# Supplementary material for: High-throughput single-molecule quantification of individual base stacking energies in nucleic acids
Source: Nat Commun. 2023 Feb 6;14:631. doi: 10.1038/s41467-023-36373-8 (PMC9902561; doi:10.1038/s41467-023-36373-8)
Supplement: Supplementary file 1 — Supplementary Information [file 41467_2023_36373_MOESM1_ESM.pdf]

Supplementary Information

High-throughput single-molecule quantification of individual base stacking energies in nucleic acids

Jibin Abraham Punnoose, Kevin J. Thomas, Arun Richard Chandrasekaran, Javier Vilcapoma, Andrew Hayden, Kacey Kilpatrick, Sweta Vangaveti, Alan Chen, Thomas Banco, Ken Halvorsen

Contents

**Supplementary Table 1:**	List of oligonucleotide sequences
**Supplementary Table 2:**	Oligonucleotide combinations for each single-molecule construct
**Supplementary Table 3**:	Construct combination to form tethers in single-molecule experiments
**Supplementary Table 4:**	Reagents used
**Supplementary Figure 1:**	Modular construct design
**Supplementary Figure 2**:	Decay plots and single-exponential fits of A|C construct at various forces
**Supplementary Figure 3**:	Decay plots and single-exponential fits of A|T construct at various forces
**Supplementary Figure 4:**	Decay plots and single-exponential fits of control construct at various forces
**Supplementary Figure 5:**	ΔG_{stack} determined for A|C and A|T base-stack at various forces
**Supplementary Figure 6**:	Tethering combinations for each stacking interaction
**Supplementary Figure 7**:	Decay plots and single-exponential fits of A|T, G|T, A|C, and G|C base-stacks at 15 pN
**Supplementary Figure 8**:	Decay plot and single-exponential fits of C|T, T|T, C|C base-stacks and control construct at 15 pN
**Supplementary Figure 9:**	Decay plot and single-exponential fits of G|A, A|A, G|G base-stacks and control construct at 15 pN
**Supplementary Figure 10**:	Tethering combinations for modified base constructs
**Supplementary Figure 11**:	Decay plots and single-exponential fits of phosphorylated and methylated A|C base-stacks and control constructs at 15 pN
**Supplementary Figure 12**:	Decay plots and single-exponential fits of FAM and ribose A|C base-stacks and control constructs at 15 pN
**Supplementary Figure 13**:	Design of DNA tetrahedron.
**Supplementary Figure 14**:	Triplicate gel pics for DNA tetrahedron at various temperatures
**Supplementary Figure 15**:	Non-denaturing PAGE confirms the ligation of the two DNA duplexes.
**Supplementary Figure 16:**	Triplicate gel pics for ligation experiments with 3 nt overhang
**Supplementary Figure 17:**	Triplicate gel pics for ligation experiments with 4 nt overhang
**Supplementary Figure 18**:	Ligation experiments with 3 nt overhang with G|A Stack vs 4 nt overhang with C|T stack
**Supplementary Figure 19**:	Potential mean force at varying simulation lengths

Supplementary Table 1. List of oligonucleotide sequences.

Name	Sequence	Length
**Backbone sequences (5′-3′) (Common set of oligos for all single-molecule constructs)**		
1. 5'Biotin	(5' 2x bio) AACATCCAATAAATCATACAGGCAAGGCAAAGAATTAGCA	40
2	AAATTAAGCAATAAAGCCTC	20
3	AGAGCATAAAGCTAAATCGGTTGTACCAAAAACATTATGACCCTGTAATACTTTTGCGGG	60
4	AGAAGCCTTTATTTCAACGCAAGGATAAAAATTTTTAGAACCCTCATATATTTTAAATGC	60
5	AATGCCTGAGTAATGTGTAGGTAAAGATTCAAAAGGGTGAGAAAGGCCGGAGACAGTCAA	60
6	ATCACCATCAATATGATATTCAACCGTTCTAGCTGATAAATTAATGCCGGAGAGGGTAGC	60
7	TATTTTTGAGAGATCTACAAAGGCTATCAGGTCATTGCCTGAGAGTCTGGAGCAAACAAG	60
8	AGAATCGATGAACGGTAATCGTAAAACTAGCATGTCAATCATATGTACCCCGGTTGATAA	60
9	TCAGAAAAGCCCCAAAAACAGGAAGATTGTATAAGCAAATATTTAAATTGTAAACGTTAA	60
10	TATTTTGTTAAAATTCGCATTAAATTTTTGTTAAATCAGCTCATTTTTTAACCAATAGGA	60
11	ACGCCATCAAAAATAATTCGCGTCTGGCCTTCCTGTAGCCAGCTTTCATCAACATTAAAT	60
12	GTGAGCGAGTAACAACCCGTCGGATTCTCCGTGGGAACAAACGGCGGATTGACCGTAATG	60
13	GGATAGGTCACGTTGGTGTAGATGGGCGCATCGTAACCGTGCATCTGCCAGTTTGAGGGG	60
14	ACGACGACAGTATCGGCCTCAGGAAGATCGCACTCCAGCCAGCTTTCCGGCACCGCTTCT	60
15	GGTGCCGGAAACCAGGCAAAGCGCCATTCGCCATTCAGGCTGCGCAACTGTTGGGAAGGG	60
16	CGATCGGTGCGGGCCTCTTCGCTATTACGCCAGCTGGCGAAAGGGGGATGTGCTGCAAGG	60
17	CGATTAAGTTGGGTAACGCCAGGGTTTTCCCAGTCACGACGTTGTAAAACGACGGCCAGT	60
18	GCCAAGCTTGCATGCCTGCAGGTCGACTCTAGAGGATCCCCGGGTACCGAGCTCGAATTC	60
19	GTAATCATGGTCATAGCTGTTTCCTGTGTGAAATTGTTATCCGCTCACAATTCCACACAA	60
20	CATACGAGCCGGAAGCATAAAGTGTAAAGCCTGGGGTGCCTAATGAGTGAGCTAACTCAC	60
21	ATTAATTGCGTTGCGCTCACTGCCCGCTTTCCAGTCGGGAAACCTGTCGTGCCAGCTGCA	60
22	TTAATGAATCGGCCAACGCGCGGGGAGAGGCGGTTTGCGTATTGGGCGCCAGGGTGGTTT	60
23	TTCTTTTCACCAGTGAGACGGGCAACAGCTGATTGCCCTTCACCGCCTGGCCCTGAGAGA	60
24	GTTGCAGCAAGCGGTCCACGCTGGTTTGCCCCAGCAGGCGAAAATCCTGTTTGATGGTGG	60
25	TTCCGAAATCGGCAAAATCCCTTATAAATCAAAAGAATAGCCCGAGATAGGGTTGAGTGT	60
26	TGTTCCAGTTTGGAACAAGAGTCCACTATTAAAGAACGTGGACTCCAACGTCAAAGGGCG	60
27	AAAAACCGTCTATCAGGGCGATGGCCCACTACGTGAACCATCACCCAAATCAAGTTTTTT	60
28	GGGGTCGAGGTGCCGTAAAGCACTAAATCGGAACCCTAAAGGGAGCCCCCGATTTAGAGC	60
29	TTGACGGGGAAAGCCGGCGAACGTGGCGAGAAAGGAAGGGAAGAAAGCGAAAGGAGCGGG	60
30	CGCTAGGGCGCTGGCAAGTGTAGCGGTCACGCTGCGCGTAACCACCACACCCGCCGCGCT	60
31	TAATGCGCCGCTACAGGGCGCGTACTATGGTTGCTTTGACGAGCACGTATAACGTGCTTT	60
32	CCTCGTTAGAATCAGAGCGGGAGCTAAACAGGAGGCCGATTAAAGGGATTTTAGACAGGA	60
33	ACGGTACGCCAGAATCCTGAGAAGTGTTTTTATAATCAGTGAGGCCACCGAGTAAAAGAG	60
34	TCTGTCCATCACGCAAATTAACCGTTGTAGCAATACTTCTTTGATTAGTAATAACATCAC	60
35	TTGCCTGAGTAGAAGAACTCAAACTATCGGCCTTGCTGGTAATATCCAGAACAATATTAC	60
36	CGCCAGCCATTGCAACAGGAAAAACGCTCATGGAAATACCTACATTTTGACGCTCAATCG	60

37	TCTGAAATGGATTATTTACATTGGCAGATTCACCAGTCACACGACCAGTAATAAAAGGGA	60
38	CATTCTGGCCAACAGAGATAGAACCCTTCTGACCTGAAAGCGTAAGAATACGTGGCACAG	60
39	ACAATATTTTTGAATGGCTATTAGTCTTTAATGCGCGAACTGATAGCCCTAAAACATCGC	60
40	CATTAAAAATACCGAACGAACCACCAGCAGAAGATAAAACAGAGGTGAGGCGGTCAGTAT	60
41	TAACACCGCCTGCAACAGTGCCACGCTGAGAGCCAGCAGCAAATGAAAAATCTAAAGCAT	60
42	CACCTTGCTGAACCTCAAATATCAAACCCTCAATCAATATCTGGTCAGTTGGCAAATCAA	60
43	CAGTTGAAAGGAATTGAGGAAGGTTATCTAAAATATCTTTAGGAGCACTAACAACTAATA	60
44	GATTAGAGCCGTCAATAGATAATACATTTGAGGATTTAGAAGTATTAGACTTTACAAACA	60
45	ATTCGACAACTCGTATTAAATCCTTTGCCCGAACGTTATTAATTTTAAAAGTTTGAGTAA	60
46	CATTATCATTTTGCGGAACAAAGAAACCACCAGAAGGAGCGGAATTATCATCATATTCCT	60
47	GATTATCAGATGATGGCAATTCATCAATATAATCCTGATTGTTTGGATTATACTTCTGAA	60
48	TAATGGAAGGGTTAGAACCTACCATATCAAAATTATTTGCACGTAAAACAGAAATAAAGA	60
49	AATTGCGTAGATTTTCAGGTTTAACGTCAGATGAATATACAGTAACAGTACCTTTTACAT	60
50	CGGGAGAAACAATAACGGATTCGCCTGATTGCTTTGAATACCAAGTTACAAAATCGCGCA	60
51	GAGGCGAATTATTCATTTCAATTACCTGAGCAAAAGAAGATGATGAAACAAACATCAAGA	60
52	AAACAAAATTAATTACATTTAACAATTTCATTTGAATTACCTTTTTTAATGGAAACAGTA	60
53	CATAAATCAATATATGTGAGTGAATAACCTTGCTTCTGTAAATCGTCGCTATTAATTAAT	60
54	TTTCCCTTAGAATCCTTGAAAACATAGCGATAGCTTAGATTAAGACGCTGAGAAGAGTCA	60
55	ATAGTGAATTTATCAAAATCATAGGTCTGAGAGACTACCTTTTTAACCTCCGGCTTAGGT	60
56	TGGGTTATATAACTATATGTAAATGCTGATGCAAATCCAATCGCAAGACAAAGAACGCGA	60
57	GAAAACTTTTTCAAATATATTTTAGTTAATTTCATCTTCTGACCTAAATTTAATGGTTTG	60
58	AAATACCGACCGTGTGATAAATAAGGCGTTAAATAAGAATAAACACCGGAATCATAATTA	60
59	CTAGAAAAAGCCTGTTTAGTATCATATGCGTTATACAAATTCTTACCAGTATAAAGCCAA	60
60	CGCTCAACAGTAGGGCTTAATTGAGAATCGCCATATTTAACAACGCCAACATGTAATTTA	60
61	GGCAGAGGCATTTTCGAGCCAGTAATAAGAGAATATAAAGTACCGACAAAAGGTAAAGTA	60
62	ATTCTGTCCAGACGACGACAATAAACAACATGTTCAGCTAATGCAGAACGCGCCTGTTTA	60
63	TCAACAATAGATAAGTCCTGAACAAGAAAAATAATATCCCATCCTAATTTACGAGCATGT	60
64	AGAAACCAATCAATAATCGGCTGTCTTTCCTTATCATTCCAAGAACGGGTATTAAACCAA	60
65	GTACCGCACTCATCGAGAACAAGCAAGCCGTTTTTATTTTCATCGTAGGAATCATTACCG	60
66	CGCCCAATAGCAAGCAAATCAGATATAGAAGGCTTATCCGGTATTCTAAGAACGCGAGGC	60
67	GTTTTAGCGAACCTCCCGACTTGCGGGAGGTTTTGAAGCCTTAAATCAAGATTAGTTGCT	60
68	ATTTTGCACCCAGCTACAATTTTATCCTGAATCTTACCAACGCTAACGAGCGTCTTTCCA	60
69	GAGCCTAATTTGCCAGTTACAAAATAAACAGCCATATTATTTATCCCAATCCAAATAAGA	60
70	AACGATTTTTTGTTTAACGTCAAAAATGAAAATAGCAGCCTTTACAGAGAGAATAACATA	60
71	AAAACAGGGAAGCGCATTAGACGGGAGAATTAACTGAACACCCTGAACAAAGTCAGAGGG	60
72	TAATTGAGCGCTAATATCAGAGAGATAACCCACAAGAATTGAGTTAAGCCCAATAATAAG	60
73	AGCAAGAAACAATGAAATAGCAATAGCTATCTTACCGAAGCCCTTTTTAAGAAAAGTAAG	60
74	CAGATAGCCGAACAAAGTTACCAGAAGGAAACCGAGGAAACGCAATAATAACGGAATACC	60
75	CAAAAGAACTGGCATGATTAAGACTCCTTATTACGCAGTATGTTAGCAAACGTAGAAAAT	60
76	ACATACATAAAGGTGGCAACATATAAAAGAAACGCAAAGACACCACGGAATAAGTTTATT	60
77	TTGTCACAATCAATAGAAAATTCATATGGTTTACCAGCGCCAAAGACAAAAGGGCGACAT	60

78	TCAACCGATTGAGGGAGGGAAGGTAAATATTGACGGAAATTATTCATTAAAGGTGAATTA	60
79	TCACCGTCACCGACTTGAGCCATTTGGGAATTAGAGCCAGCAAAATCACCAGTAGCACCA	60
80	TTACCATTAGCAAGGCCGGAAACGTCACCAATGAAACCATCGATAGCAGCACCGTAATCA	60
81	GTAGCGACAGAATCAAGTTTGCCTTTAGCGTCAGACTGTAGCGCGTTTTCATCGGCATTT	60
82	TCGGTCATAGCCCCCTTATTAGCGTTTGCCATCTTTTCATAATCAAAATCACCGGAACCA	60
83	GAGCCACCACCGGAACCGCCTCCCTCAGAGCCGCCACCCTCAGAACCGCCACCCTCAGAG	60
84	CCACCACCCTCAGAGCCGCCACCAGAACCACCACCAGAGCCGCCGCCAGCATTGACAGGA	60
85	GGTTGAGGCAGGTCAGACGATTGGCCTTGATATTCACAAACAAATAAATCCTCATTAAAG	60
86	CCAGAATGGAAAGCGCAGTCTCTGAATTTACCGTTCCAGTAAGCGTCATACATGGCTTTT	60
87	GATGATACAGGAGTGTACTGGTAATAAGTTTTAACGGGGTCAGTGCCTTGAGTAACAGTG	60
88	CCCGTATAAACAGTTAATGCCCCCTGCCTATTTCGGAACCTATTATTCTGAAACATGAAA	60
89	GTATTAAGAGGCTGAGACTCCTCAAGAGAAGGATTAGGATTAGCGGGGTTTTGCTCAGTA	60
90	CCAGGCGGATAAGTGCCGTCGAGAGGGTTGATATAAGTATAGCCCGGAATAGGTGTATCA	60
91	CCGTACTCAGGAGGTTTAGTACCGCCACCCTCAGAACCGCCACCCTCAGAACCGCCACCC	60
92	TCAGAGCCACCACCCTCATTTTCAGGGATAGCAAGCCCAATAGGAACCCATGTACCGTAA	60
93	CACTGAGTTTCGTCACCAGTACAAACTACAACGCCTGTAGCATTCCACAGACAGCCCTCA	60
94	TAGTTAGCGTAACGATCTAAAGTTTTGTCGTCTTTCCAGACGTTAGTAAATGAATTTTCT	60
95	GTATGGGATTTTGCTAAACAACTTTCAACAGTTTCAGCGGAGTGAGAATAGAAAGGAACA	60
96	ACTAAAGGAATTGCGAATAATAATTTTTTCACGTTGAAAATCTCCAAAAAAAAAGGCTCCA	60
97	AAAGGAGCCTTTAATTGTATCGGTTTATCAGCTTGCTTTCGAGGTGAATTTCTTAAACAG	60
98	CTTGATACCGATAGTTGCGCCGACAATGACAACAACCATCGCCCACGCATAACCGATATA	60
99	TTCGGTCGCTGAGGCTTGCAGGGAGTTAAAGGCCGCTTTTGCGGGATCGTCACCCTCAGC	60
100	AGCGAAAGACAGCATCGGAACGAGGGTAGCAACGGCTACAGAGGCTTTGAGGACTAAAGA	60
101	CTTTTTCATGAGGAAGTTTCCATTAAACGGGTAAAATACGTAATGCCACTACGAAGGCAC	60
102	CAACCTAAAACGAAAGAGGCAAAAGAATACACTAAAACACTCATCTTTGACCCCCAGCGA	60
103	TTATACCAAGCGCGAAACAAAGTACAACGGAGATTTGTATCATCGCCTGATAAATTGTGT	60
104	CGAAATCCGCGACCTGCTCCATGTTACTTAGCCGGAACGAGGCGCAGACGGTCAATCATA	60
105	AGGGAACCGAACTGACCAACTTTGAAAGAGGACAGATGAACGGTGTACAGACCAGGCGCA	60
106	TAGGCTGGCTGACCTTCATCAAGAGTAATCTTGACAAGAACCGGATATTCATTACCCAAA	60
107	TCAACGTAACAAAGCTGCTCATTCAGTGAATAAGGCTTGCCCTGACGAGAAACACCAGAA	60
108	CGAGTAGTAAATTGGGCTTGAGATGGTTTAATTTCAACTTTAATCATTGTGAATTACCTT	60
109	ATGCGATTTTAAGAACTGGCTCATTATACCAGTCAGGACGTTGGGAAGAAAAATCTACGT	60
110	TAATAAAACGAACTAACGGAACAACATTATTACAGGTAGAAAGATTCATCAGTTGAGATT	60
111	TAGGAATACCACATTCAACTAATGCAGATACATAACGCCAAAAGGAATTACGAGGCATAG	60
112	TAAGAGCAACACTATCATAACCCTCGTTTACCAGACGACGATAAAAACCAAAATAGCGAG	60
113	AGGCTTTTGCAAAAGAAGTTTTGCCAGAGGGGGTAATAGTAAAATGTTTAGACTGGATAG	60
114	CGTCCAATACTGCGGAATCGTCATAAATATTCATTGAATCCCCCTCAAATGCTTTAAACA	60
115	GTTCAGAAAACGAGAATGACCATAAATCAAAAATCAGGTCTTTACCCTGACTATTATAGT	60
116	CAGAAGCAAAGCGGATTGCATCAAAAAGATTAAGAGGAAGCCCGAAAGACTTCAAATATC	60
117	GCGTTTTAATTCGAGCTTCAAAGCGAACCAGACCGGAAGCAAACTCCAACAGGTCAGGAT	60
118	TAGAGAGTACCTTTAATTGCTCCTTTTGATAAGAGGTCATTTTTGCGGATGGCTTAGAGC	60

119	TTAATTGCTGAATATAATGCTGTAGCTCAACATGTTTTAAATATGCAACTAAAGTACGGT	60
120	GTCTGGAAGTTTCATTCCATATAACAGTTGATTCCCAATTCTGCGAACGAGTAGATTTAG	60
121	TTTGACCATTAGATACATTTCGCAAATGGTCAATAACCTGTTTAGCTAT	49
122	ATTTTCATTTGGGGCGCGAGCTGAAAAGGT	30
**Sequence used in specific combination for each construct (5′-3′)<br>Overhanging regions underlined, spacer T are marked in blue, and stacking bases in red**		
OH-A	GGCATCAATTCTACTAATAGTAGTAGCATTCCGTGCCTGTGAACGAGCTGCCCCATGGCA	60
OH-G	GGCATCAATTCTACTAATAGTAGTAGCATTCCGTGCCTGTGAACGAGCTGCCCCATGGCG	60
OH-C	GGCATCAATTCTACTAATAGTAGTAGCATTCCGTGCCTGTGAACGAGCTGCCCCATGGCC	60
OH-T	GGCATCAATTCTACTAATAGTAGTAGCATTCCGTGCCTGTGAACGAGCTGCCCCATGGCT	60
A:A-C	CCGCTGCATGCCATGGGGCAGCTCGTTCACAGGCACGG	38
G:A-C	CCGCTGCACGCCATGGGGCAGCTCGTTCACAGGCACGG	38
C:A-C	CCGCTGCAGGCCATGGGGCAGCTCGTTCACAGGCACGG	38
T:A-C	CCGCTGCAAGCCATGGGGCAGCTCGTTCACAGGCACGG	38
T: Sp-A-C	CCGCTGCATTTAGCCATGGGGCAGCTCGTTCACAGGCACGG	41
A: G-T	TGCAGCGGTGCCATGGGGCAGCTCGTTCACAGGCACGG	38
G: G-T	TGCAGCGGCGCCATGGGGCAGCTCGTTCACAGGCACGG	38
C: G-T	TGCAGCGGGGCCATGGGGCAGCTCGTTCACAGGCACGG	38
T: G-T	TGCAGCGGAGCCATGGGGCAGCTCGTTCACAGGCACGG	38
T:Sp-G-T	TGCAGCGGTTTAGCCATGGGGCAGCTCGTTCACAGGCACGG	41
G: C-A	ACGTCGCCCGCCATGGGGCAGCTCGTTCACAGGCACGG	38
T: Sp-C-A	ACGTCGCCTTTAGCCATGGGGCAGCTCGTTCACAGGCACGG	41
A: T-G	GGCGACGTTGCCATGGGGCAGCTCGTTCACAGGCACGG	38
G: T-G	GGCGACGTCGCCATGGGGCAGCTCGTTCACAGGCACGG	38
T: Sp-T-G	GGCGACGTTTTAGCCATGGGGCAGCTCGTTCACAGGCACGG	41
**Sequence used for constructs with modified stacks (5′-3′)<br>Overhanging regions underlined, spacer T are marked in blue, and stacking bases in red**		
T:Sp-A-C^{p}	/5Phos/CCGCTGCATTTAGCCATGGGGCAGCTCGTTCACAGGCACGG	41
T:Sp-A-C^{FAM}	/56-FAM/CCGCTGCATTTAGCCATGGGGCAGCTCGTTCACAGGCACGG	41
T:Sp-A-^{Me}C	/5Me-dC/CGCTGCATTTAGCCATGGGGCAGCTCGTTCACAGGCACGG	41
OH-rA	GGCATCAATTCTACTAATAGTAGTAGCATTCCGTGCCTGTGAACGAGCTGCCCCATGGCrA	60
**Sequence used for constructing DNA tetrahedron (5′-3′)**		
L	AGGCACCATCGTAGGTTTTTCTTGCCAGGCACCATCGTAGGTTTTTCTTGCCAGGCACCATCG TAGGTTTTTCTTGCC	78
M1	TAGCAACCTGCCTGGCAAGCCTACGATGGACACGGTATCGCA	42
S1	ATACCGTGTGGTTGCTATGCG	21
M2	AGCAACCTGCCTGGCAAGCCTACGATGGACACGGTATCGCA	41
S2	TACCGTGTGGTTGCTATGCG	20
**Sequences used for Ligation experiments (5′-3′)<br>Overhanging regions underlined and stacking bases are shown in red**		
20 AG	TATGTGTCTTCTCAGTAGTG	20
3 + 20 AG	/5Phos/ACTCACTACTGAGAAGACACATA	23
4 + 20 AG	/5Phos/ACCTCACTACTGAGAAGACACATA	24

30 AG	ATACTTATACAGCTTATTGACCATTTGCGG	30
3 + 30 AG	/5Phos/AGTCCGCAAATGGTCAATAAGCTGTATAAGTAT	33
4 + 30 AG	/5Phos/AGGTCCGCAAATGGTCAATAAGCTGTATAAGTAT	34
20 AT	TATGTGTCTTCTCAGTAGTT	20
3 + 20 AT	/5Phos/ACTAACTACTGAGAAGACACATA	23
4 + 20 AT	/5Phos/ACCTAACTACTGAGAAGACACATA	24
30 AT	ATACTTATACAGCTTATTGACCATTTGCGT	30
3 + 30 AT	/5Phos/AGTACGCAAATGGTCAATAAGCTGTATAAGTAT	33
4 + 30 AT	/5Phos/AGGTACGCAAATGGTCAATAAGCTGTATAAGTAT	34

Supplementary Table 2: Oligonucleotide combinations for each single-molecule construct.

Construct #	Oligo Mix (1:15:50 mole ratio)
1	Oligos 1-122, OH-A, A:A-C
2	Oligos 1-122, OH-G, G:A-C
3	Oligos 1-122, OH-C, C:A-C
4	Oligos 1-122, OH-T, T:A-C
5	Oligos 1-122, OH-T, T:Sp-G-T
6	Oligos 1-122, OH-A, A:G-T
7	Oligos 1-122, OH-G, G:G-T
8	Oligos 1-122, OH-C, C:G-T
9	Oligos 1-122, OH-T, T:G-T
10	Oligos 1-122, OH-T, T:Sp-A-C
11	Oligos 1-122, OH-G, G:T-G
12	Oligos 1-122, OH-A, A:T-G
13	Oligos 1-122, OH-T, T:Sp-C-A
14	Oligos 1-122, OH-G, G:C-A
15	Oligos 1-122, OH-T, T:Sp-T-G
16	Oligos 1-122, OH-T, T:Sp-A-C^{p}
17	Oligos 1-122, OH-T, T:Sp-A-C^{me}
18	Oligos 1-122, OH-T, T:Sp-A-C^{FAM}
19	Oligos 1-122, OH-rA, A:T-G

Supplementary Table 3: Construct combination to form tethers in single-molecule experiments**.**

**Tether ID**	**Stacking Combination (5′|3′)**	**Construct combinations to form tether**
1	A|T	1 & 5
2	G|T	2 & 5
3	C|T	3 & 5
4	T|T	4 & 5
5	A|C	6 & 10
6	G|C	7 & 10
7	C|C	8 & 10
8	Control for (A|T, G|T, C|T, T|T, A|C, G|C, C|C)	5 & 10
9	G|A	11 & 13
10	A|A	12 & 13
11	G|G	14 & 15
12	Control for (G|A, A|A, G|G)	13 & 15
13	A| ^{p}C	16 & 6
14	Control for A|^{p}C	16 & 5
15	A| ^{me}C	17 & 6
16	Control A|^{me}C	17 & 5
17	A ^{FAM}|C	18 & 6
18	Control A|^{FAM}C	18 & 5
19	rA|C	19 & 6
8	Control for rA|C	5 & 10

Supplementary Table 4: Reagents used.

Reagent	Supplier	Catalog #
Nuclease-free water	Invitrogen	10977-015
40% Polyacrylamide solution (29:1 acrylamide/bisacrylamide)	National Diagnostics	EC-852
GelRed (10,000× in water)	Biotium	41003
Ammonium persulfate (APS)	Fisher Scientific	BP179-100
Tetramethylethylenediamine (TEMED)	Thermo Scientific	PI17919
10 bp DNA ladder	ThermoFisher Scientific	SM1313
50 bp DNA ladder	New England Biolabs	N3236S
Bromophenol blue	Fisher Scientific	AAA1846918
Glycerol	Hampton Research	HR2-623
Tris base	VWR	97061-794
Acetic acid (1 M solution)	Fisher Scientific	S25840A
EDTA (0.5 M solution, pH 8)	VWR	BDH7830-1
Magnesium acetate	Fisher Scientific	BP215-500
Streptavidin coated microspheres (Dynabeads)	Thermo Fisher	65306
Tween 20	Millipore	655204
Phosphate buffered saline (PBS)	Fisher	BP3991
BtsCI enzyme	New England Biolabs	R0647S
Streptavidin	Amresco	E497
T4 DNA ligase buffer	New England Biolabs	B0202SVIAL
T4 DNA ligase	New England Biolabs	M0202S
ATP	New England Biolabs	P0756
Bovine serum albumin (BSA)	New England Biolabs	B9000

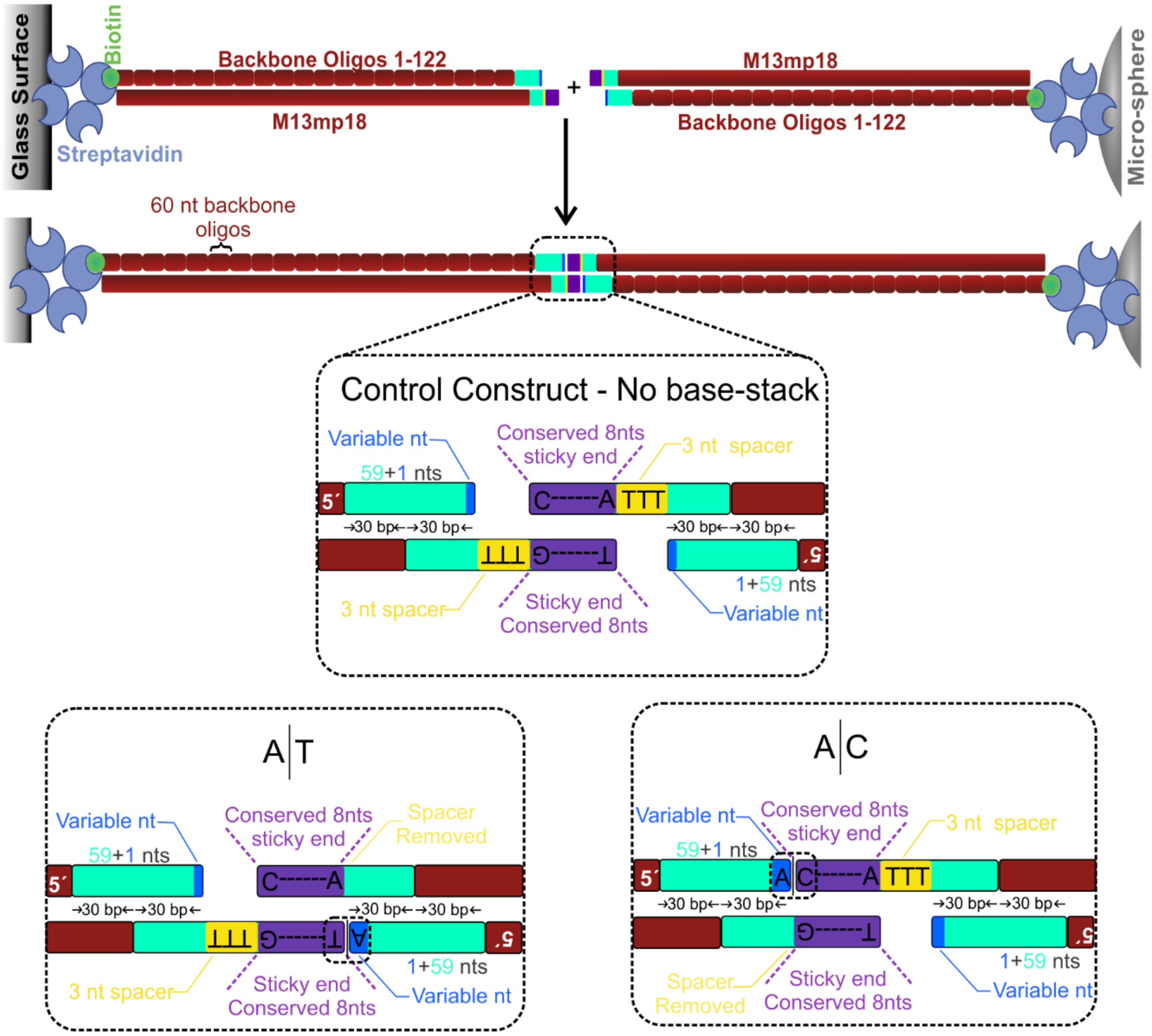

Supplementary Figure 1: Modular construct design. The central duplex (purple) has 4 nucleotides in its termini, which can interface with the variable region (blue) to form the base-stack of interest in the absence of 3 nt spacer nucleotides (yellow). The enlarged central region shows the tethers that has an A|T and an A|C base-stack and the control construct without any base-stacks. All these constructs have the same central duplex forming the same base-pairs, thus the difference in the tether strength is due to the base-stack of interest.

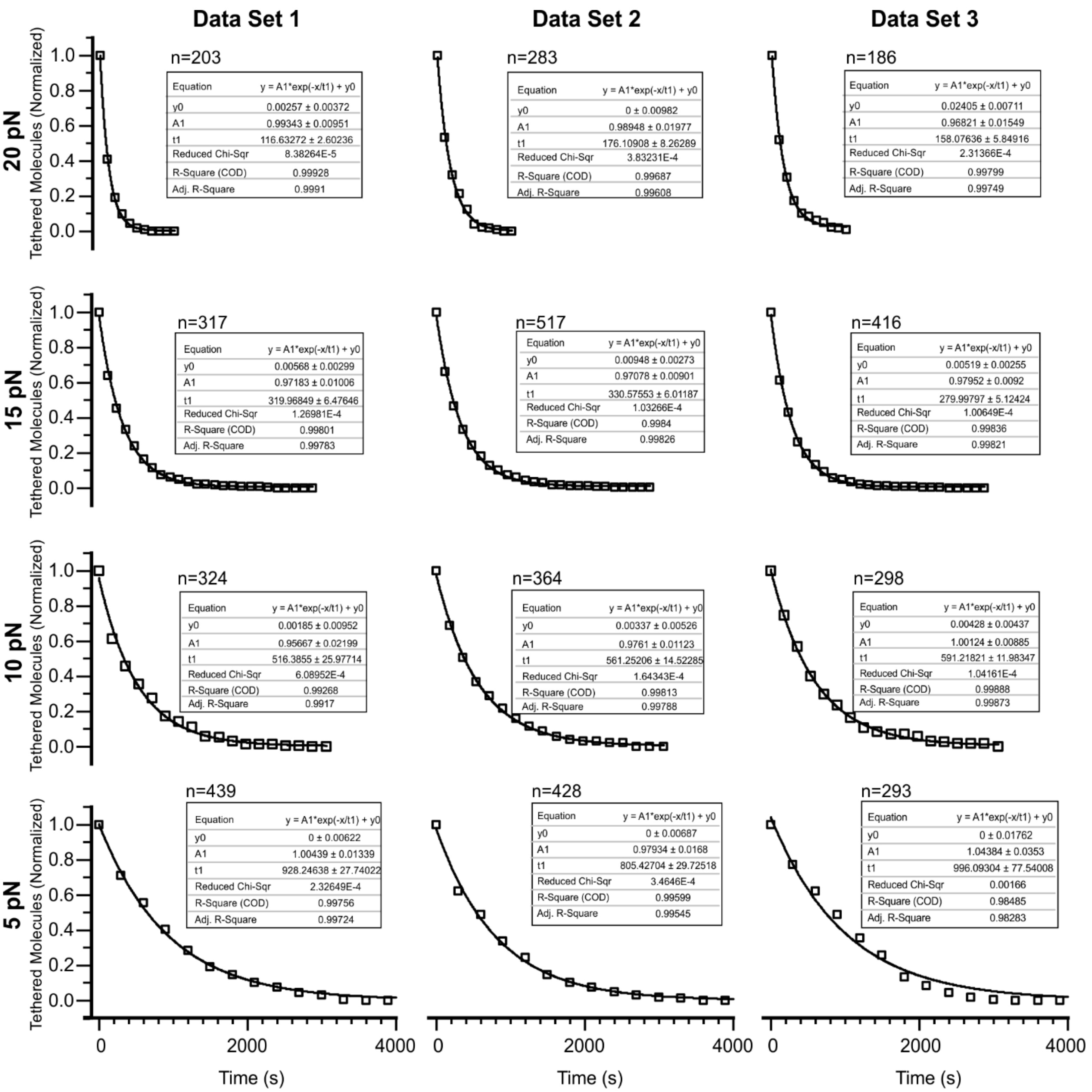

Supplementary Figure 2: Decay plots and single-exponential fits of A|C construct at various forces.

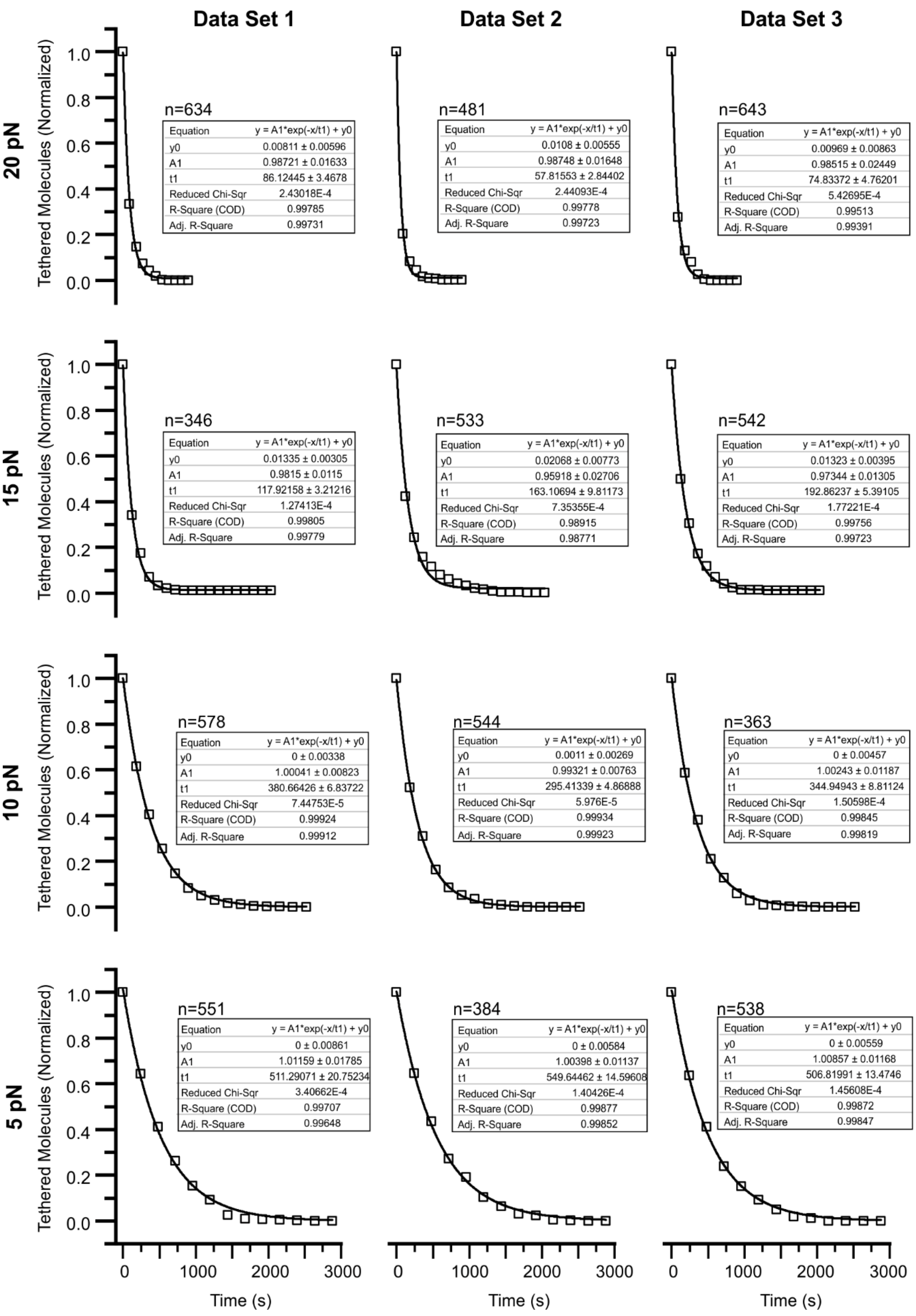

Supplementary Figure 3: **Decay plots and single-exponential fits of A|T construct at various forces.**

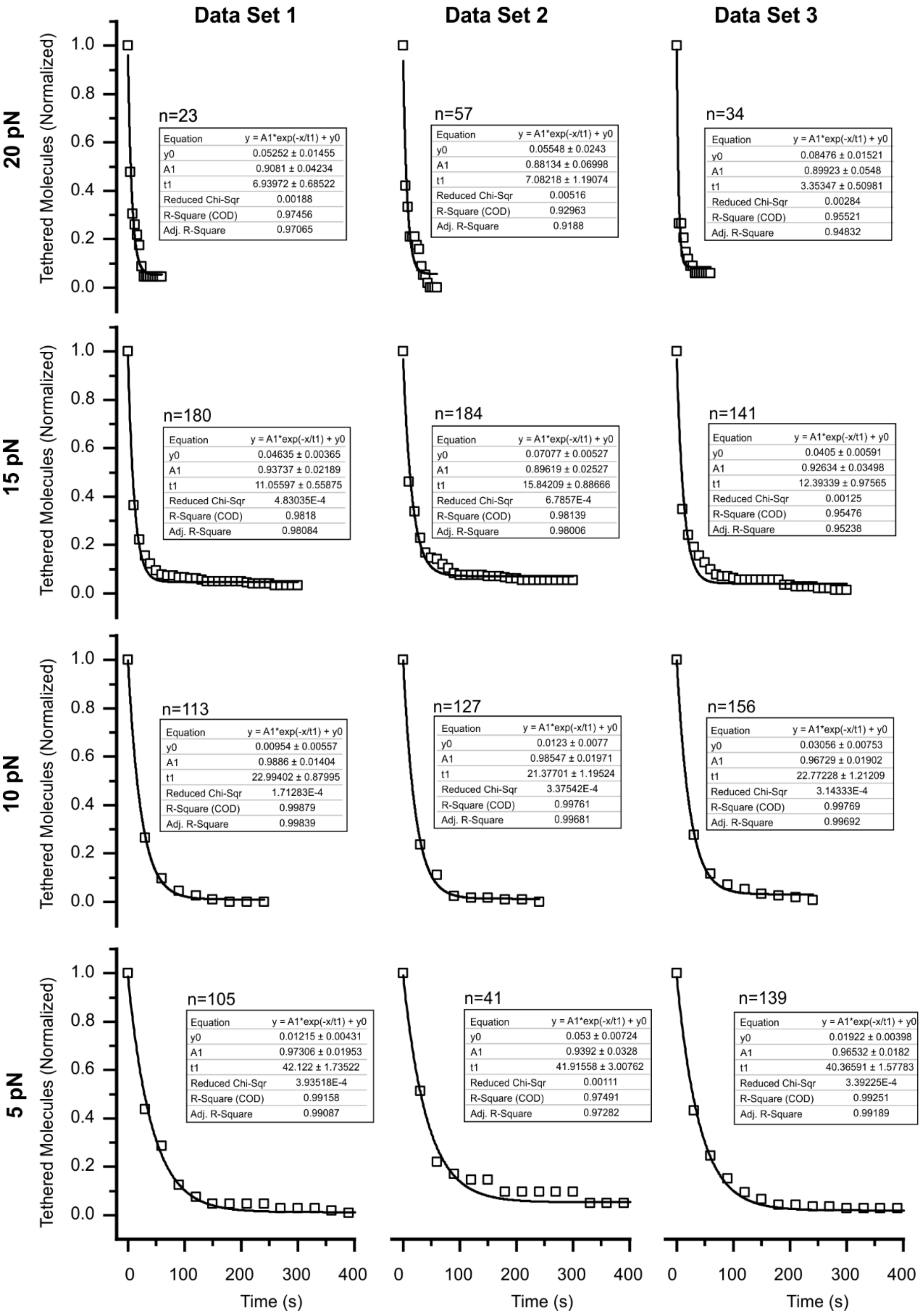

Supplementary Figure 4: Decay plots and single-exponential fits of control construct at various forces.

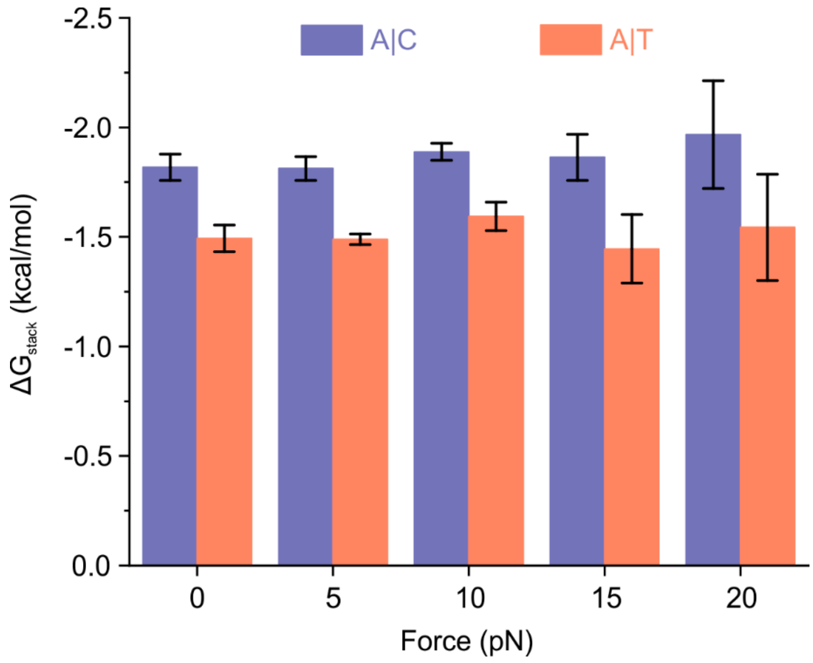

Supplementary Figure 5: ΔG_{stack} determined for A|C and A|T base-stack at various forces. ΔG_{stack} at thermal equilibrium (zero force) was obtained from fitting Force - ΔG_{stack} data with Bell-Evans model (Figure 3c). Data is calculated from mean values +/- propagated error.

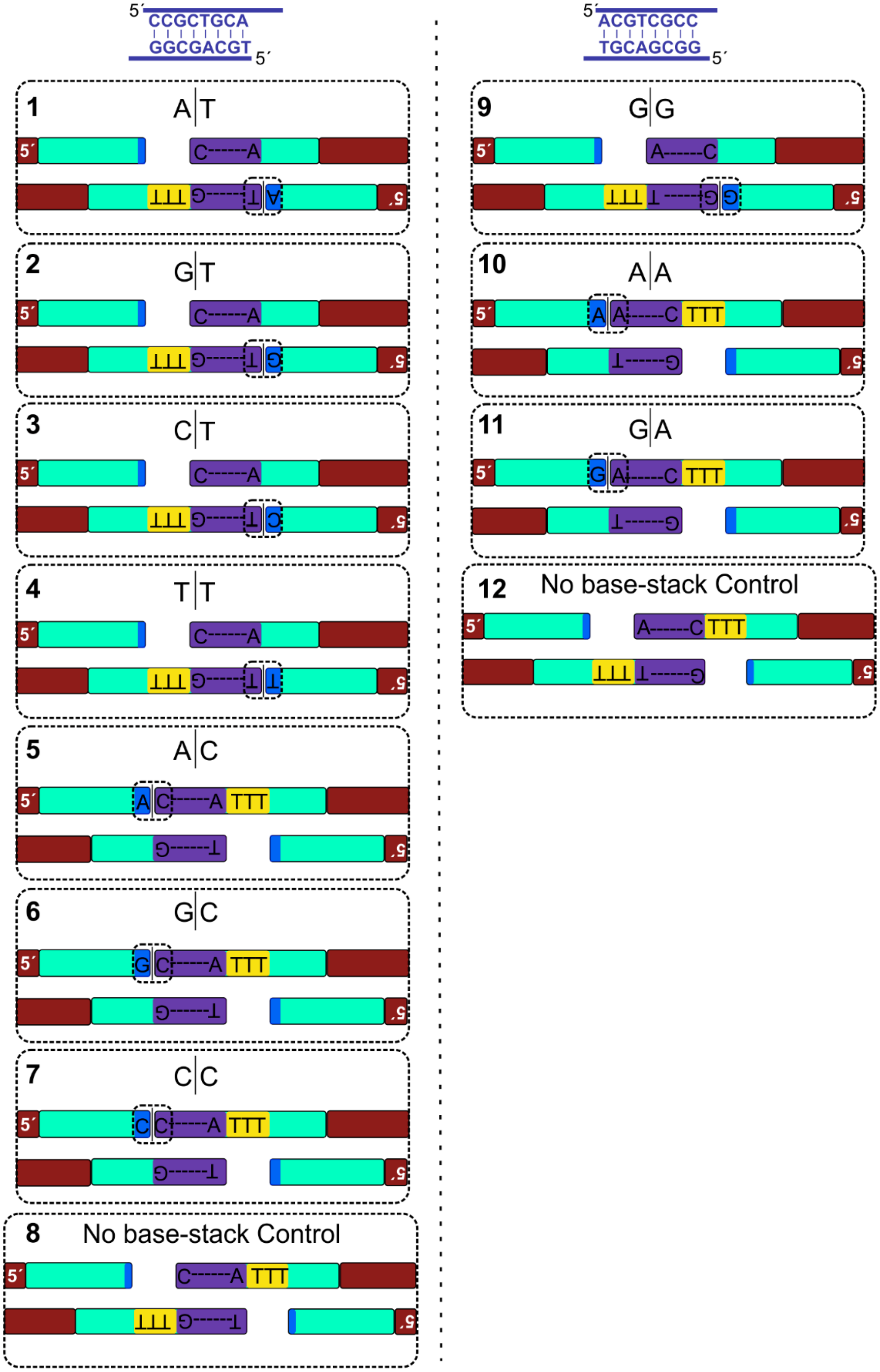

Supplementary Figure 6: **Tethering combinations for each stacking interaction.** The central duplex is conserved in all cases, but polarity is revered for the constructs in right to achieve all the combinations. The control constructs with both polarity is shown below on both left and right panel. The number on the top left of each box represents tether ID same as in Table S3.

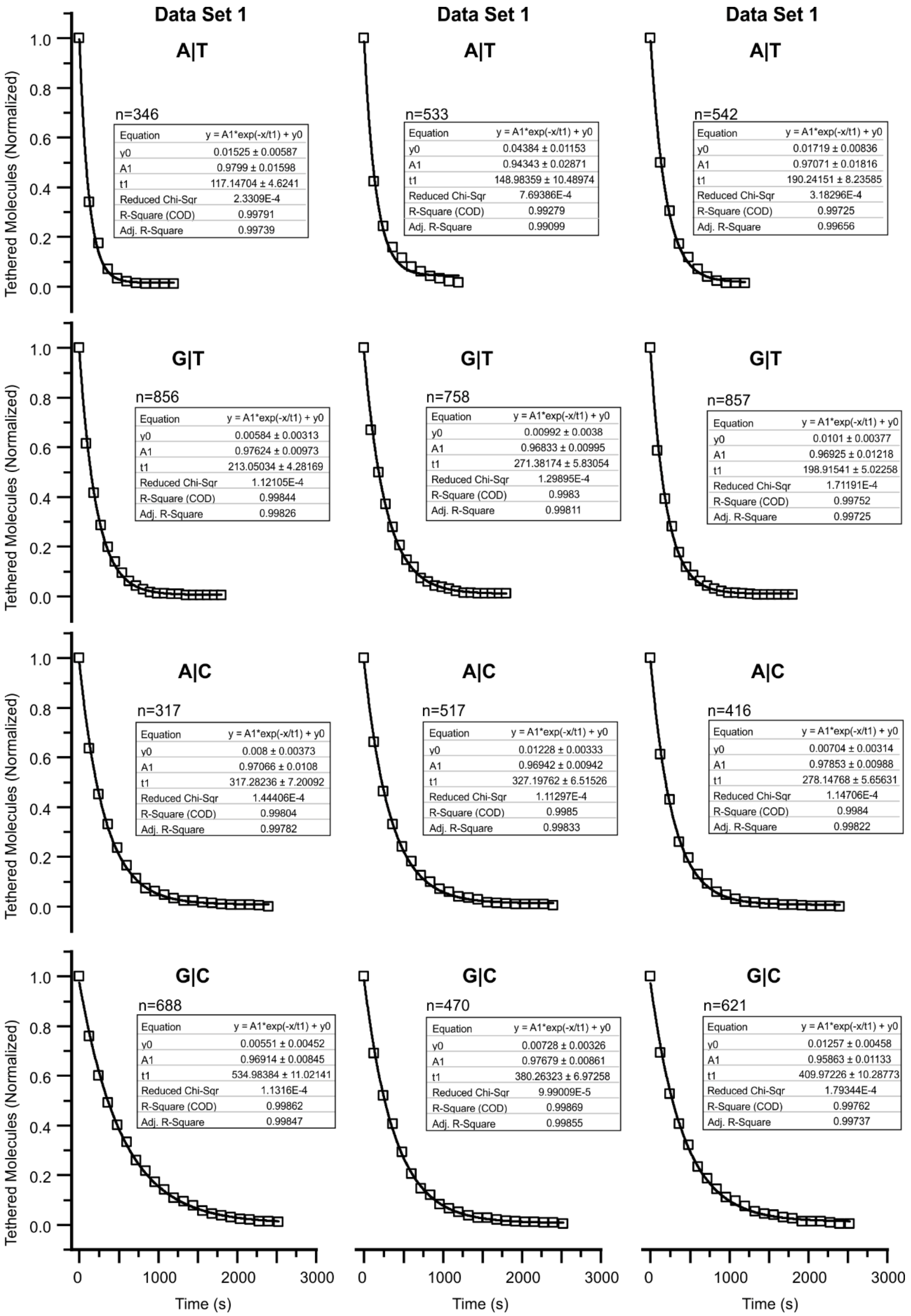

Supplementary Figure 7: **Decay plots and single-exponential fits of A|T, G|T, A|C and G|C base-stacks at 15 pN.**

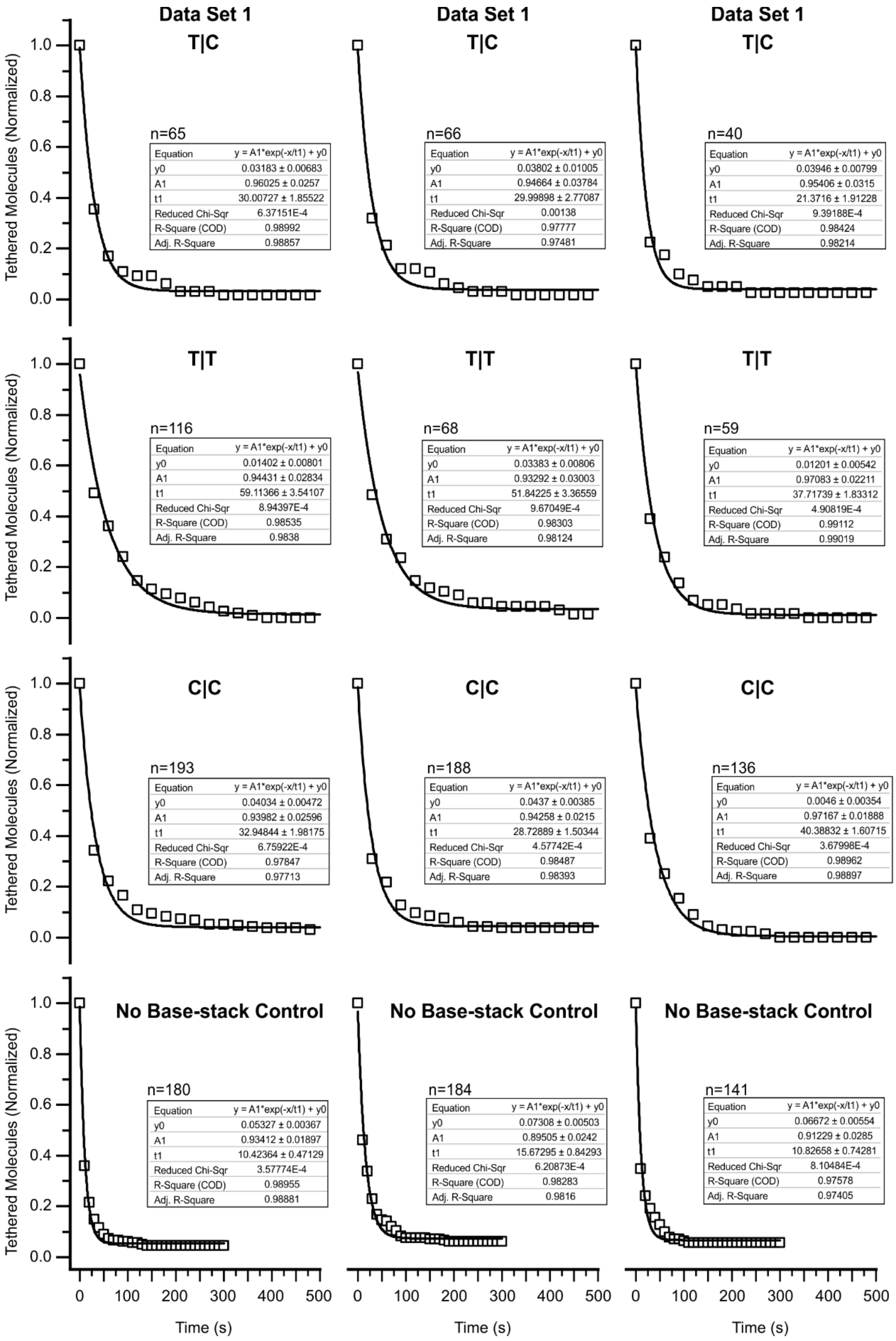

Supplementary Figure 8: **Decay plots and single-exponential fits of C|T, T|T, C|C base-stacks and control construct at 15 pN.**

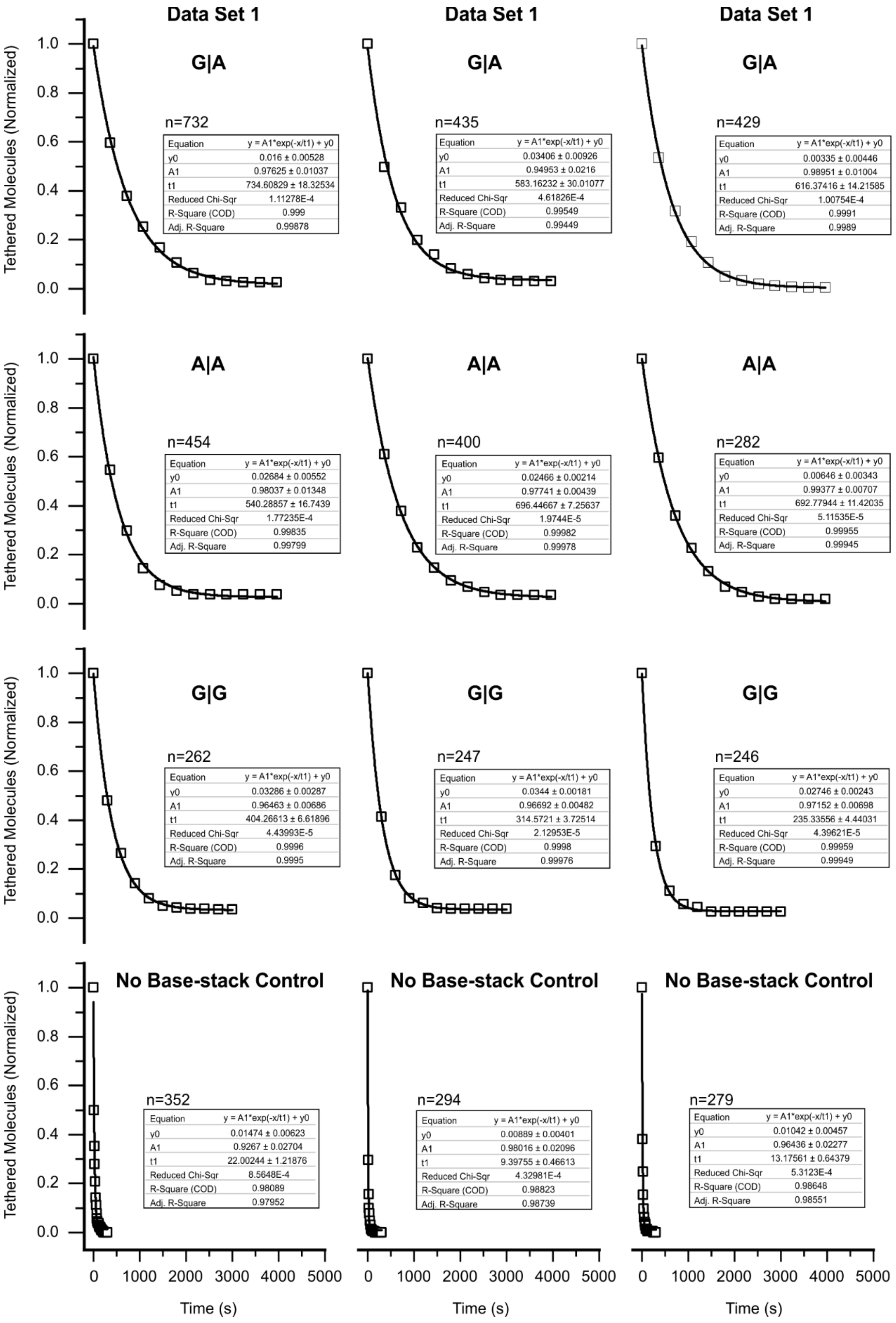

Supplementary Figure 9: **Decay plots and single-exponential fits of G|A, A|A, G|G base-stacks and control construct at 15 pN.**

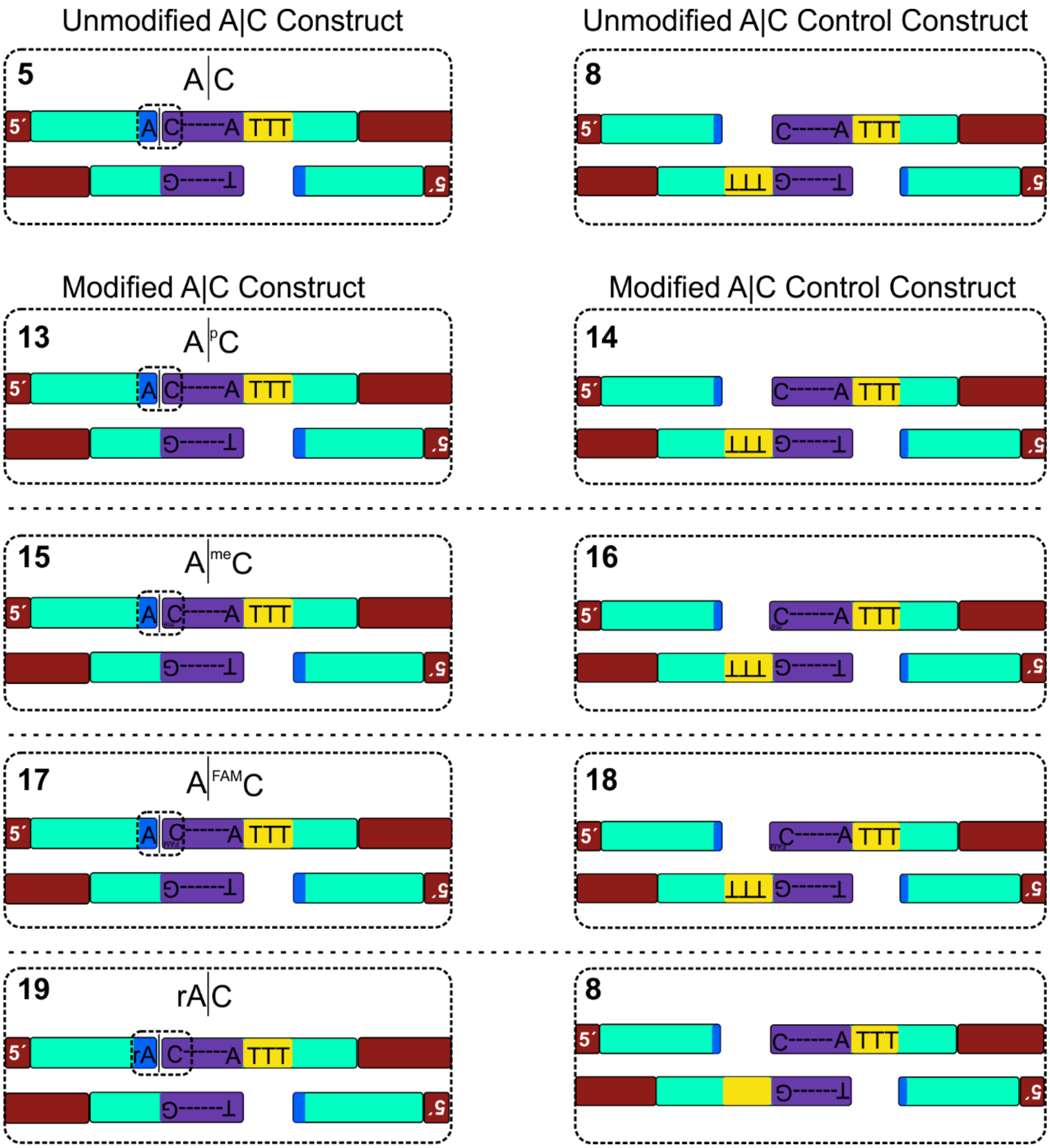

Supplementary Figure 10: **Tethering combinations for modified base constructs.** The number on the top left of each box represents tether ID same as in Table S3.

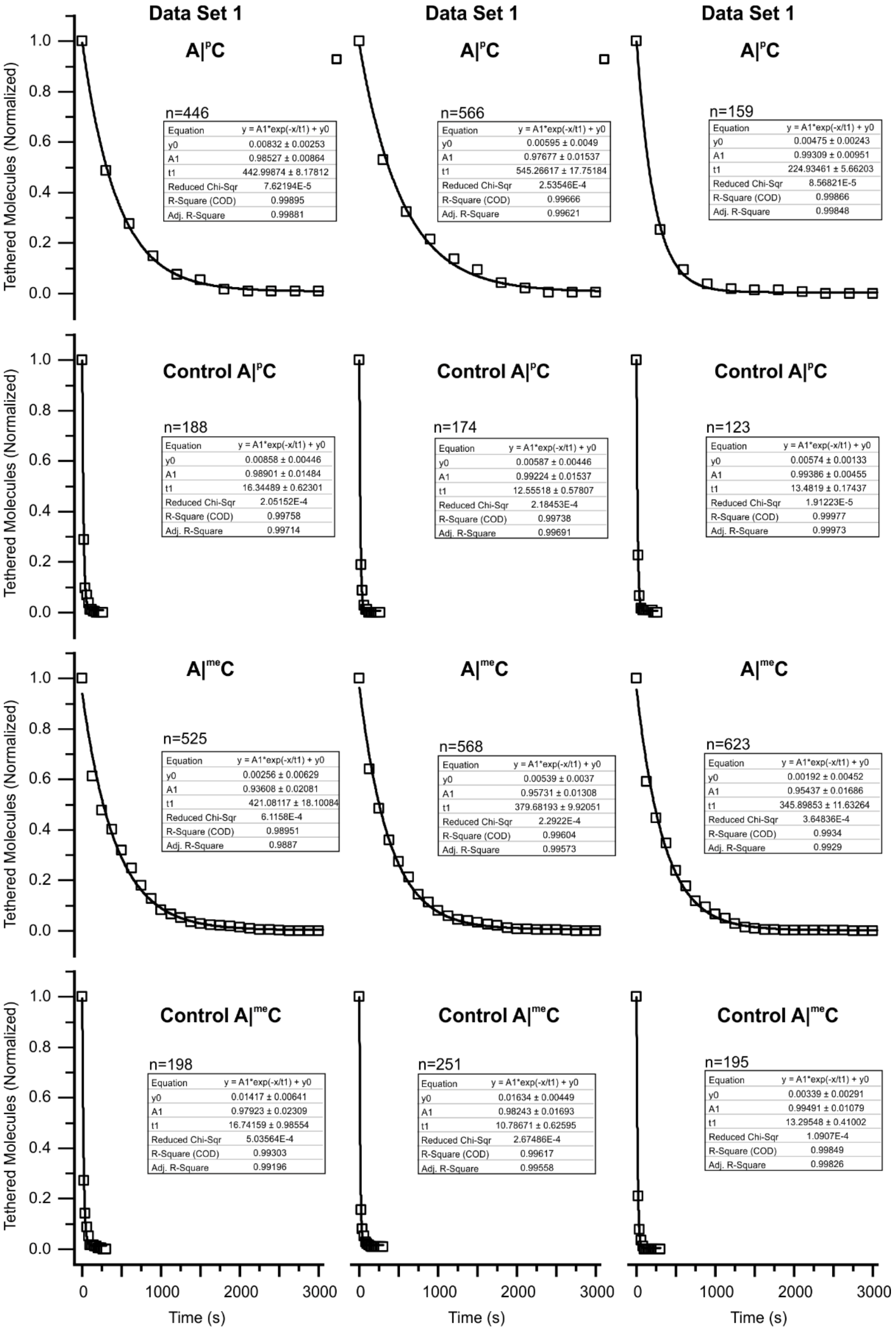

Supplementary Figure 11: **Decay plots and single-exponential fits of phosphorylated and methylated A|C base-stacks and control constructs at 15 pN.**

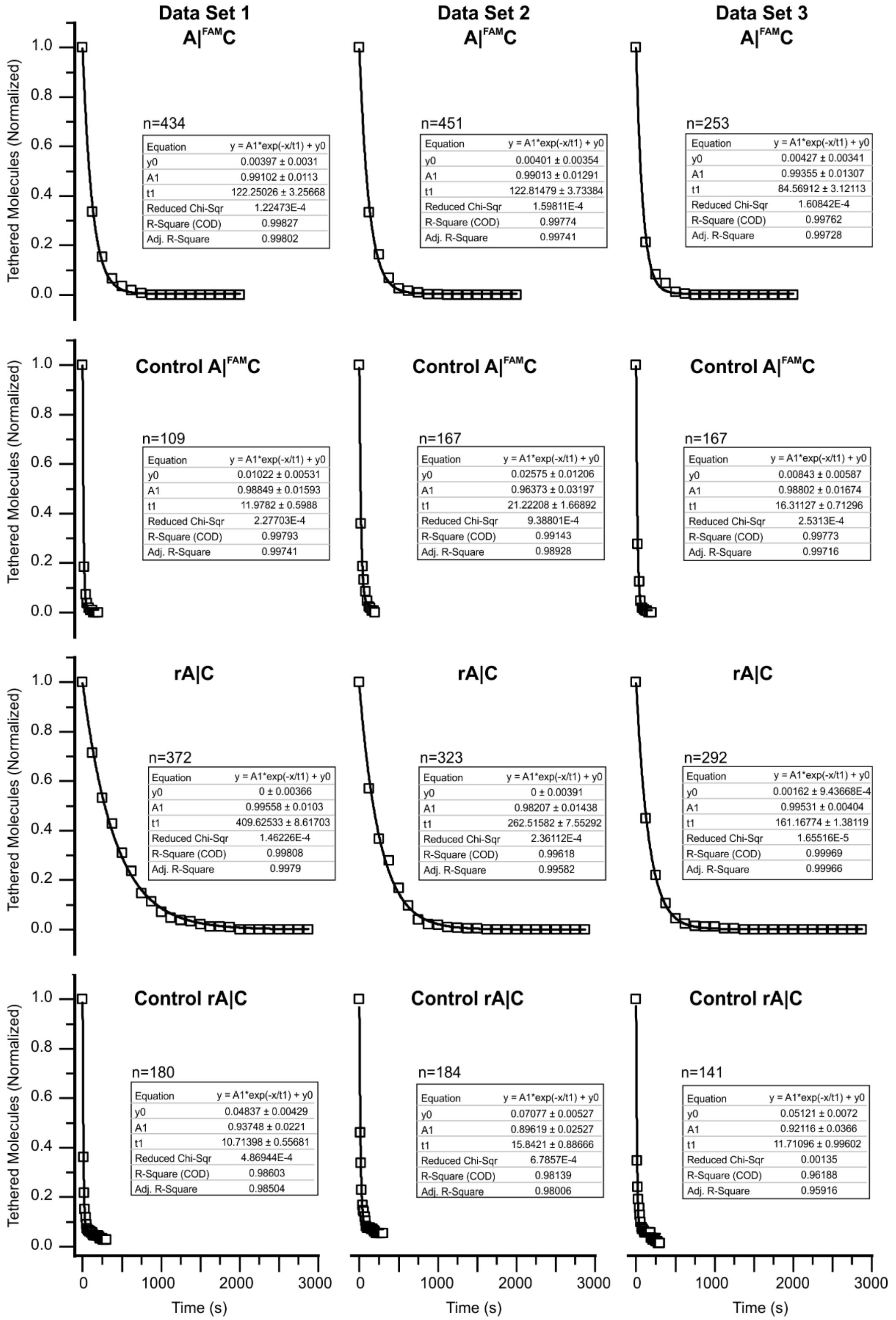

Supplementary Figure 12: **Decay plots and single-exponential fits of FAM and ribose A|C base-stacks and control constructs at 15 pN.**

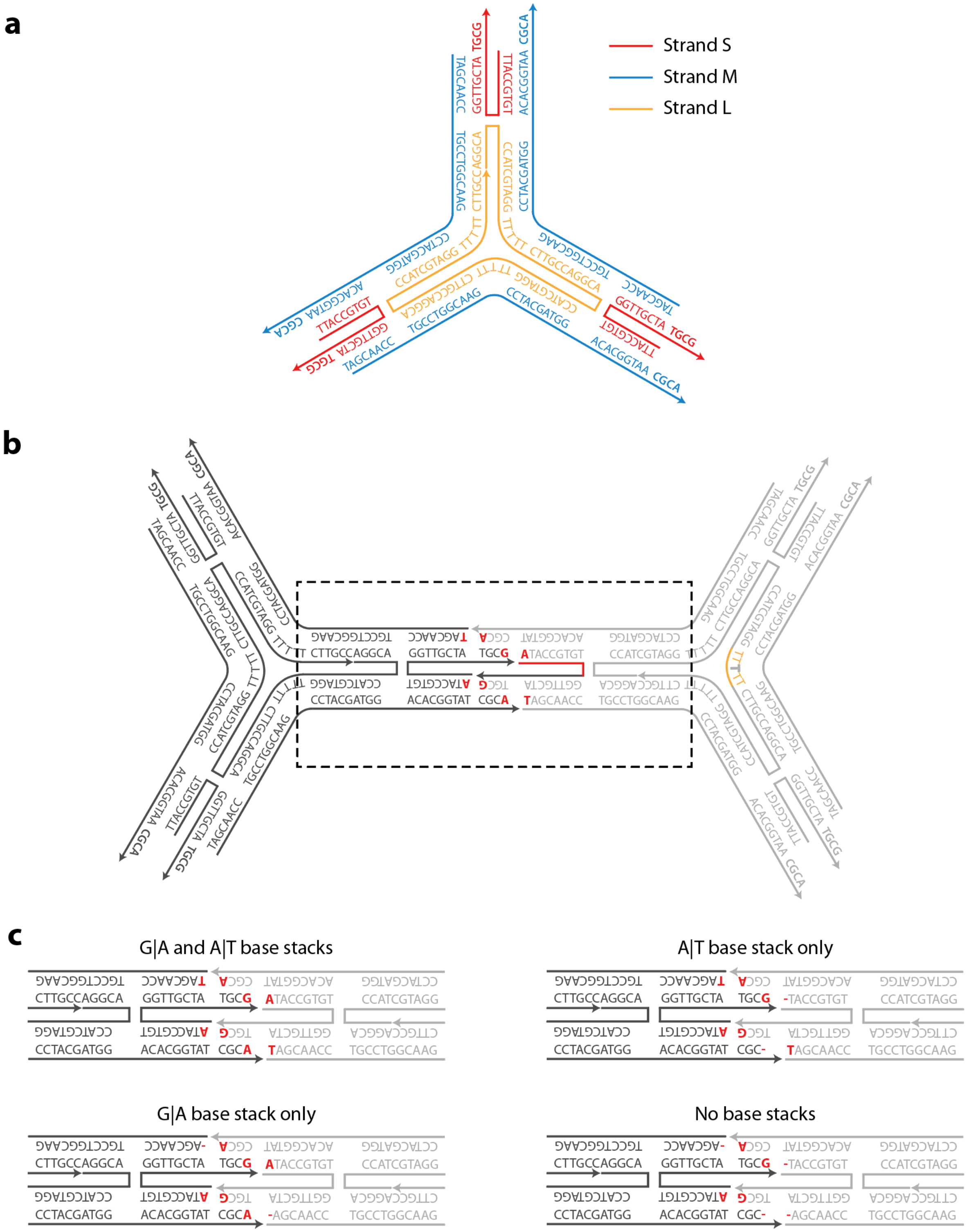

Supplementary Figure 13: **Design of DNA tetrahedron.** (a) Design and sequences of the 3-point-star motif. (b) Illustration showing the interface of double cohesion with two sticky ends and corresponding base stacks. (c) Illustration showing the different base stack combinations tested in this study.

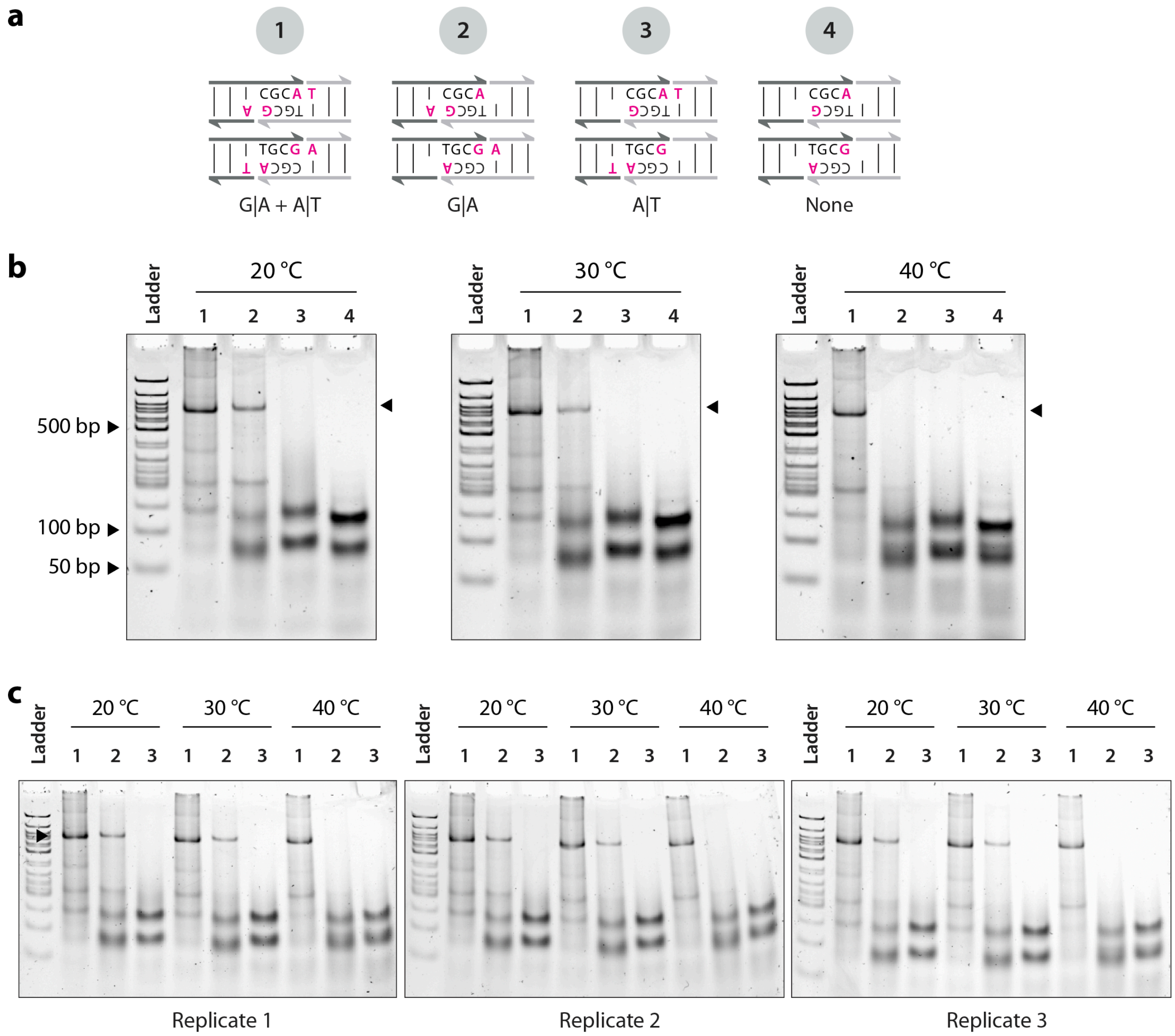

Supplementary Figure 14: **Triplicate gel pics for DNA tetrahedron at various temperatures.** (a) Design and sequences of sticky end interfaces in the tetrahedra. (b) Demonstration of stability of each construct after 1 hour incubation at different temperatures. (c) Full gel images of triplicate experiments in Figure 6d.

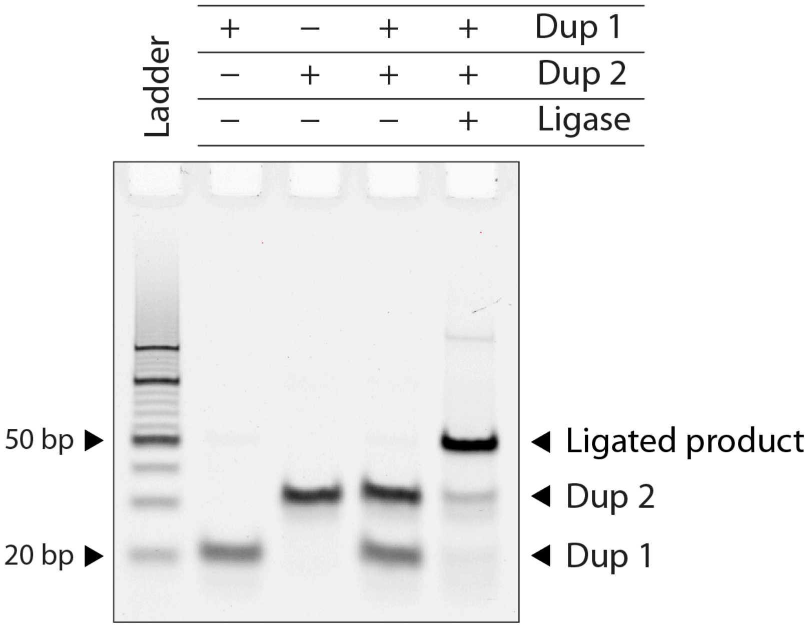

Supplementary Figure 15: **Non-denaturing PAGE confirms the ligation of the two DNA duplexes.** Full image of gel shown in Figure 6g.

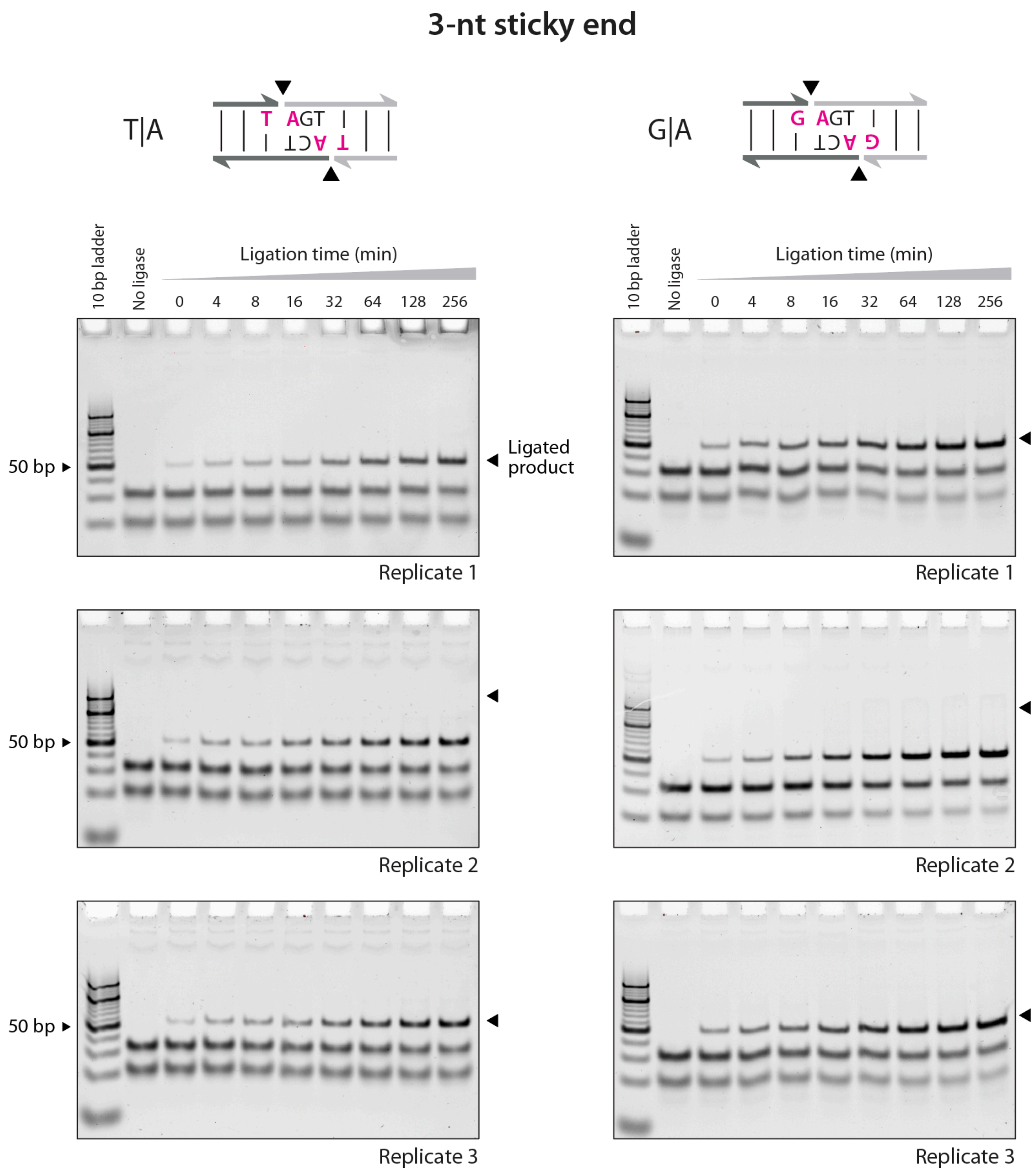

Supplementary Figure 16: Triplicate gel pics for ligation experiments with 3 nt overhang.

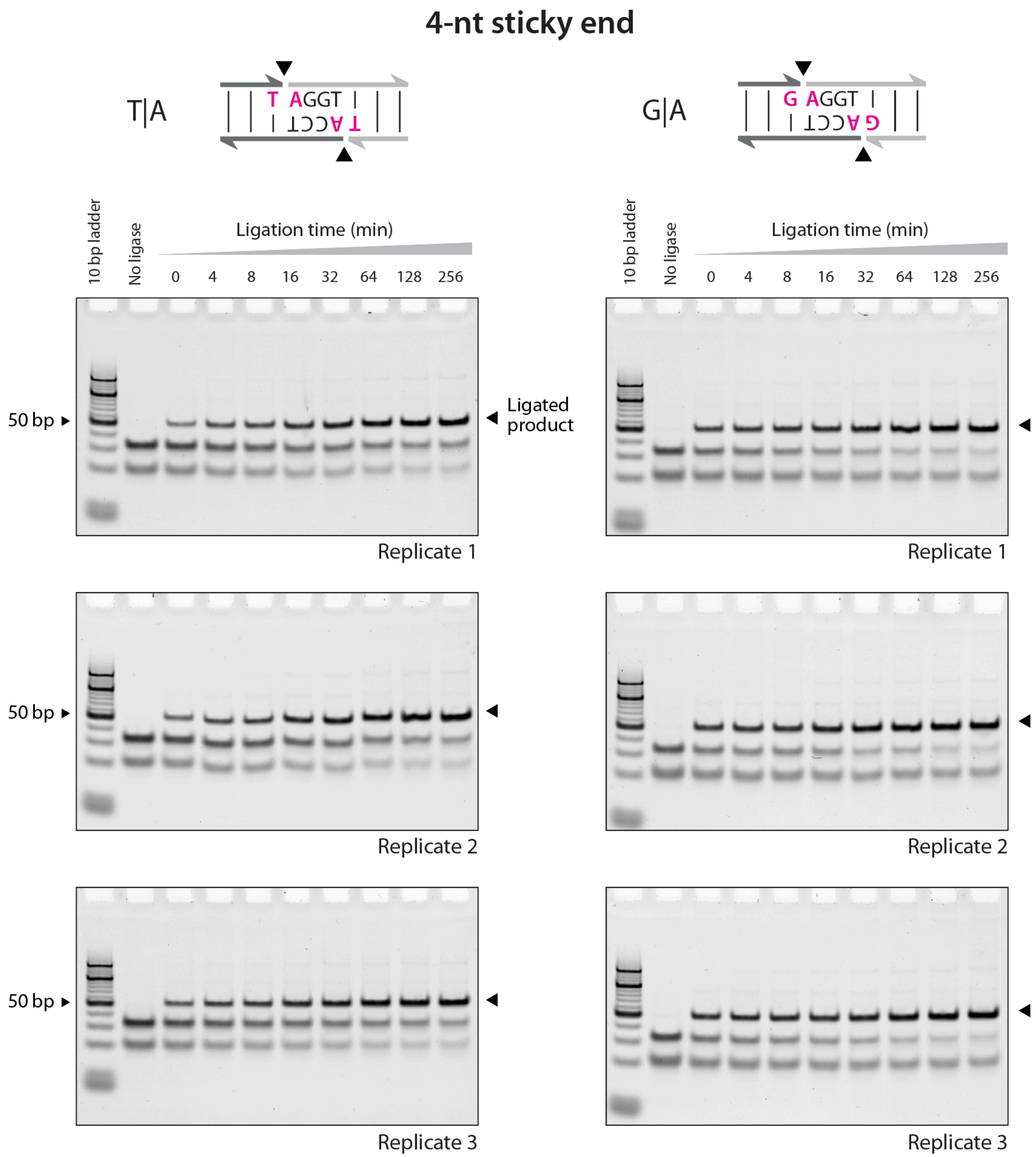

Supplementary Figure 17: Triplicate gel pics for ligation experiments with 4 nt overhang.

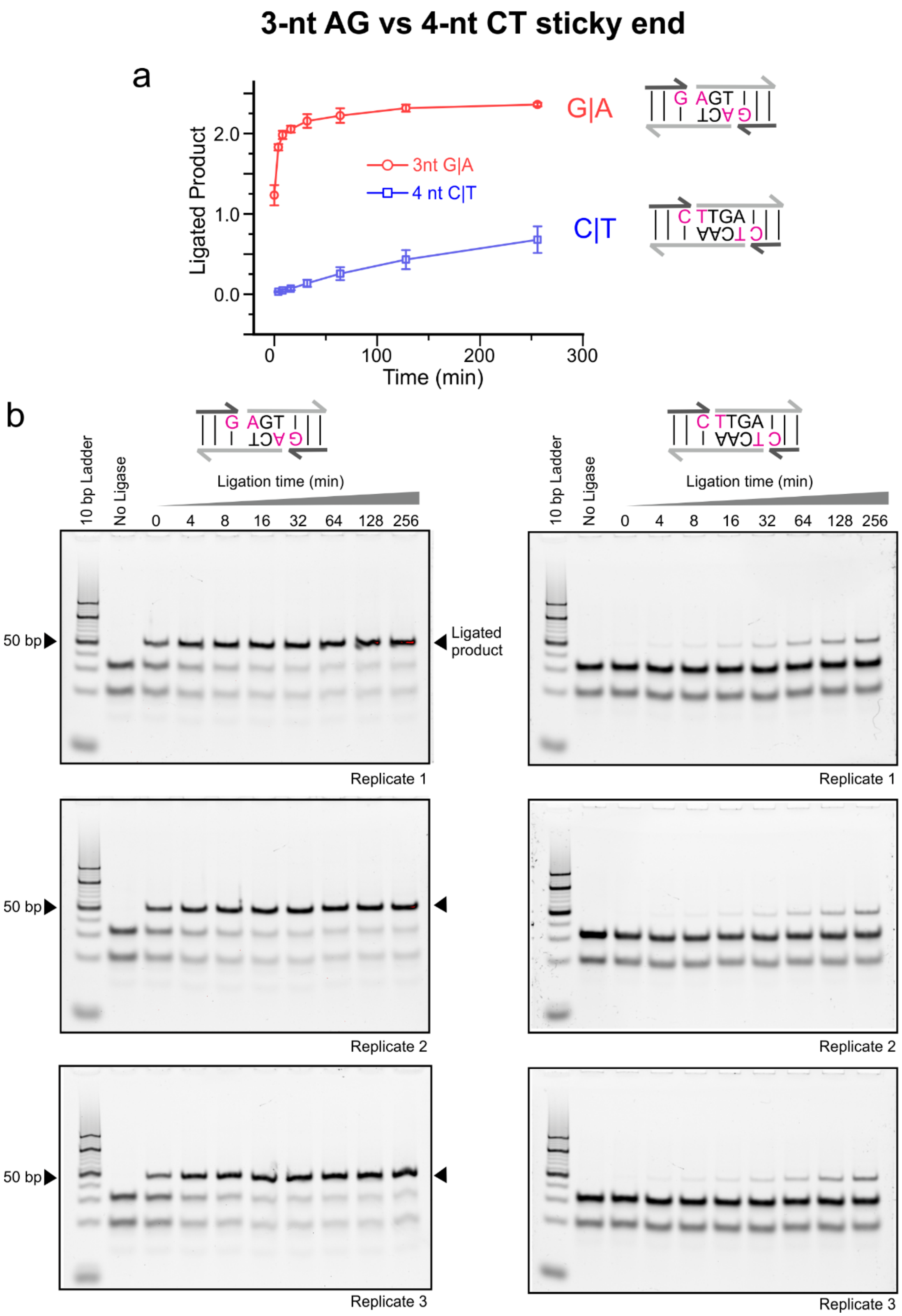

Supplementary Figure S18: Ligation experiments with 3 nt overhang with G|A Stack vs 4 nt overhang with C|T stack. (a) Quantified ligation product of G|A and C|T stacked constructs over time. (b) Triplicate gel pictures.

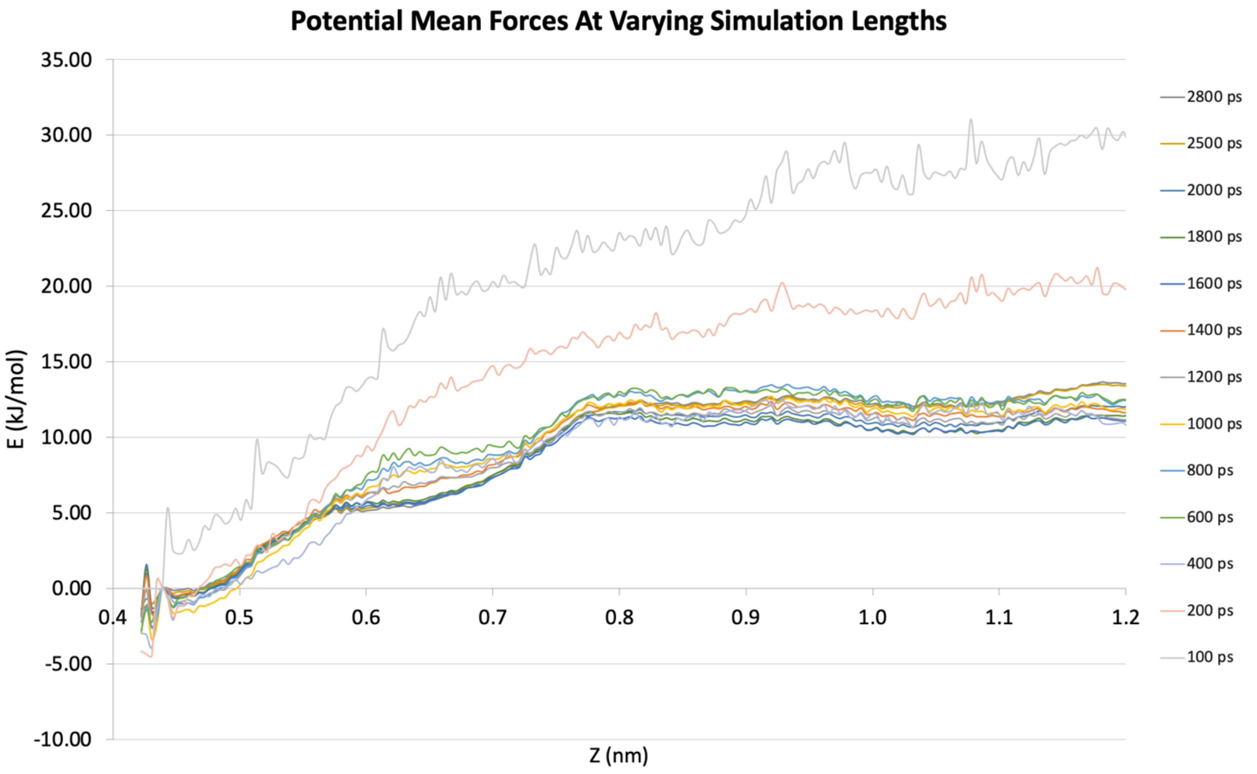

Supplementary Figure S19: Potential mean force at varying simulation lengths. Simulations of AA-TT using bsc1 show convergence of potential mean force with less than 1 ns of simulation time.
