# Supplementary material for: High-throughput single-molecule quantification of individual base stacking energies in nucleic acids
Source: Nat Commun. 2023 Feb 6;14:631. doi: 10.1038/s41467-023-36373-8 (PMC9902561; doi:10.1038/s41467-023-36373-8)
Supplement: Supplementary file 3 — Description of Additional Supplementary Files [file 41467_2023_36373_MOESM3_ESM.docx]

**Description of Additional Supplementary Files**

Supplementary Dataset 1: A zip file containing analysis code with instructions and sample movie images
