# Supplementary material for: High-throughput single-molecule quantification of individual base stacking energies in nucleic acids
Source: Nat Commun. 2023 Feb 6;14:631. doi: 10.1038/s41467-023-36373-8 (PMC9902561; doi:10.1038/s41467-023-36373-8)
Supplement: Supplementary file 4 — Supplementary Software [file 41467_2023_36373_MOESM4_ESM.zip › analysis software and sample data/User Guide for CFM -Matlab analysis program.docx]

**Manual for the MATLAB program for bead dissociation analysis**

System requirements: A windows-based PC running Matlab. Tested for Matlab 2021 on a Windows 10 OS.

This program will help to create time trace of tethered beads observed in the single-molecule experiments with the CFM. The program has 4 sections

**Section 1** – Load images & timestamp file from the folder

**Section 2** – This involves identification of beads and bead selection

**Section 3** – Creation of time trace of tethered beads

**Section 4** – Calculation of bead dissociation time

Each section will be described below

**Section 1 –**


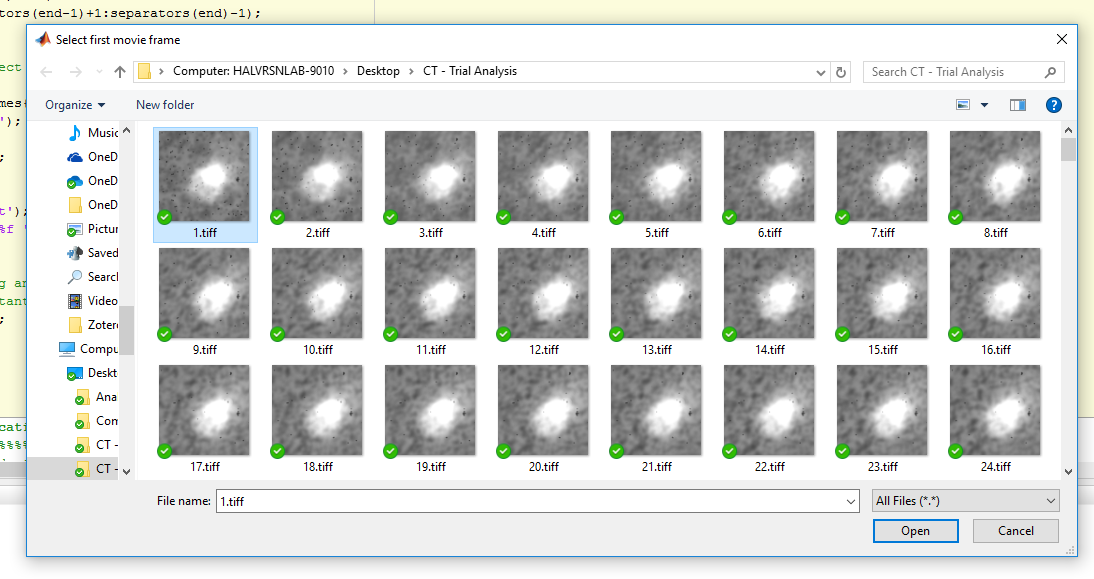
**1.1** Running this section will prompt you to choose the folder in which the data is stored. Open the folder and click on any image file and choose open

1.2 After selecting the folder, program will ask you to input the start frame and end frame sequentially in the command box. Start frame would be the first frame after centrifuge assumes full speed, indicated by stabilized image (meaning no lateral change in region of Interest) . You have to manually identify the start frame by going through the images. End frame is typically the last frame in the folder.


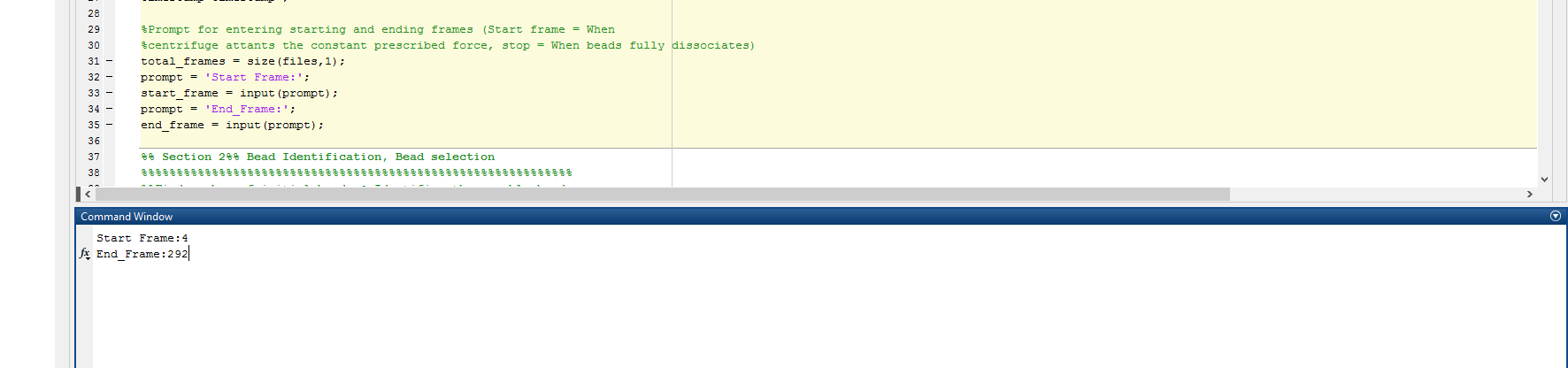


**Section 2** – This section identifies beads and using a combination of algorithm and manual inputs beads good for analysis are chosen.

**2.1 –** Using the inbuilt imfindcircles function on the first frame, beads will be identified. Here we have to choose the correct sensitivity (0 to 1) to identify all possible beads. The program will show the results of 3 sensitivity options (0.8, 0.85 and 0.9).


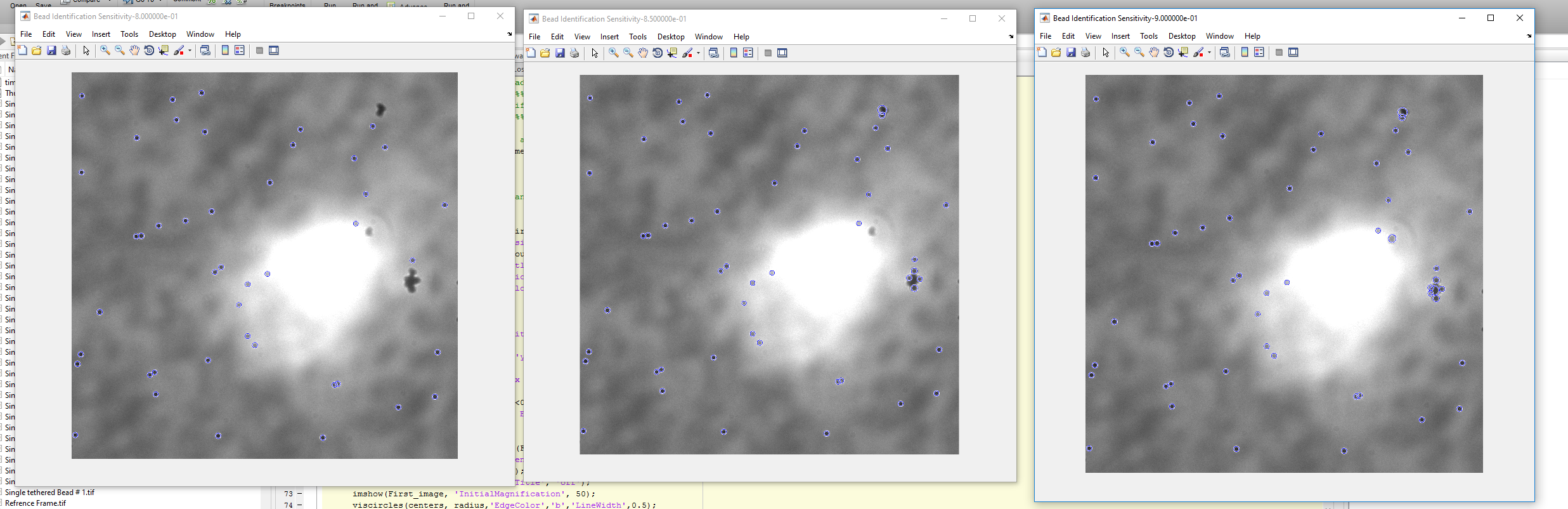


Typically, 0.8 will be a good choice.
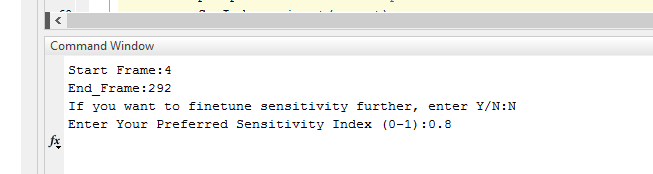


But there is option to finetune sensitivity if selection is poor. If you want to finetune When prompted, select Y and input the sensitivity (0-1). You can continue to do so until all beads are selected.

**2.2**


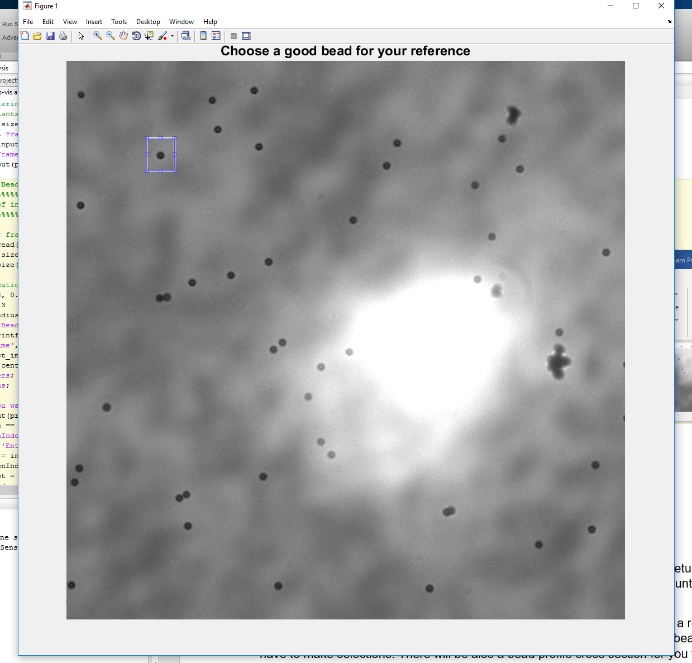
Once the preferred sensitivity is entered, program will request you to identify a reference bead. This bead will act as a visual aid you you and will be displayed next to other beads when you have to make selections. There will be also a bead profile cross section for you to compare. To choose the reference bead, draw an approximate square around the bead and right-click on it and crop image.


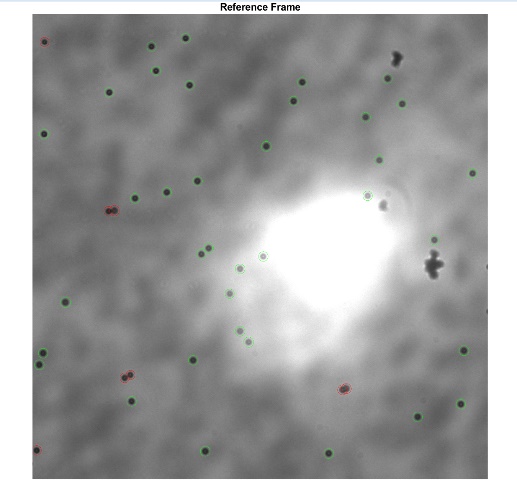

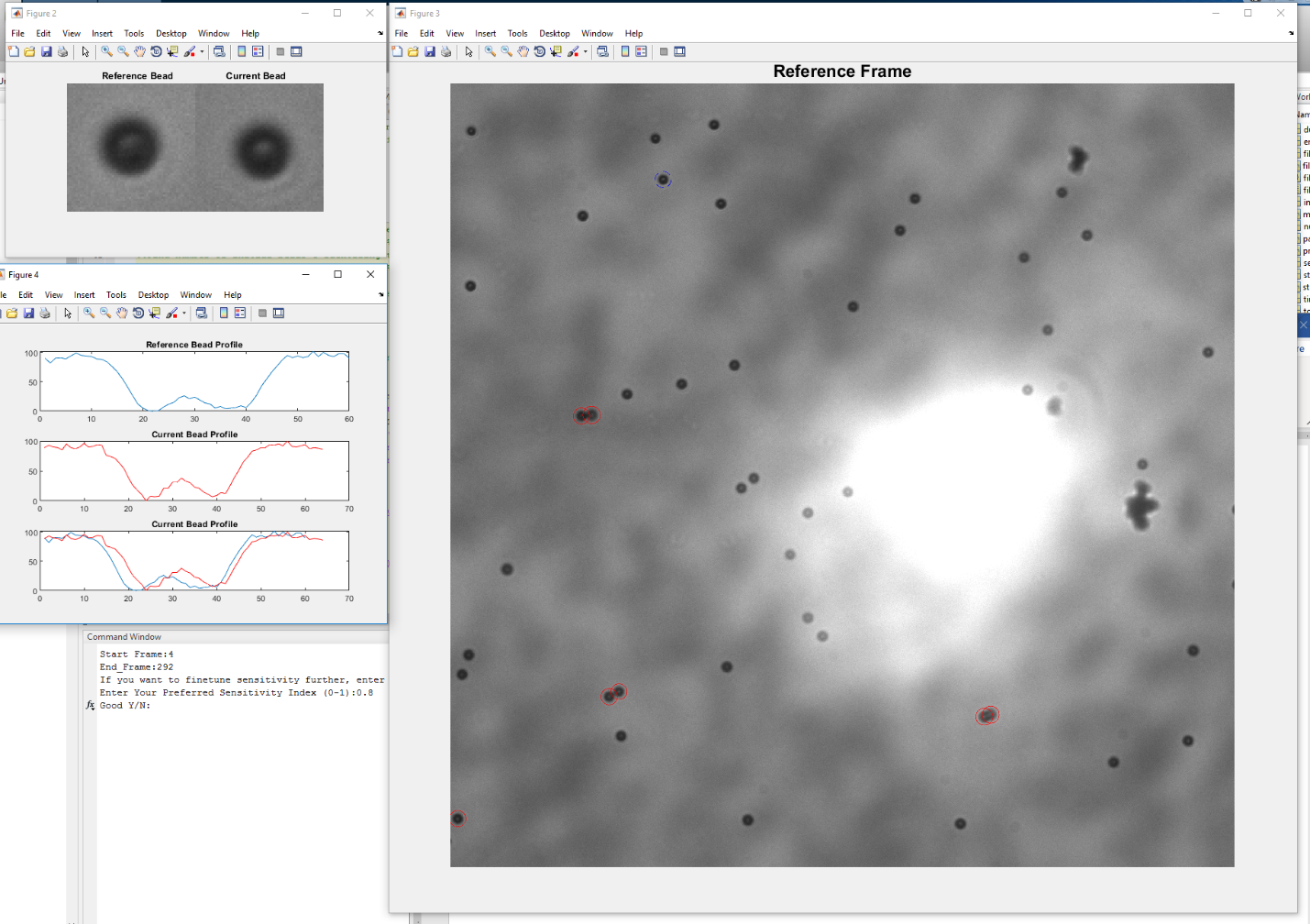
Once reference beads are chosen, program will go through all the beads and check for few conditions. All beads that are in cluster or very close proximity will be marked as bad beads. (See below in the reference frame). Beads that are very close to the edge and also marked bad and not considered for analysis. After automatically excluding these beads, you have to manually ensure the remaining beads are fit for analysis. To help this purpose, the program will walk you though each bead (current bead of question will be circled in blue), display it next to the reference bead you chose in earlier steps and will also show the cross-section profile of both the beads (see the left side of the image). Beads of un-usual size, dirt cladding beads, beads that are out of focus (most likely stuck to the surface) should be excluded by entering “N” or “n” in the command window. For good beads enter “Y” or “y”

Once all bead selections are done, this frame will be saved in the folder along with the images for future records. All the good beads selected for analysis will be marked with green circle and all rejected beads will be marked in red circles

**Section 3** – This section will help tracking the beads in time. A square area around the bead is cropped from the frames and stored in a matrix. The images of the same beads from successive slides will be placed in series left to right to aid the observation. From these images you can easily identify the frame at which dissociation happens.

As you run this section, program will display the average time difference between the slides (experimental time resolution in seconds) See below. You have to enter your preferred analysis time resolution in multiples of experimental time. (Typically, 30 sec for these experiments).


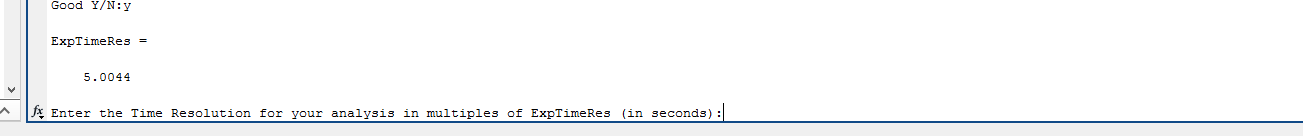


Once the experiment time is entered, program will go through every n^th^ (n=Analysis time resolution/exp time resolution). A square area around the bead is cropped from the frames and stored in a matrix. The images of the same beads from successive slides will be placed in series left to right to form bead-time trace. See below


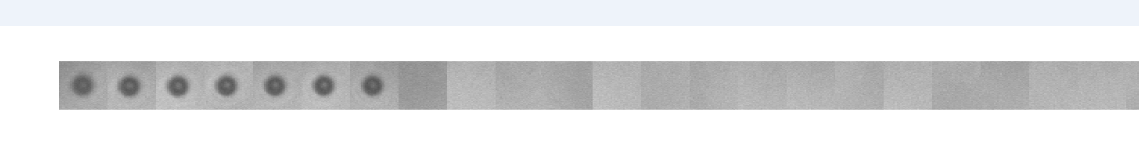


This indicates that bead dissociated after the 7^th^ frame. From the timestamp associated with each image, we can calculate the time of dissociation of the bead. These images will be also automatically saved in the same folder as data.

To visually inspect large number of beads, in addition to the single bead trace, a image with every 20 beads is also created and stored. See below


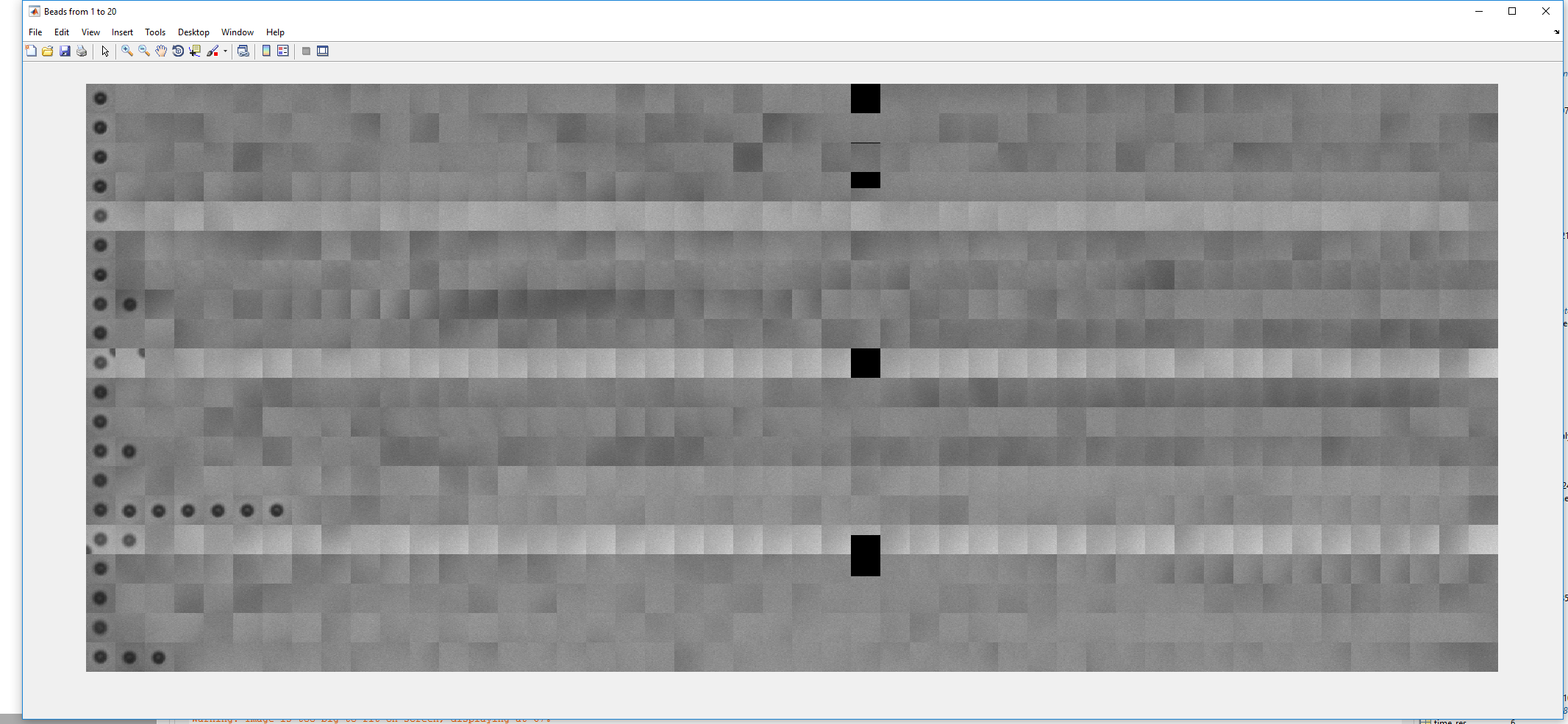


(The black lines are due to pixels lost in wireless transmission of the images and can occur rarely in experiments).

**Section 4** - Calculation of binned dissociation time

When this section is run, the program calculates the binned tether dissociation time based on the analysis time resolution chosen. In order to do this variance around each bead is calculated. The program will look for sharp changes in variance (typically >3 times the mean variance). Sharp variance indicates dead dissociation. Program will calculate the bead dissociation time based on the location of this sharp transition from the timestamp associated with each image. However, error can occur due to various circumstances including presence of null pixels, motion of other beads in vicinity etc. The dissociation time will be stored in the variable “beaddisstime”. The beads that don’t dissociate will be given a value “100000” and presence of null pixel will be given “100001” as program cannot reliably calculate dissociation time for these cases. These beads should be evaluated manually from the bead-time trace generated in section 3 and values should be entered for further analysis. All the values obtained from the program should be manually verified from the bead-time trace generated in section 3. The workspace is saved which will contain all the variable generated for the analysis.
