# Supplementary figures and images for: High-throughput single-molecule quantification of individual base stacking energies in nucleic acids
Source: Nat Commun. 2023 Feb 6;14:631. doi: 10.1038/s41467-023-36373-8 (PMC9902561; doi:10.1038/s41467-023-36373-8)

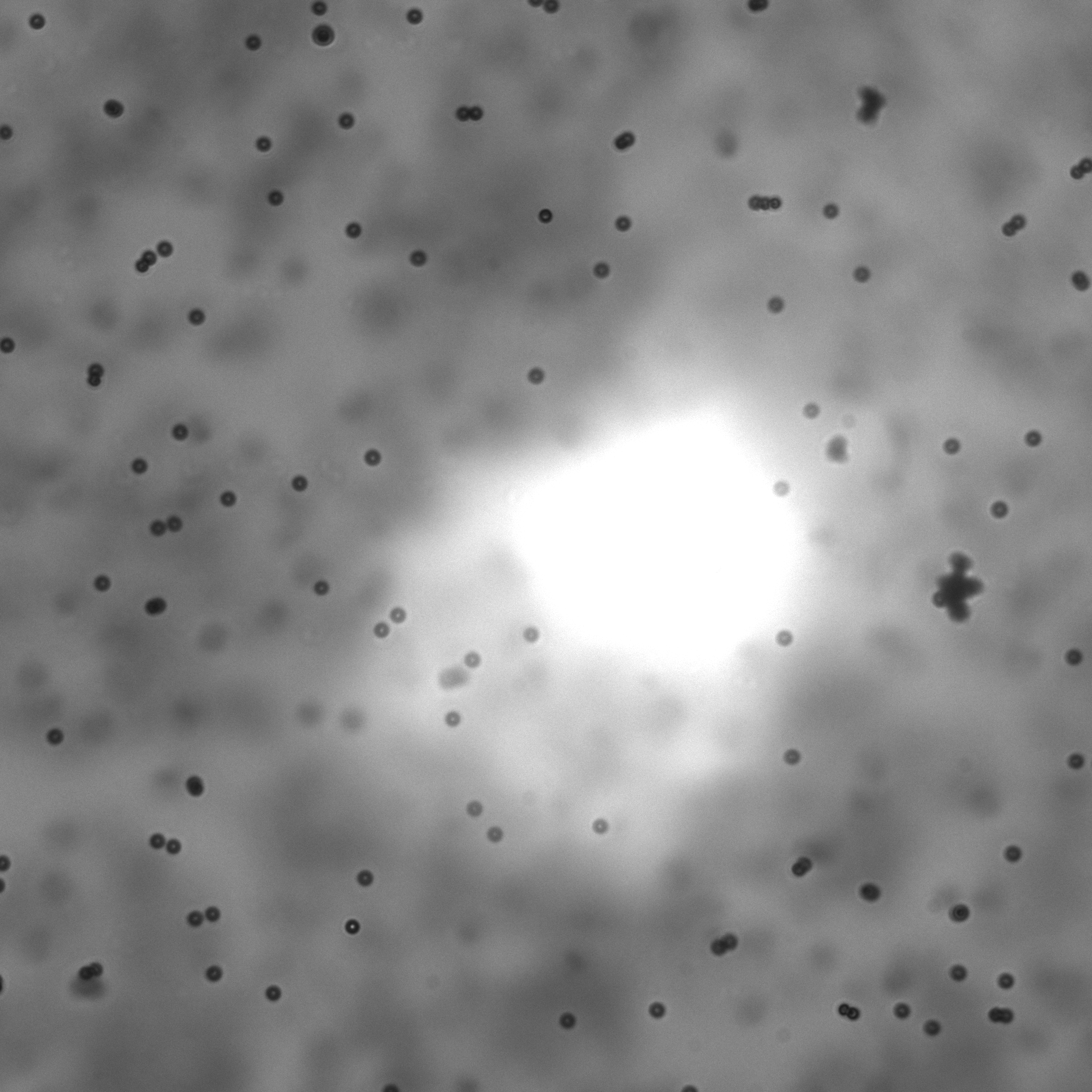

Supplement: Supplementary file 4 — Supplementary Software [file 41467_2023_36373_MOESM4_ESM.zip › analysis software and sample data/CT - Trial Analysis - Sample/1.tiff]

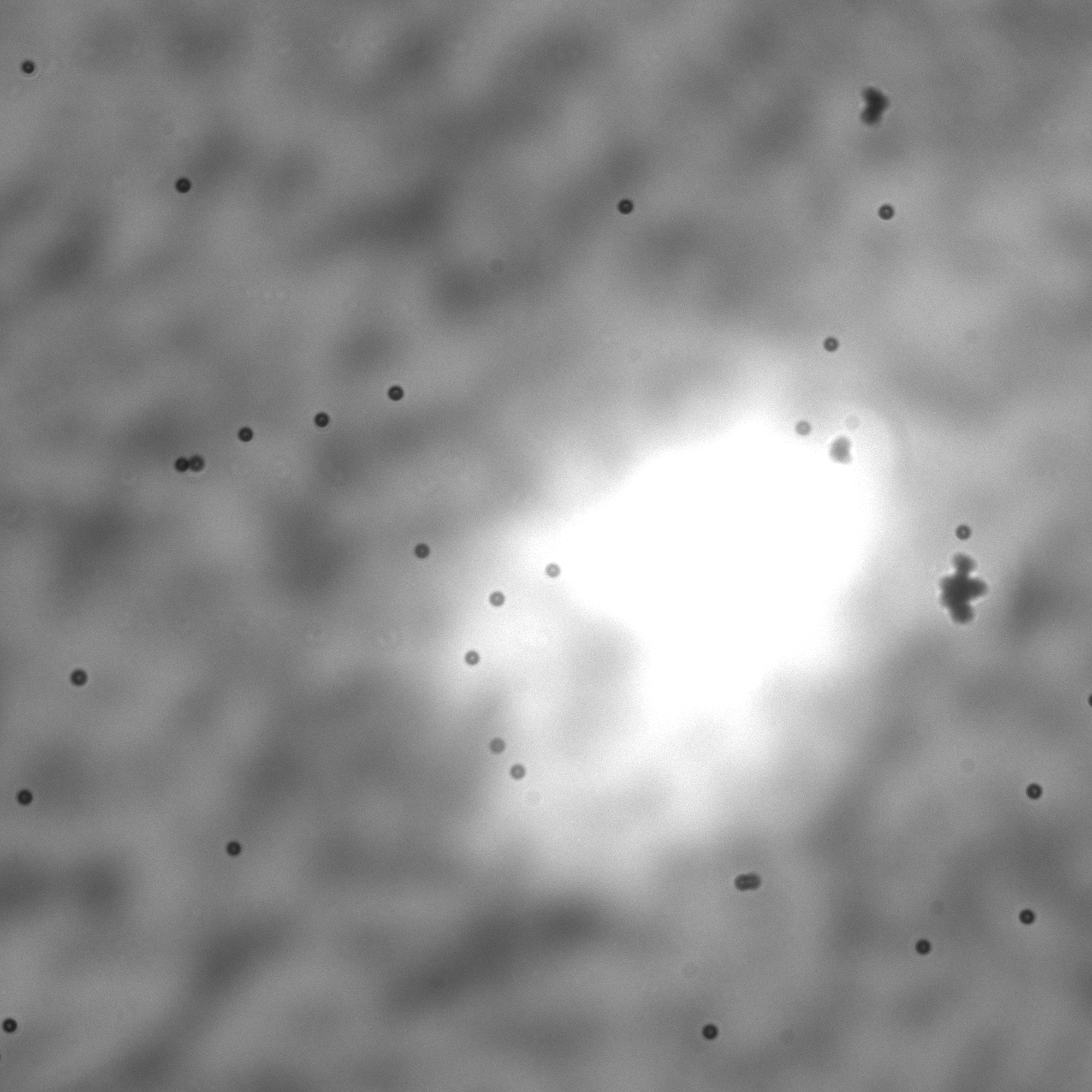

Supplement: Supplementary file 4 — Supplementary Software [file 41467_2023_36373_MOESM4_ESM.zip › analysis software and sample data/CT - Trial Analysis - Sample/10.tiff]

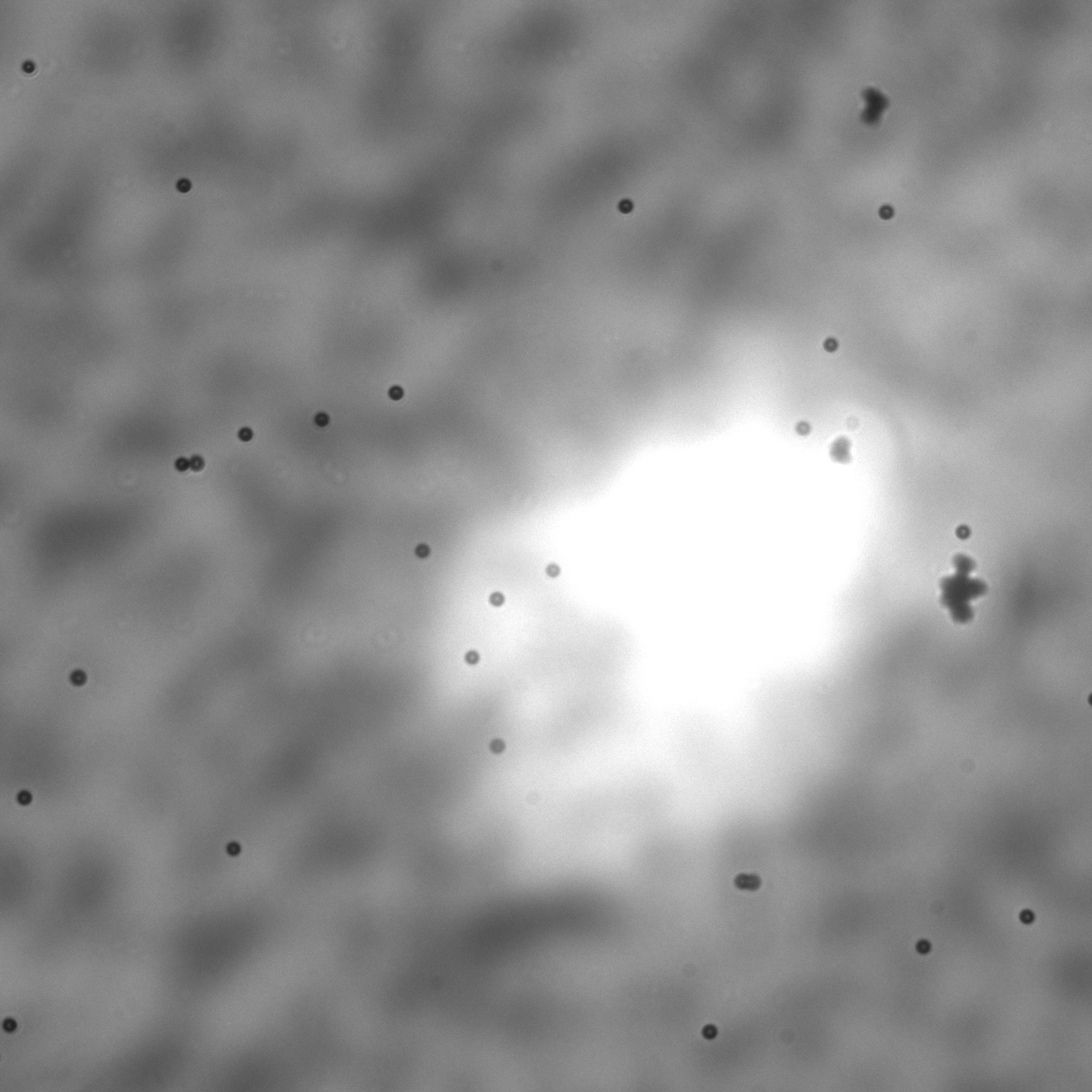

Supplement: Supplementary file 4 — Supplementary Software [file 41467_2023_36373_MOESM4_ESM.zip › analysis software and sample data/CT - Trial Analysis - Sample/11.tiff]

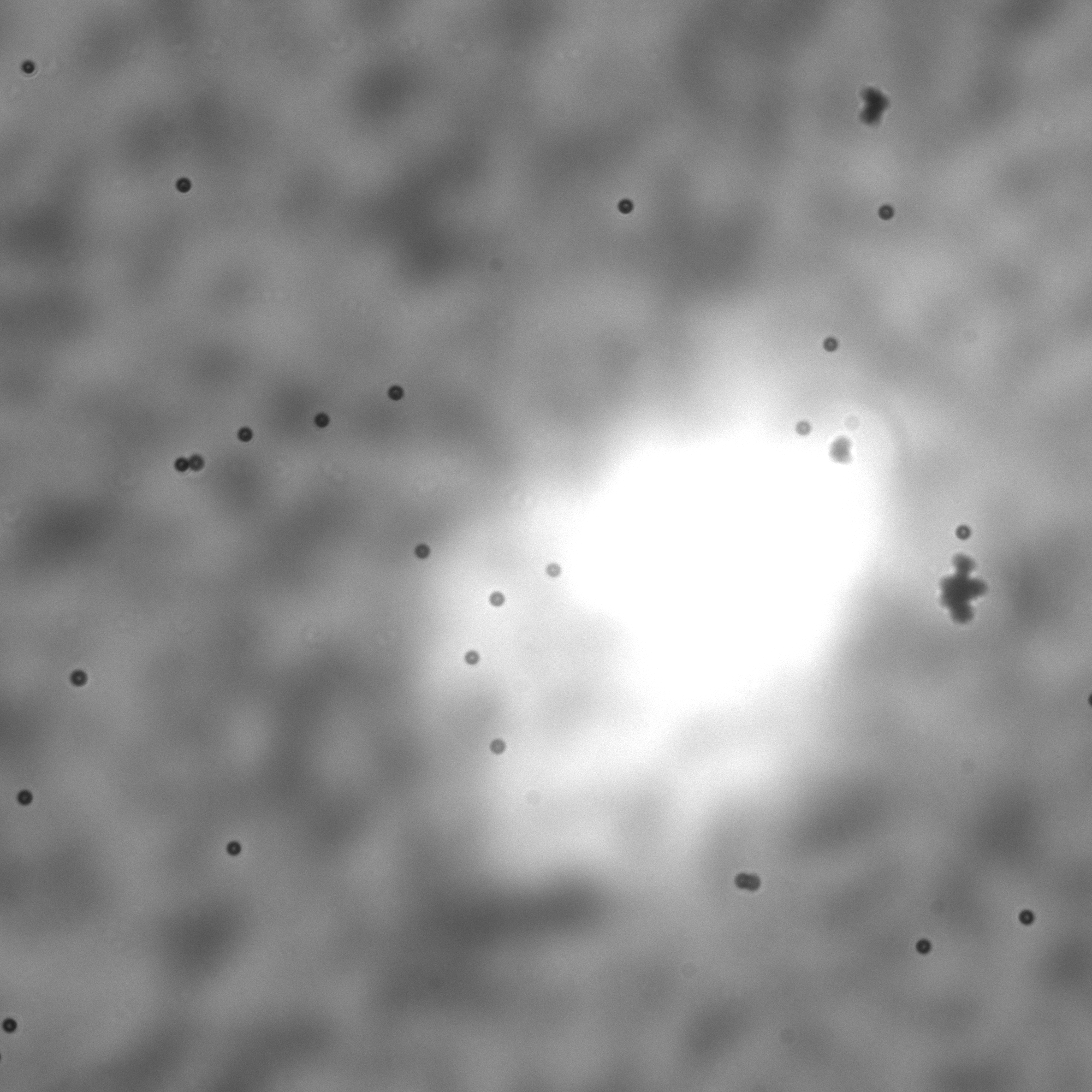

Supplement: Supplementary file 4 — Supplementary Software [file 41467_2023_36373_MOESM4_ESM.zip › analysis software and sample data/CT - Trial Analysis - Sample/12.tiff]

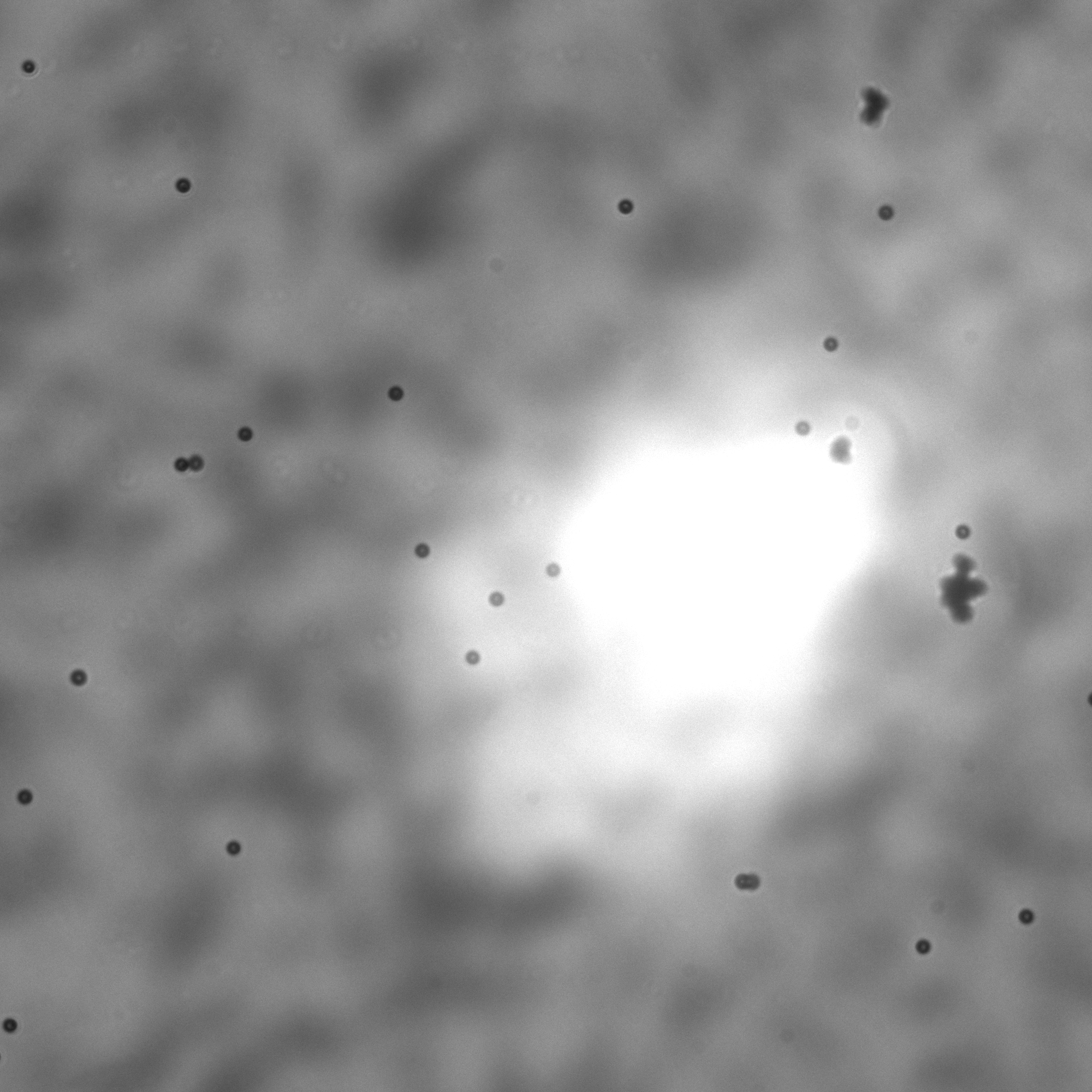

Supplement: Supplementary file 4 — Supplementary Software [file 41467_2023_36373_MOESM4_ESM.zip › analysis software and sample data/CT - Trial Analysis - Sample/13.tiff]

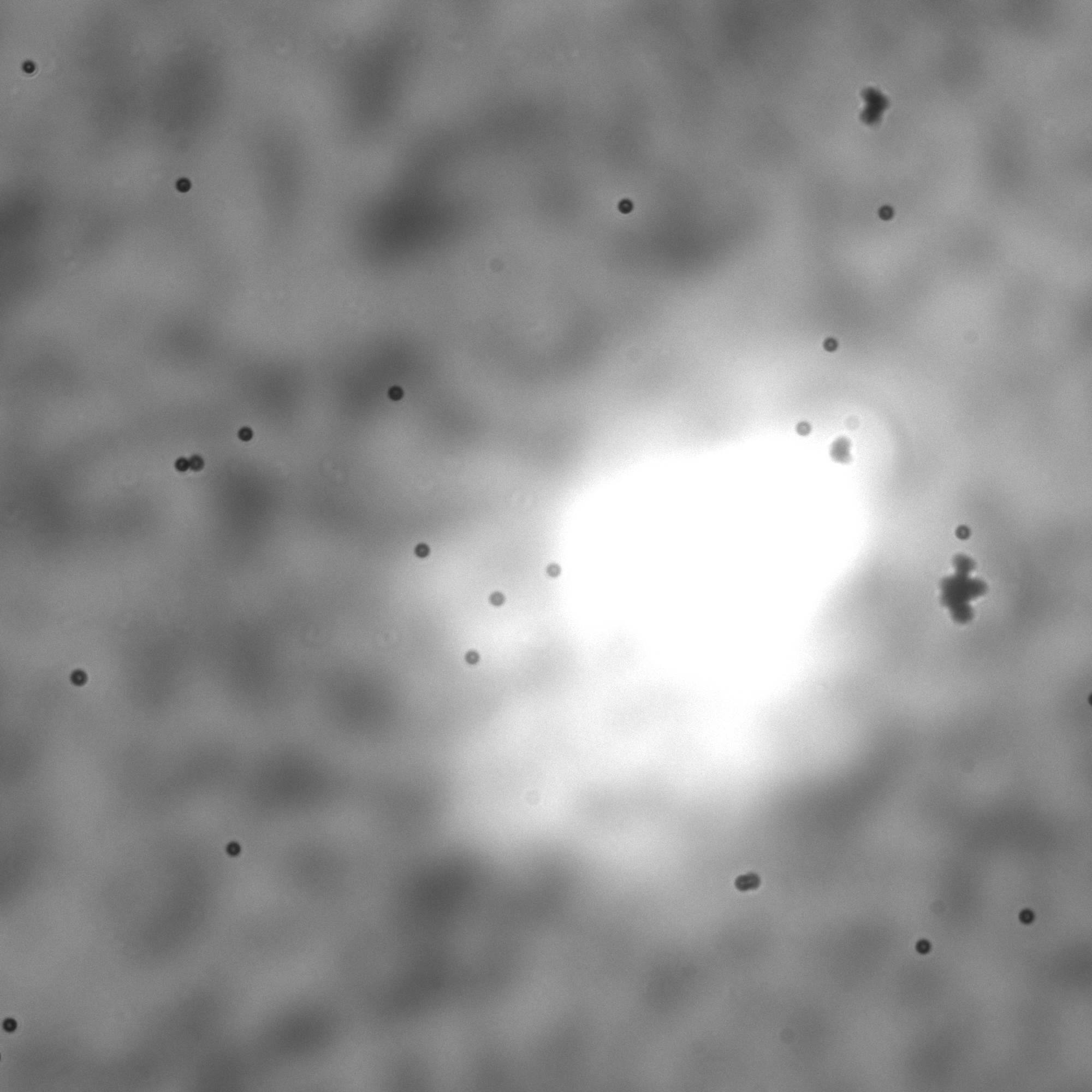

Supplement: Supplementary file 4 — Supplementary Software [file 41467_2023_36373_MOESM4_ESM.zip › analysis software and sample data/CT - Trial Analysis - Sample/14.tiff]

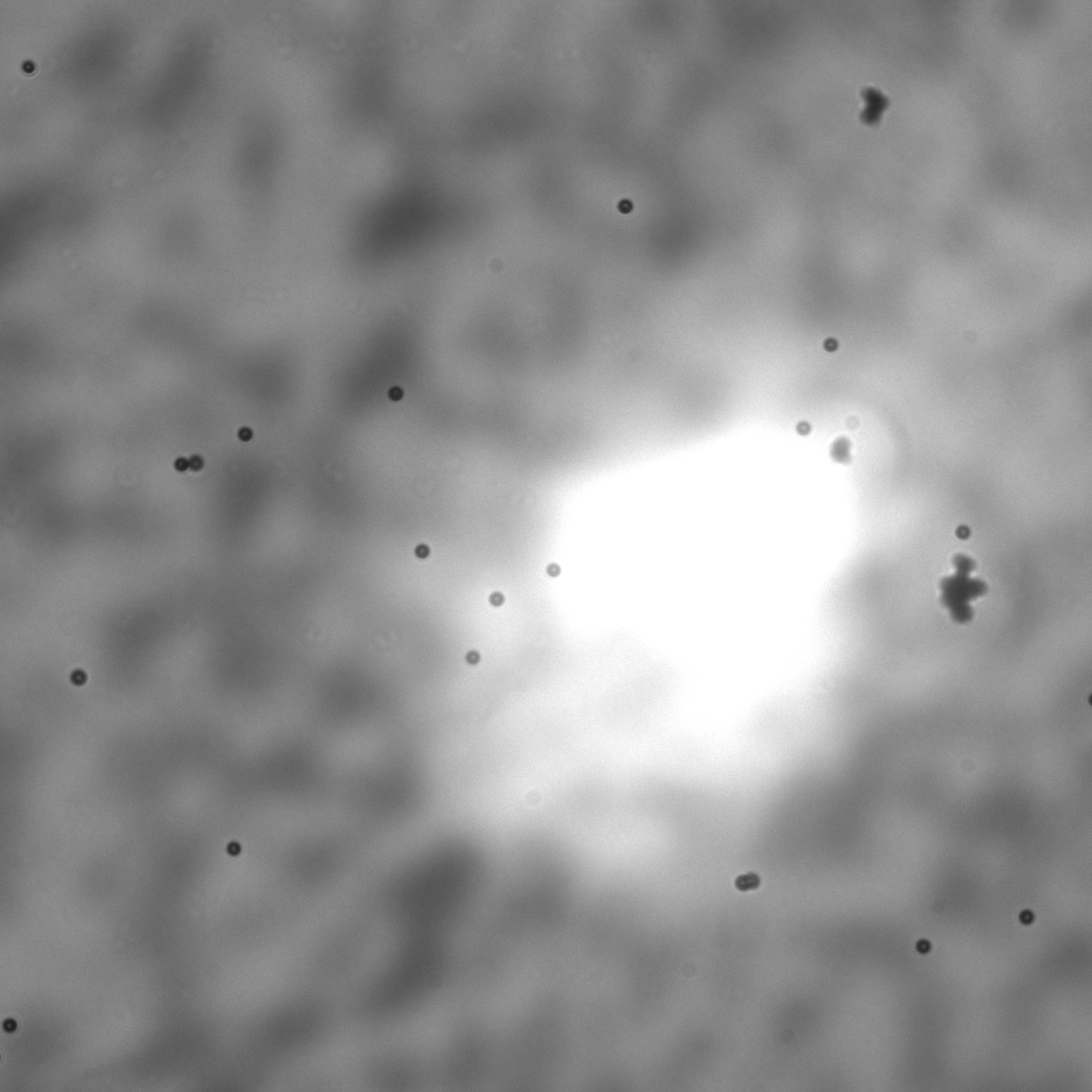

Supplement: Supplementary file 4 — Supplementary Software [file 41467_2023_36373_MOESM4_ESM.zip › analysis software and sample data/CT - Trial Analysis - Sample/15.tiff]

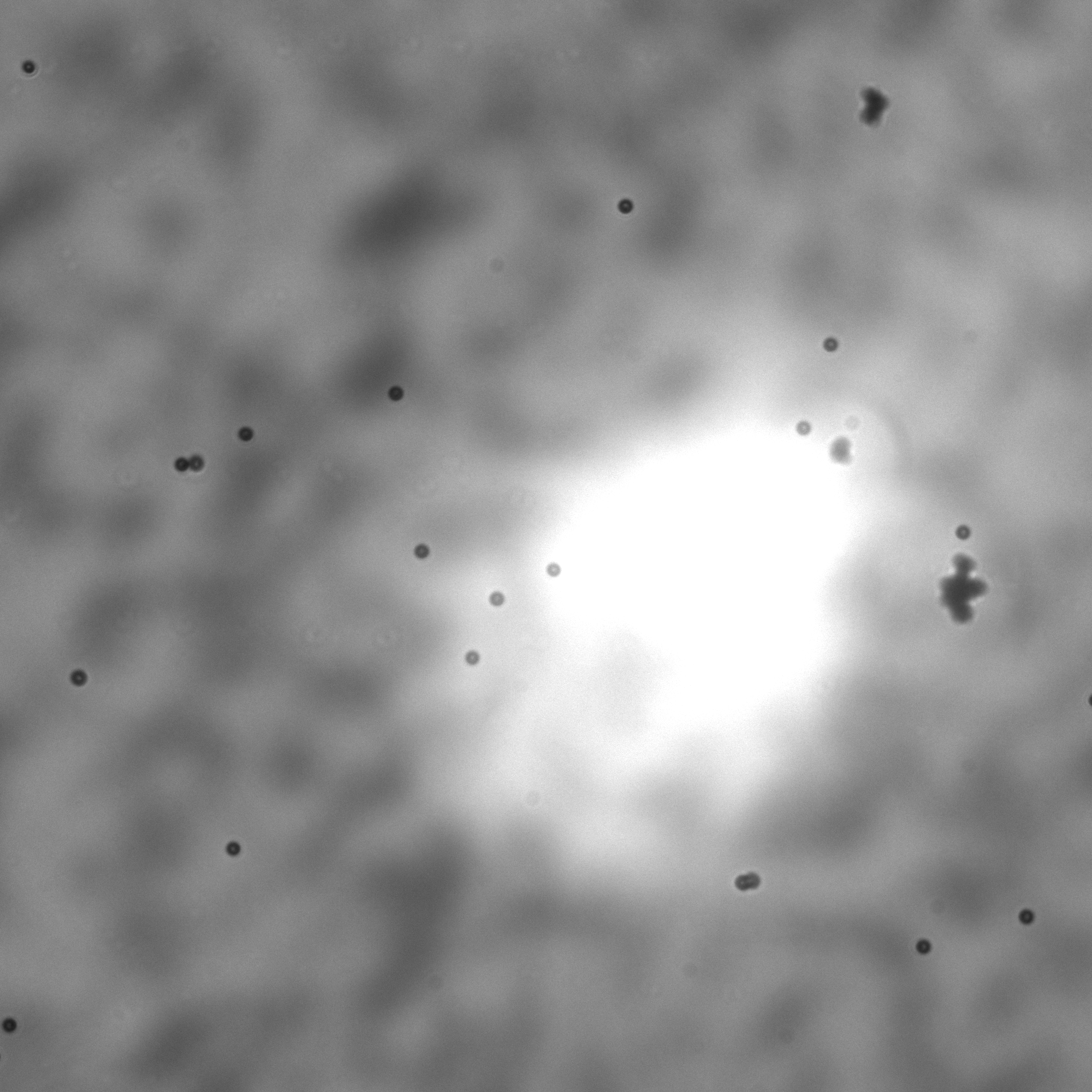

Supplement: Supplementary file 4 — Supplementary Software [file 41467_2023_36373_MOESM4_ESM.zip › analysis software and sample data/CT - Trial Analysis - Sample/16.tiff]

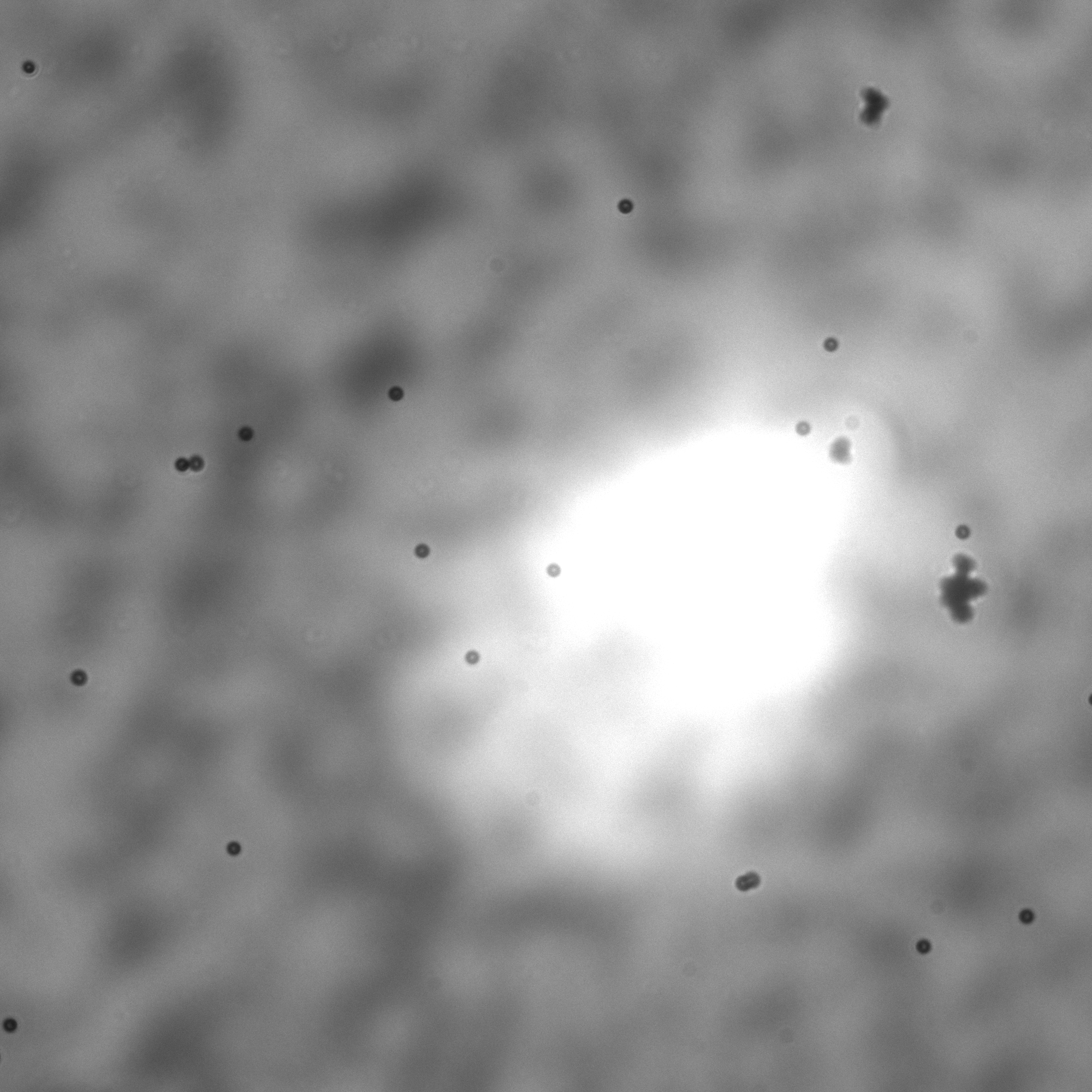

Supplement: Supplementary file 4 — Supplementary Software [file 41467_2023_36373_MOESM4_ESM.zip › analysis software and sample data/CT - Trial Analysis - Sample/17.tiff]

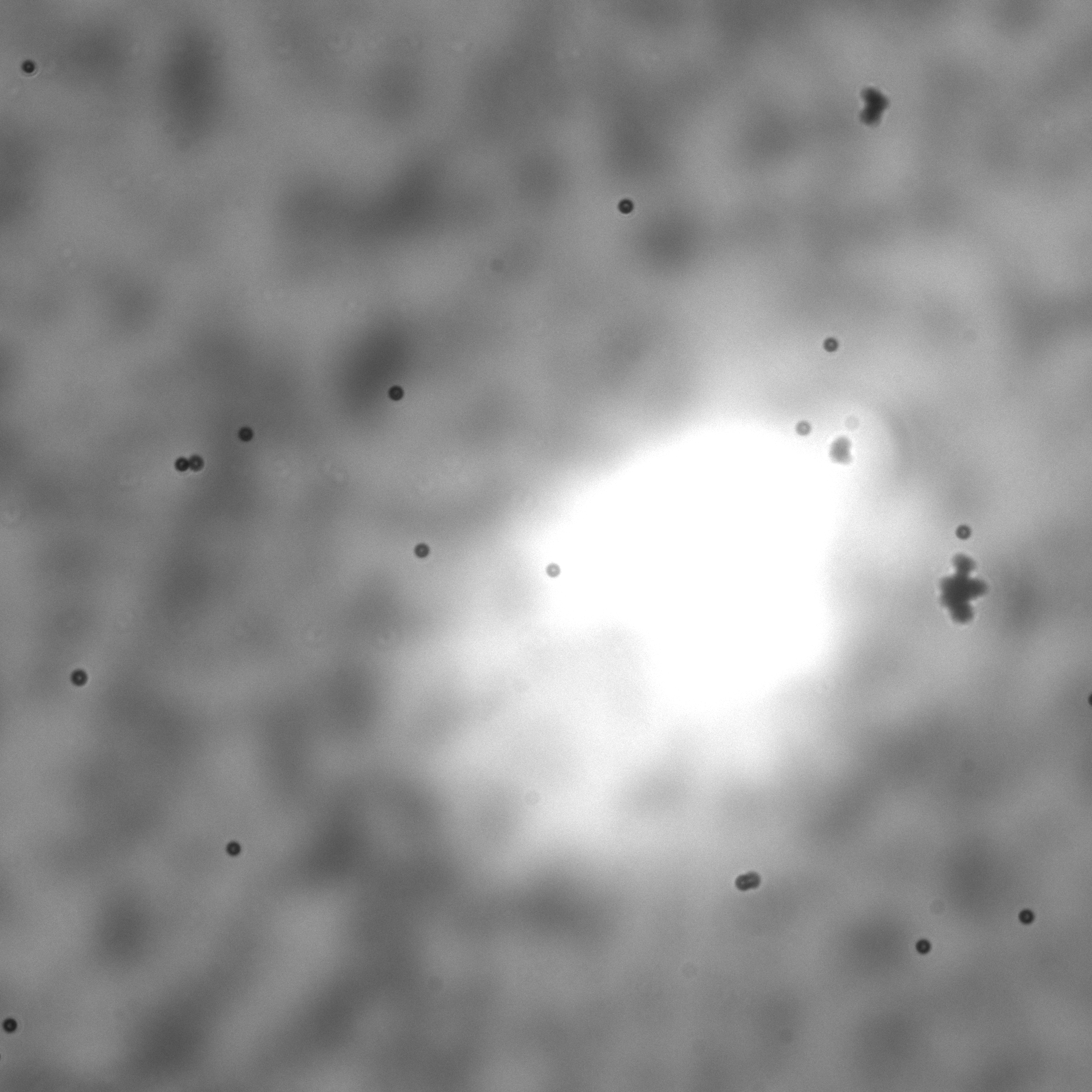

Supplement: Supplementary file 4 — Supplementary Software [file 41467_2023_36373_MOESM4_ESM.zip › analysis software and sample data/CT - Trial Analysis - Sample/18.tiff]

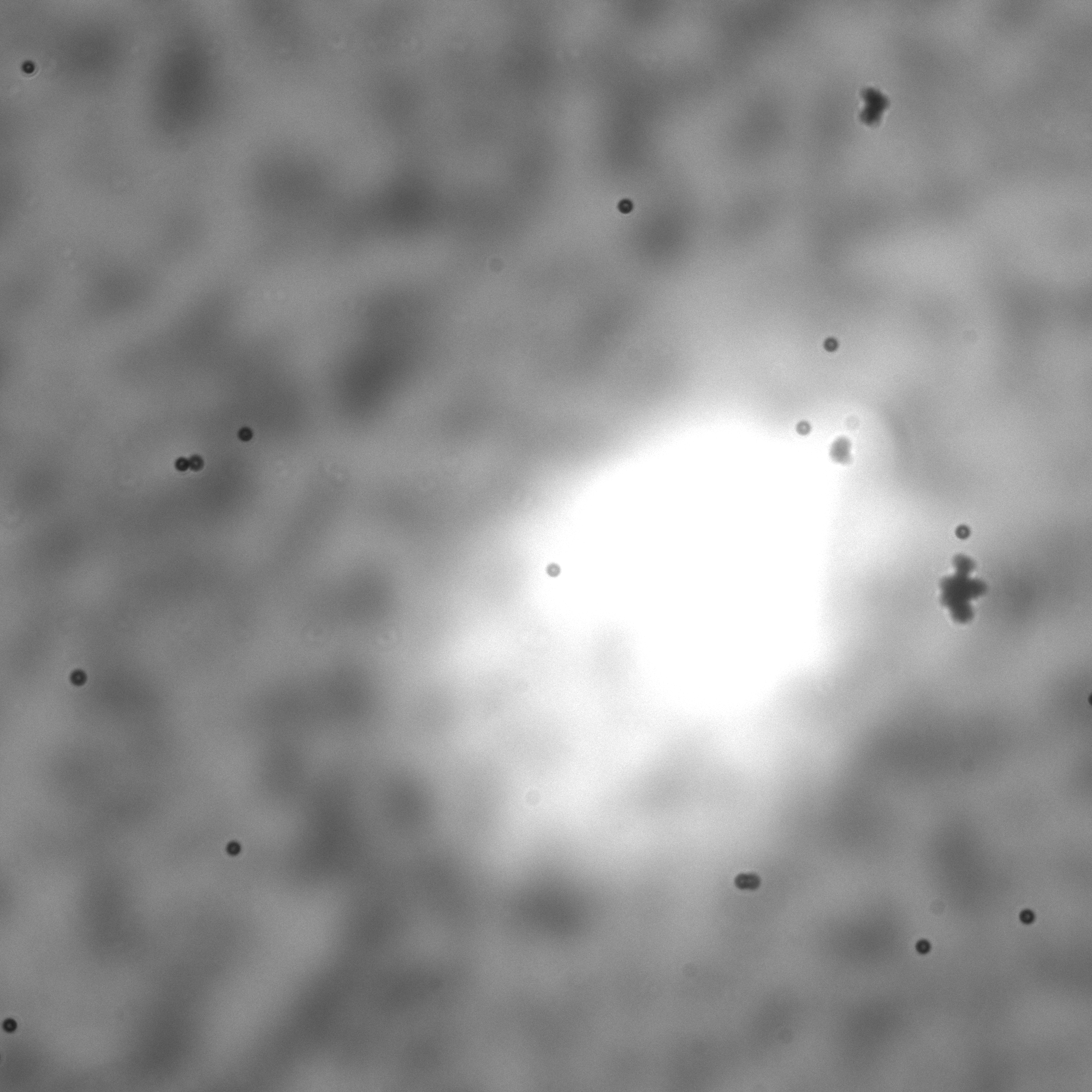

Supplement: Supplementary file 4 — Supplementary Software [file 41467_2023_36373_MOESM4_ESM.zip › analysis software and sample data/CT - Trial Analysis - Sample/19.tiff]

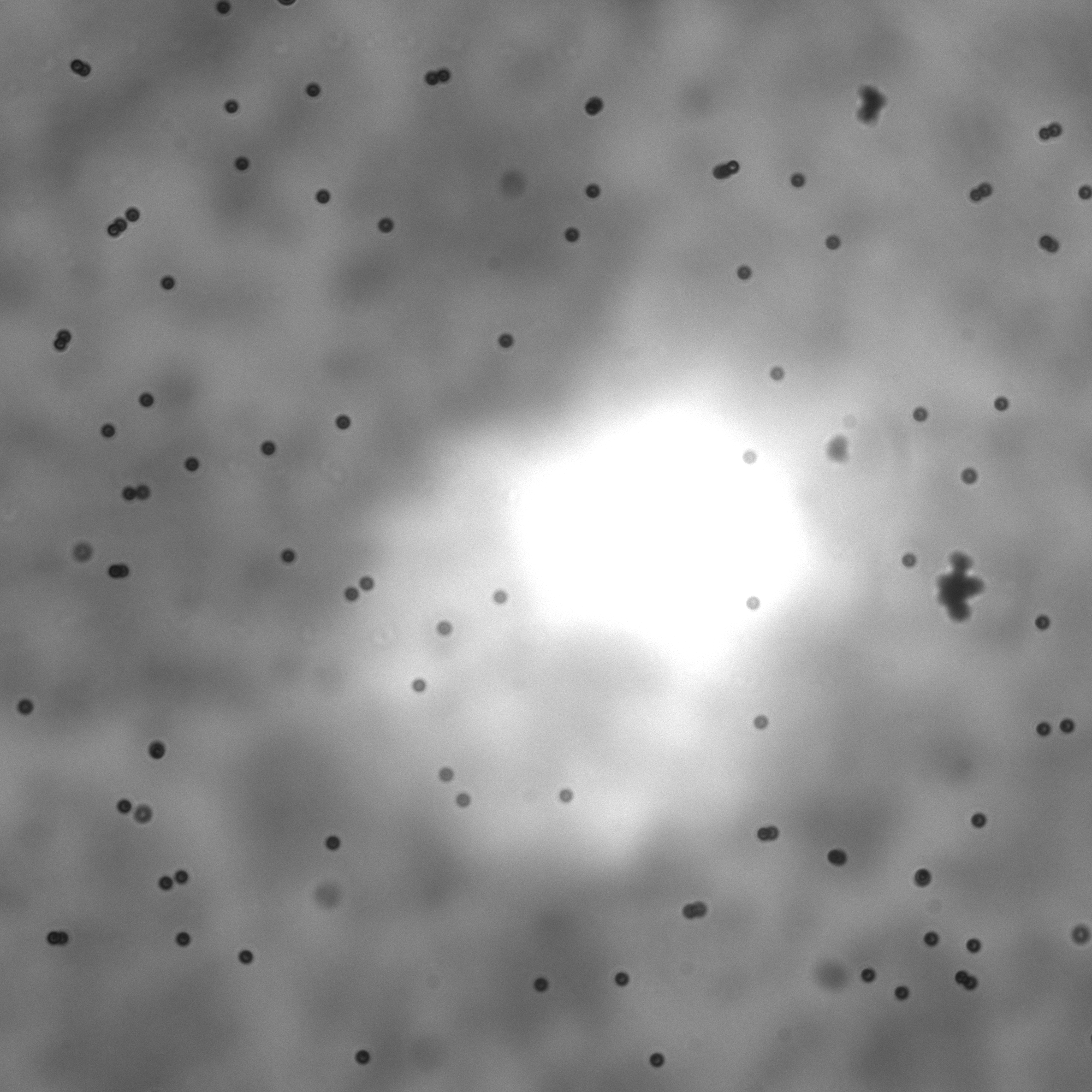

Supplement: Supplementary file 4 — Supplementary Software [file 41467_2023_36373_MOESM4_ESM.zip › analysis software and sample data/CT - Trial Analysis - Sample/2.tiff]

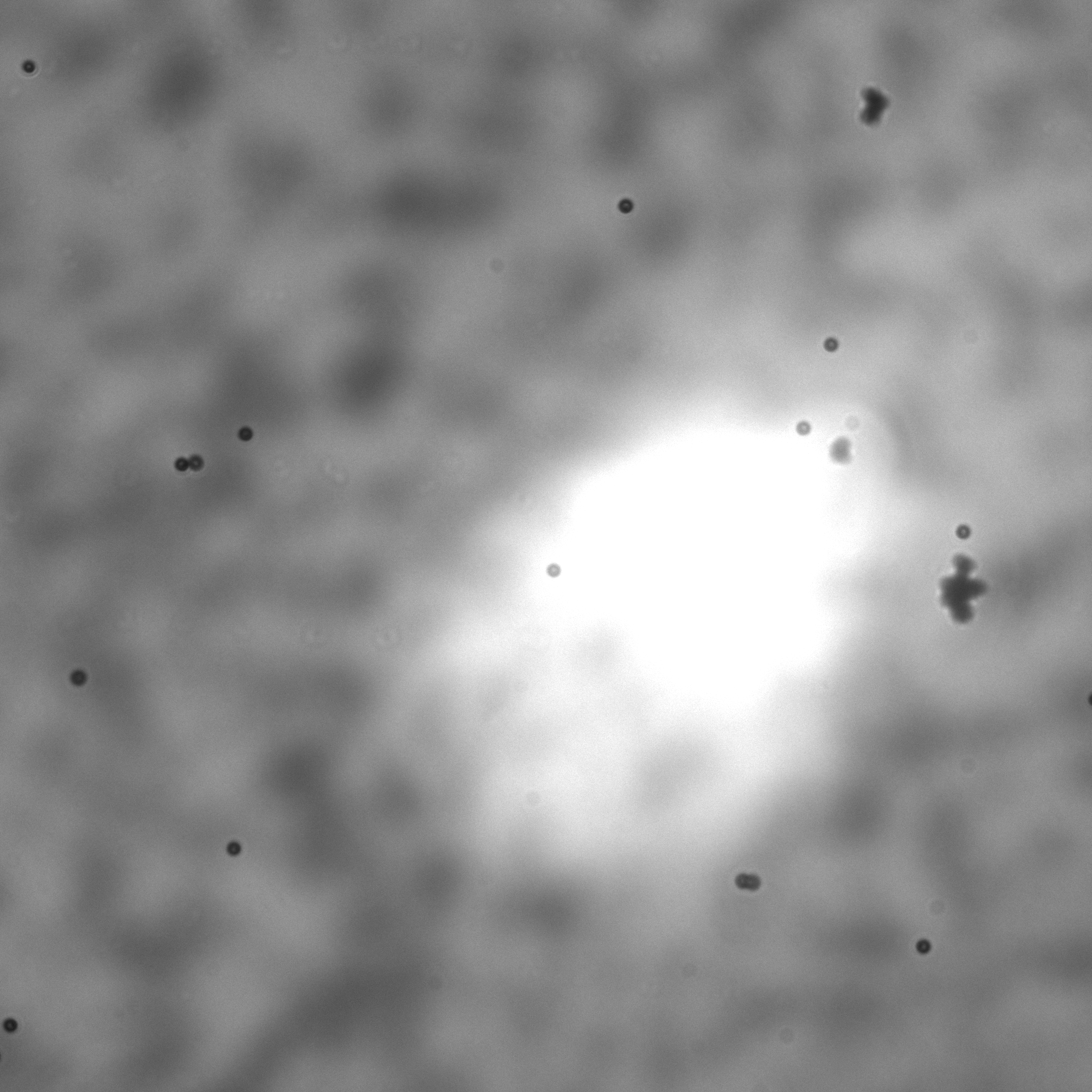

Supplement: Supplementary file 4 — Supplementary Software [file 41467_2023_36373_MOESM4_ESM.zip › analysis software and sample data/CT - Trial Analysis - Sample/20.tiff]

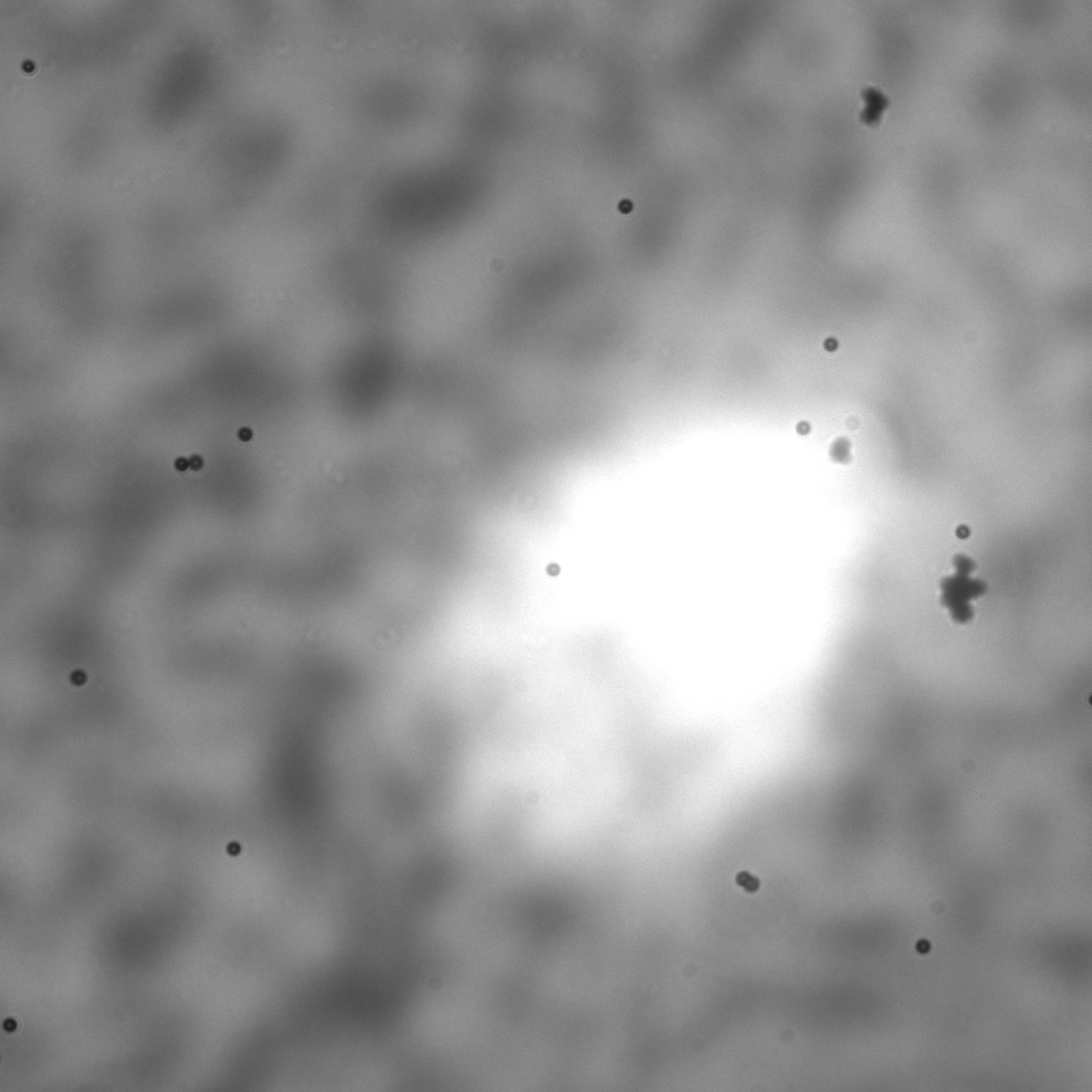

Supplement: Supplementary file 4 — Supplementary Software [file 41467_2023_36373_MOESM4_ESM.zip › analysis software and sample data/CT - Trial Analysis - Sample/21.tiff]

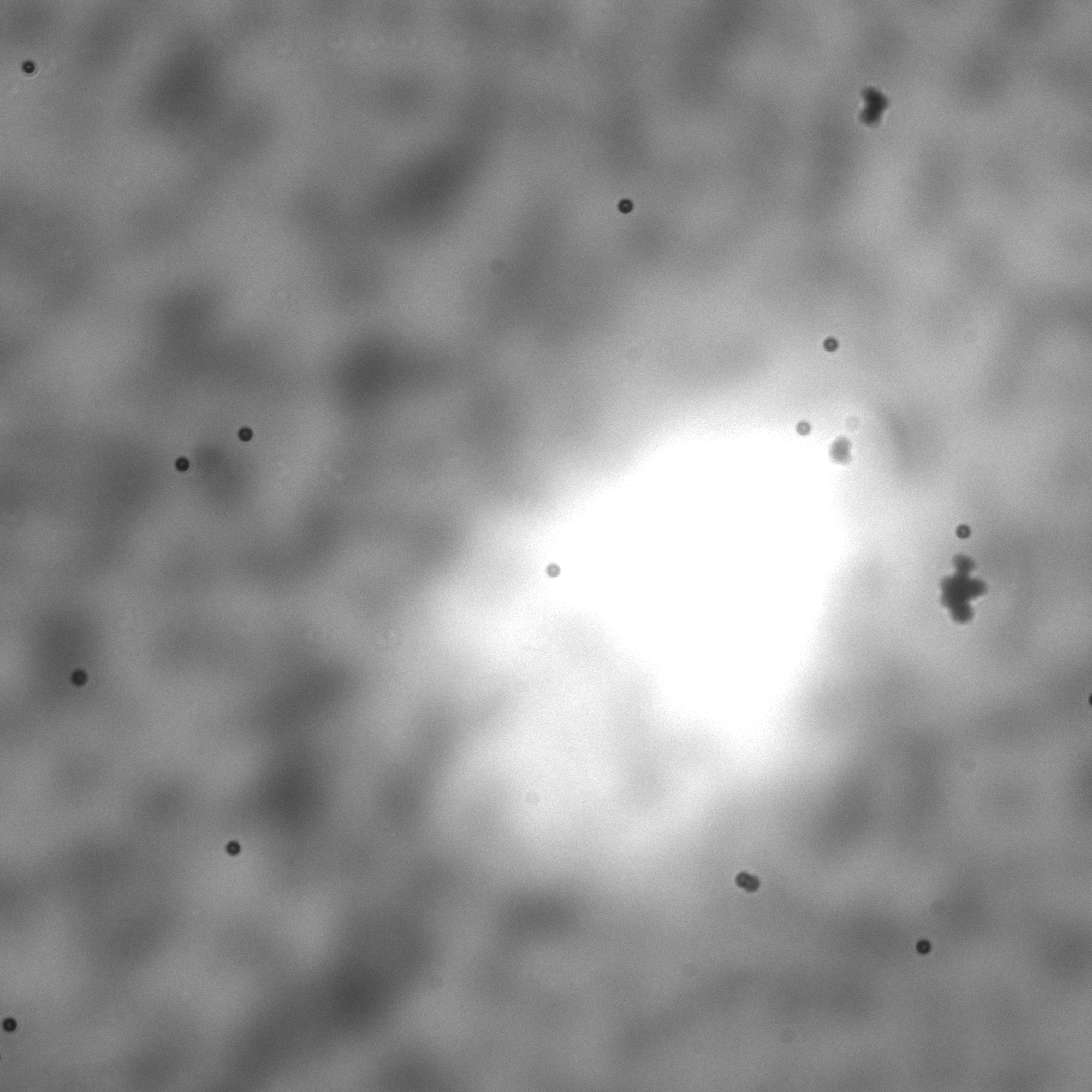

Supplement: Supplementary file 4 — Supplementary Software [file 41467_2023_36373_MOESM4_ESM.zip › analysis software and sample data/CT - Trial Analysis - Sample/22.tiff]

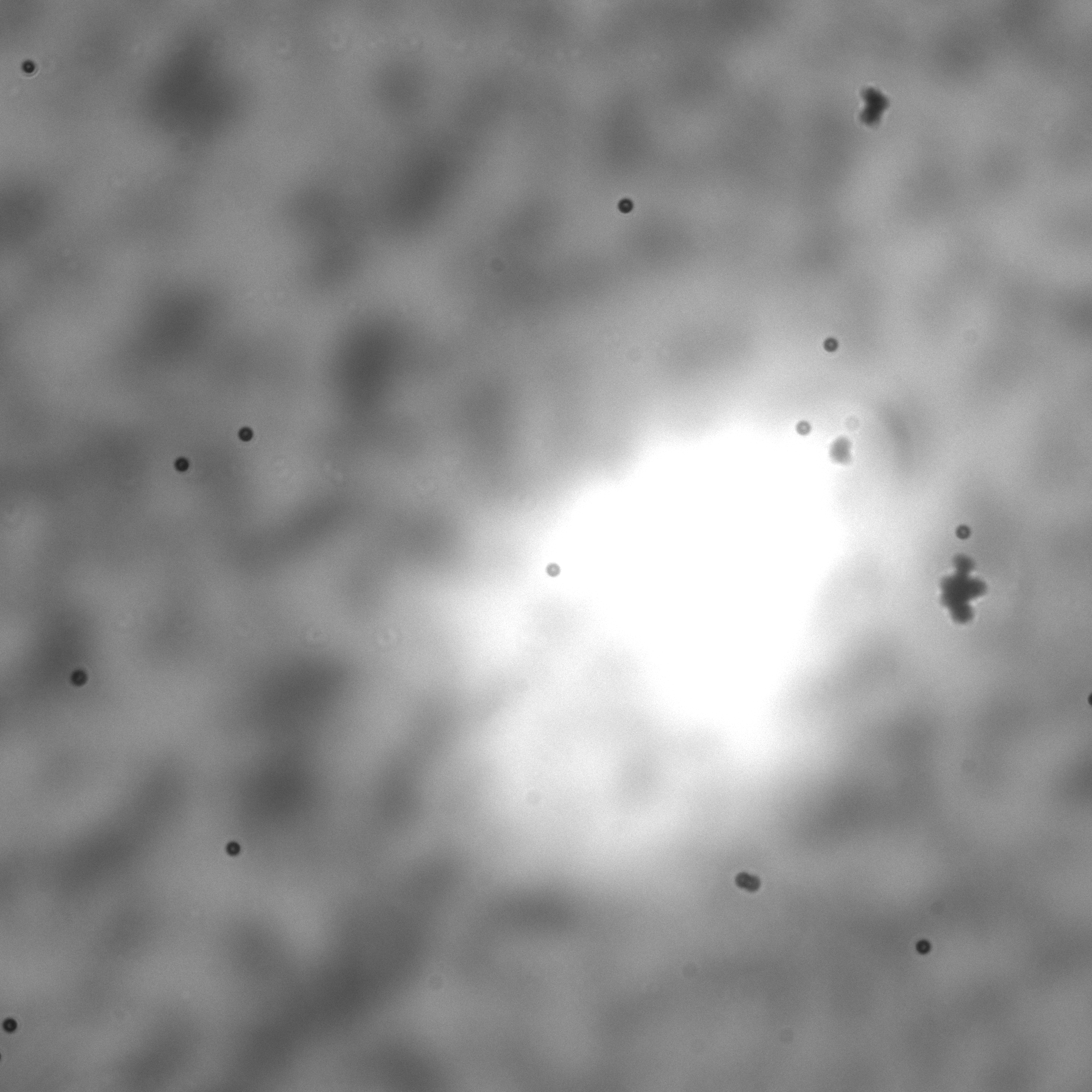

Supplement: Supplementary file 4 — Supplementary Software [file 41467_2023_36373_MOESM4_ESM.zip › analysis software and sample data/CT - Trial Analysis - Sample/23.tiff]

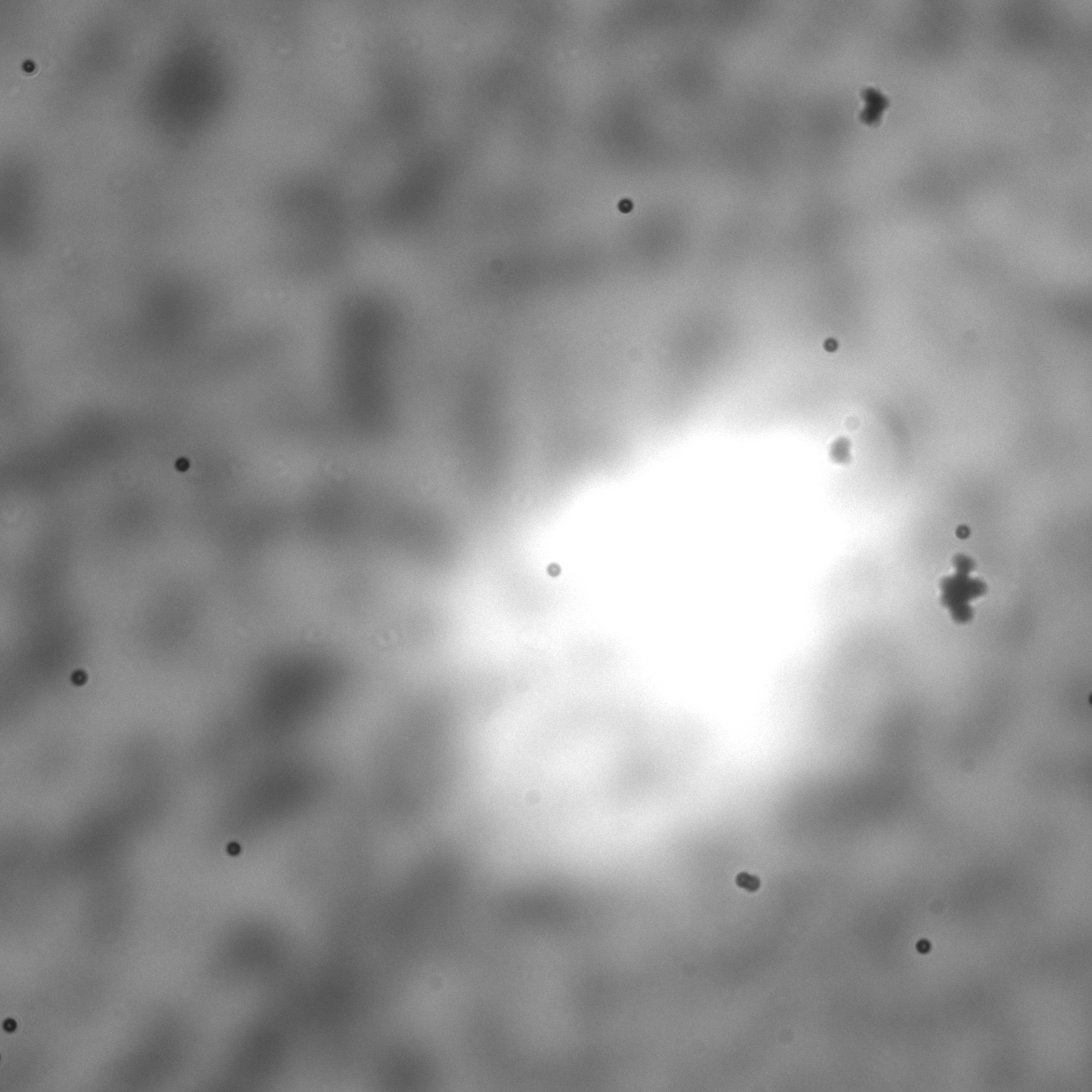

Supplement: Supplementary file 4 — Supplementary Software [file 41467_2023_36373_MOESM4_ESM.zip › analysis software and sample data/CT - Trial Analysis - Sample/24.tiff]

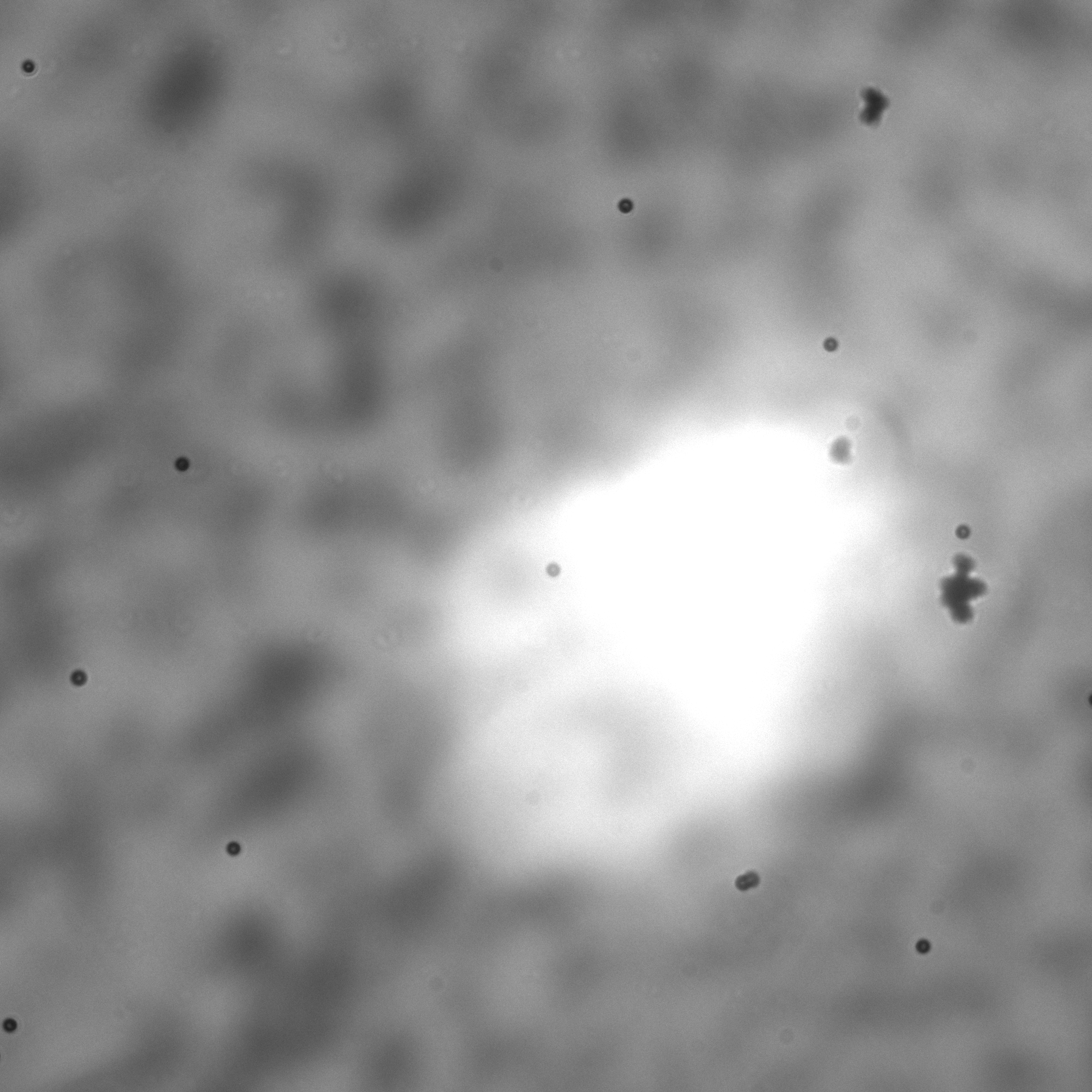

Supplement: Supplementary file 4 — Supplementary Software [file 41467_2023_36373_MOESM4_ESM.zip › analysis software and sample data/CT - Trial Analysis - Sample/25.tiff]

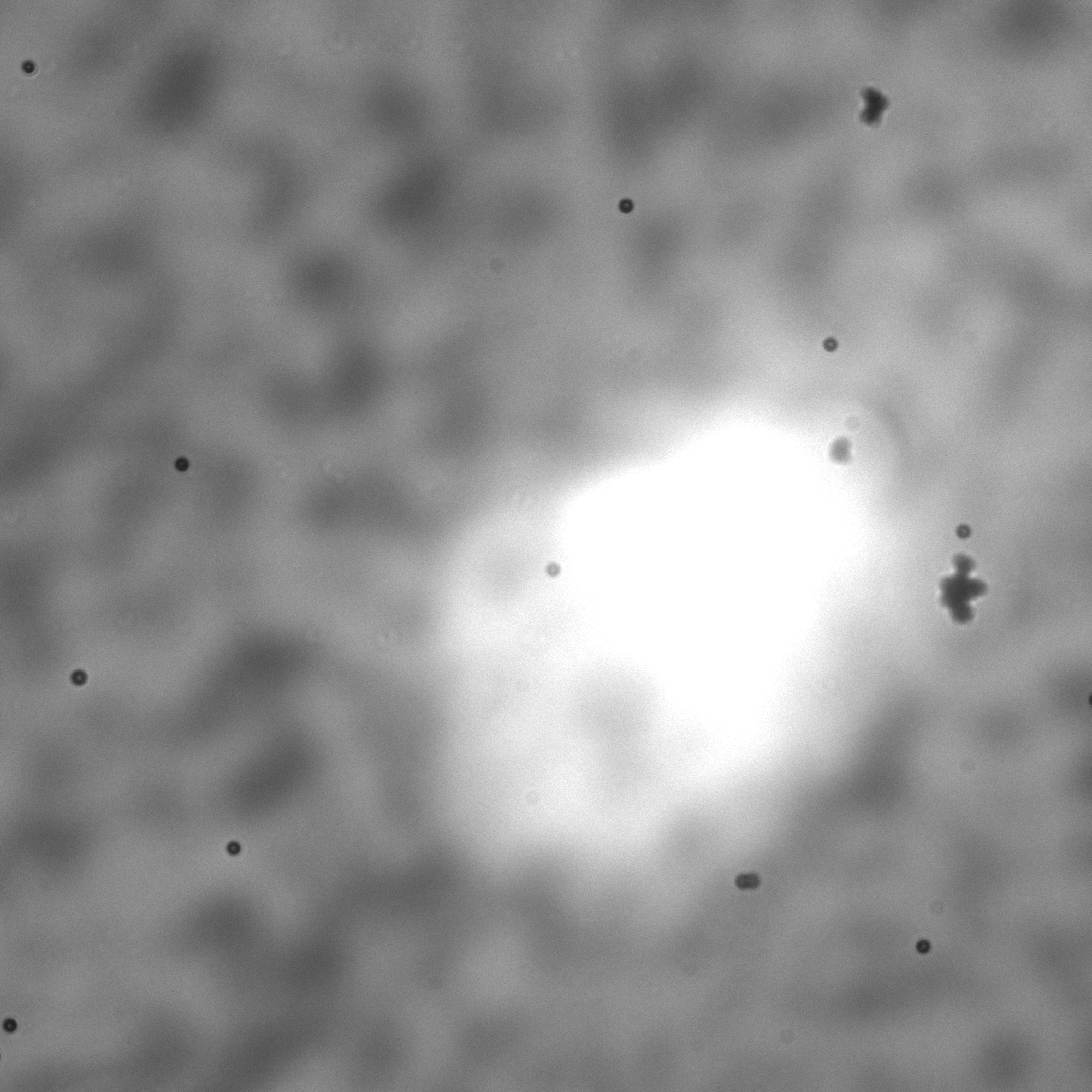

Supplement: Supplementary file 4 — Supplementary Software [file 41467_2023_36373_MOESM4_ESM.zip › analysis software and sample data/CT - Trial Analysis - Sample/26.tiff]

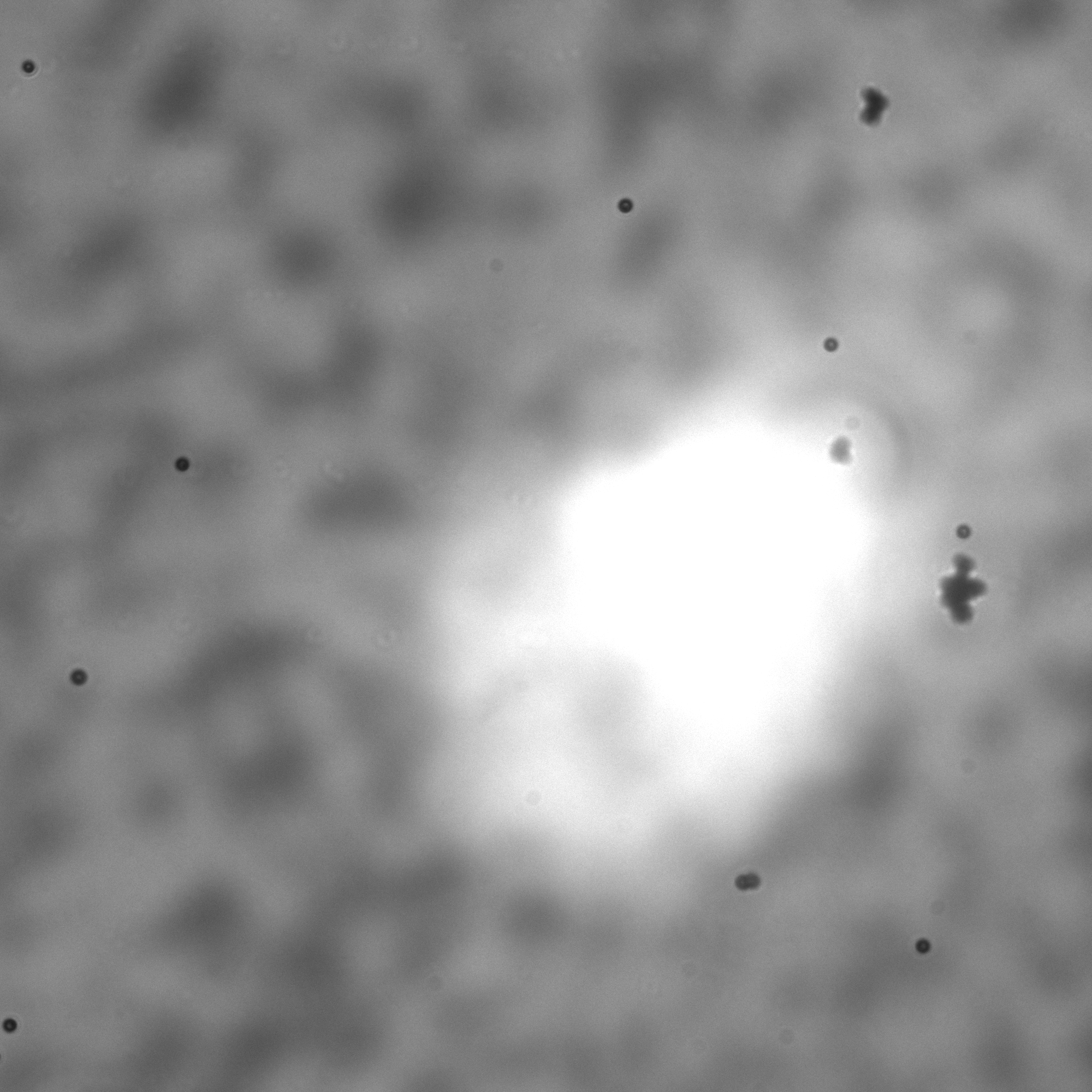

Supplement: Supplementary file 4 — Supplementary Software [file 41467_2023_36373_MOESM4_ESM.zip › analysis software and sample data/CT - Trial Analysis - Sample/27.tiff]

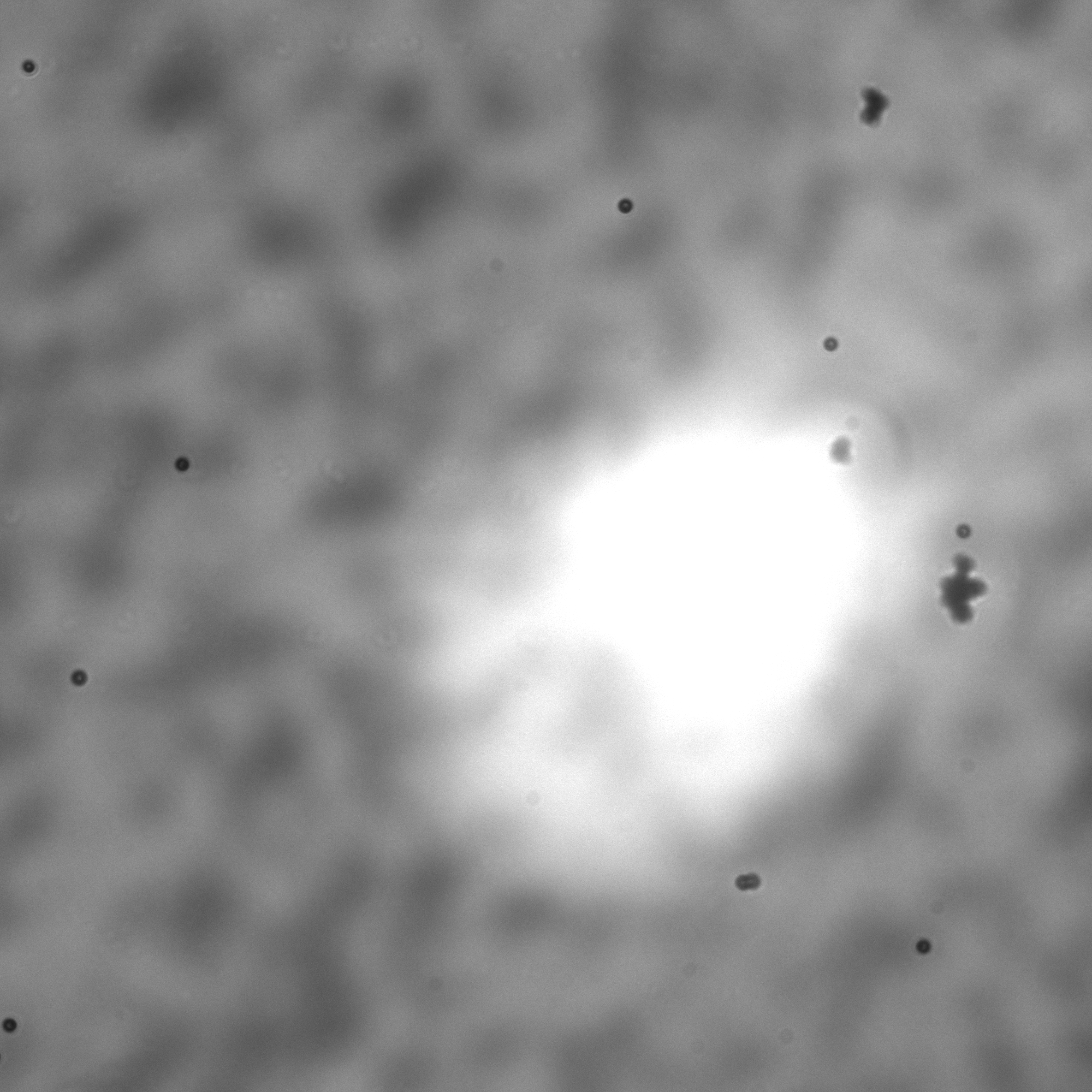

Supplement: Supplementary file 4 — Supplementary Software [file 41467_2023_36373_MOESM4_ESM.zip › analysis software and sample data/CT - Trial Analysis - Sample/28.tiff]

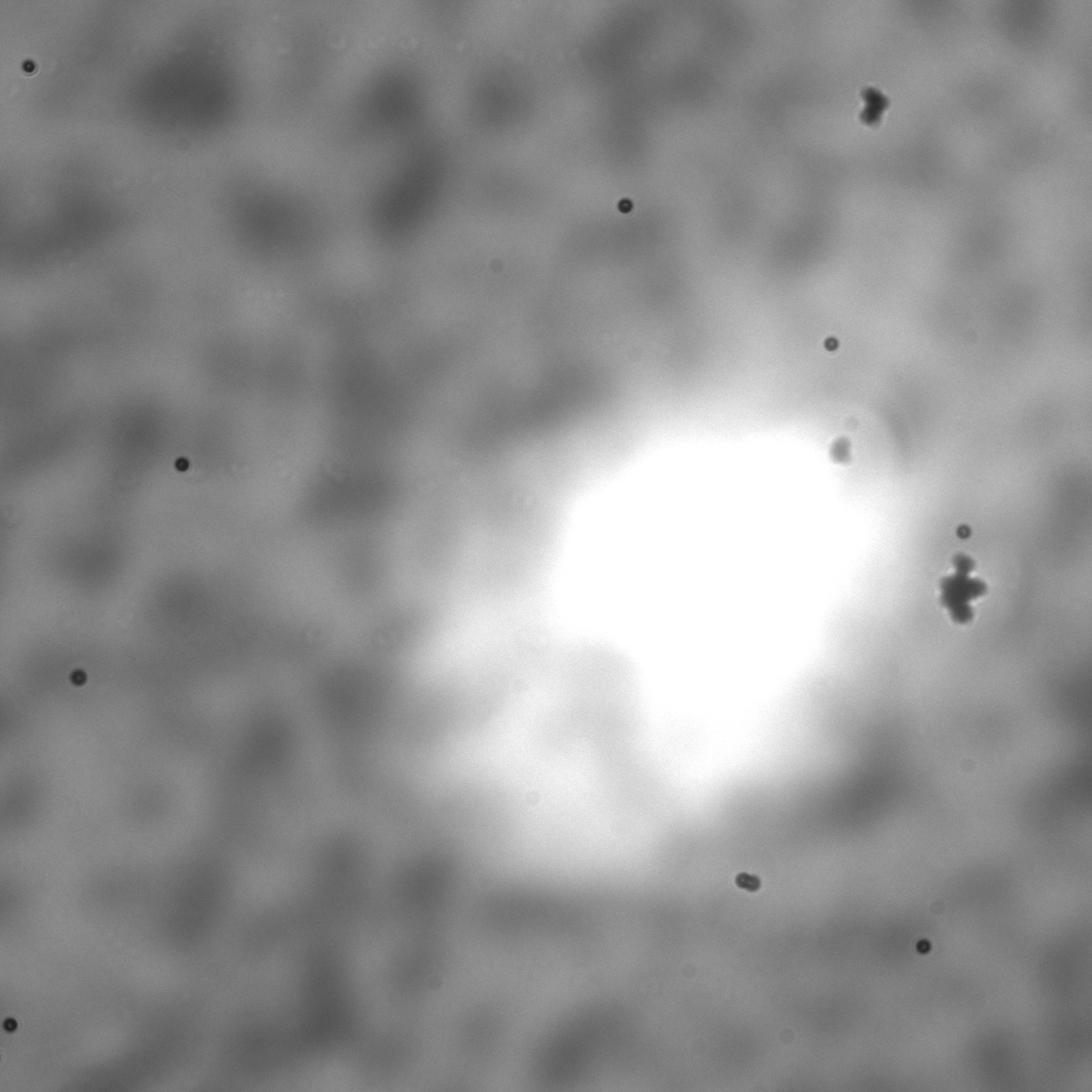

Supplement: Supplementary file 4 — Supplementary Software [file 41467_2023_36373_MOESM4_ESM.zip › analysis software and sample data/CT - Trial Analysis - Sample/29.tiff]

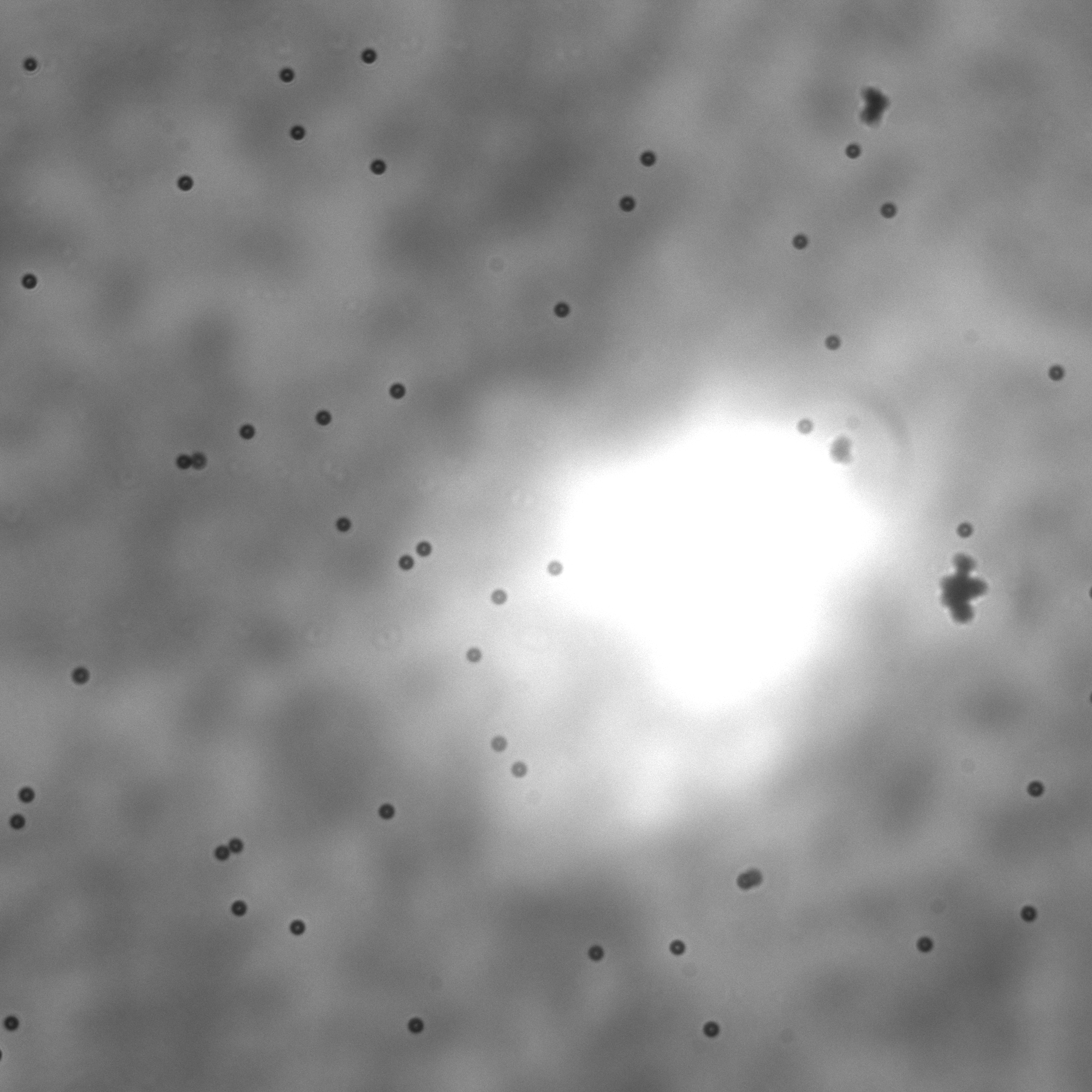

Supplement: Supplementary file 4 — Supplementary Software [file 41467_2023_36373_MOESM4_ESM.zip › analysis software and sample data/CT - Trial Analysis - Sample/3.tiff]

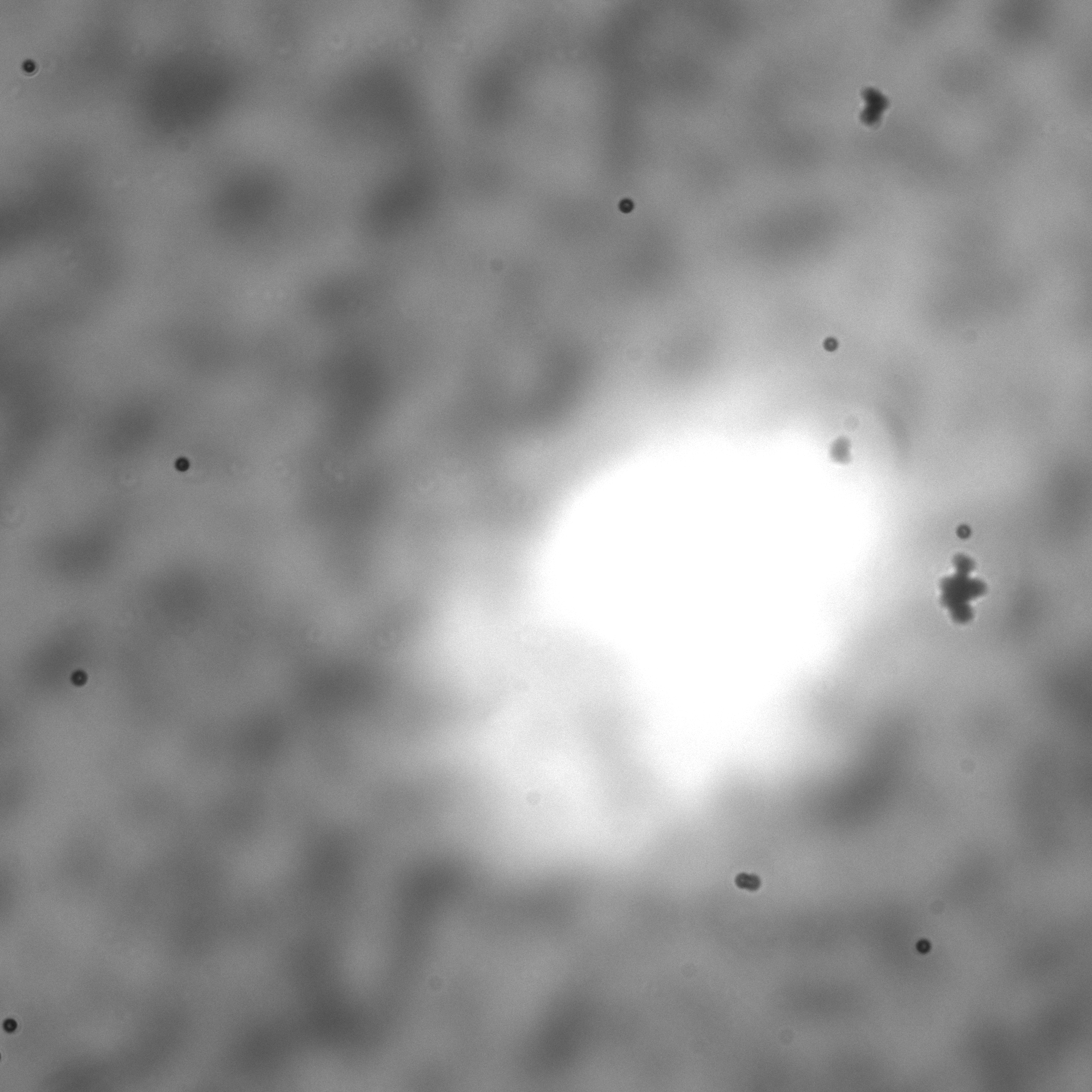

Supplement: Supplementary file 4 — Supplementary Software [file 41467_2023_36373_MOESM4_ESM.zip › analysis software and sample data/CT - Trial Analysis - Sample/30.tiff]

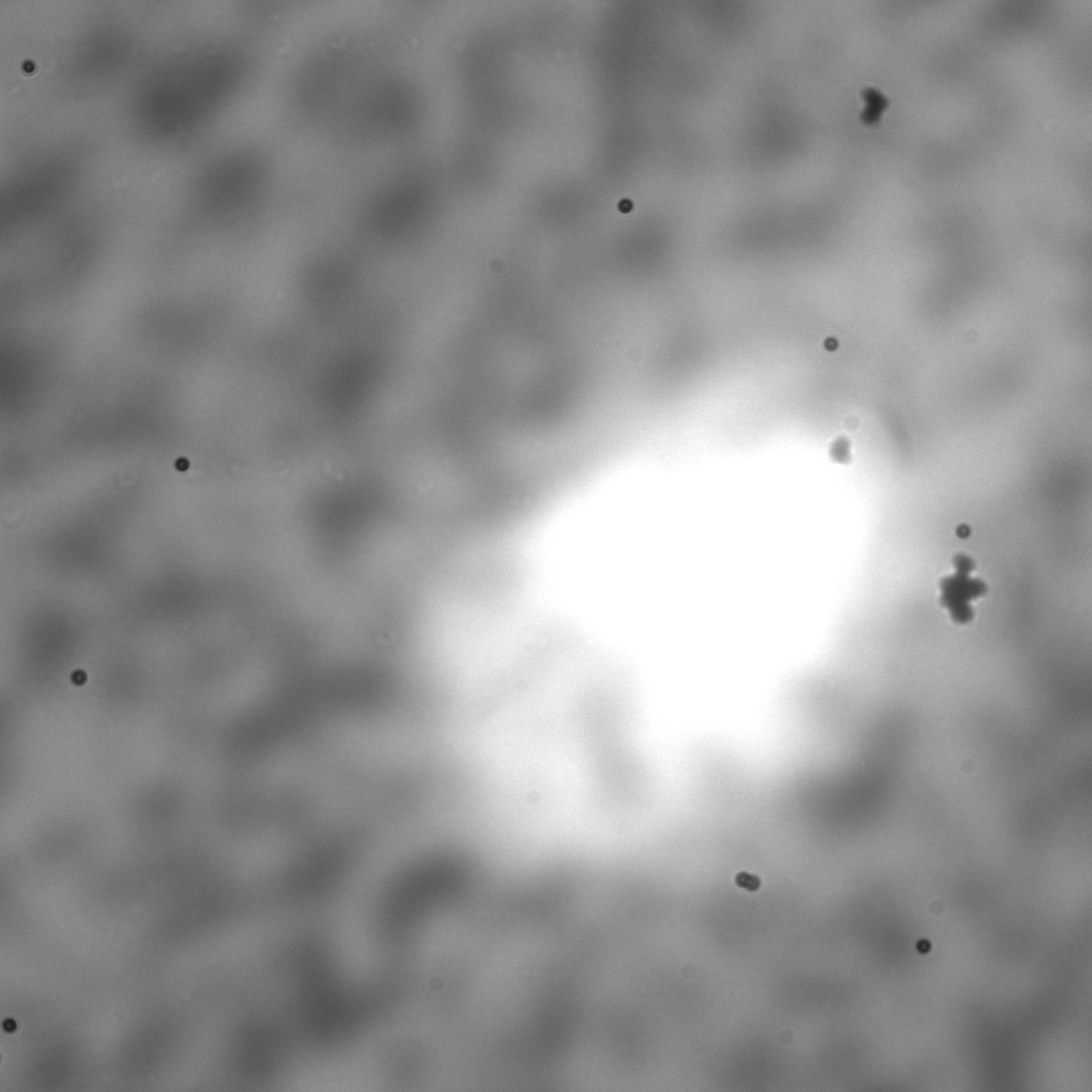

Supplement: Supplementary file 4 — Supplementary Software [file 41467_2023_36373_MOESM4_ESM.zip › analysis software and sample data/CT - Trial Analysis - Sample/31.tiff]

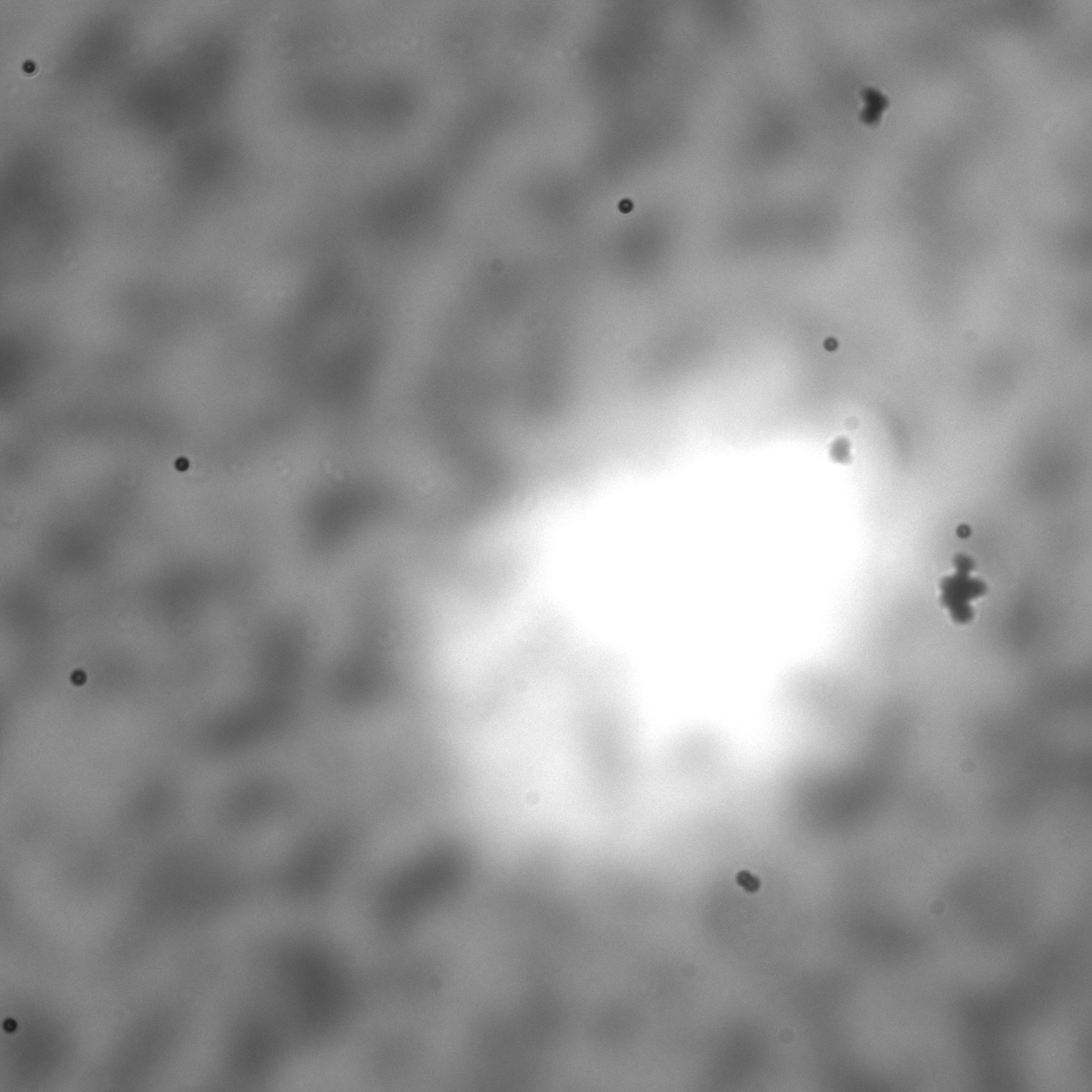

Supplement: Supplementary file 4 — Supplementary Software [file 41467_2023_36373_MOESM4_ESM.zip › analysis software and sample data/CT - Trial Analysis - Sample/32.tiff]

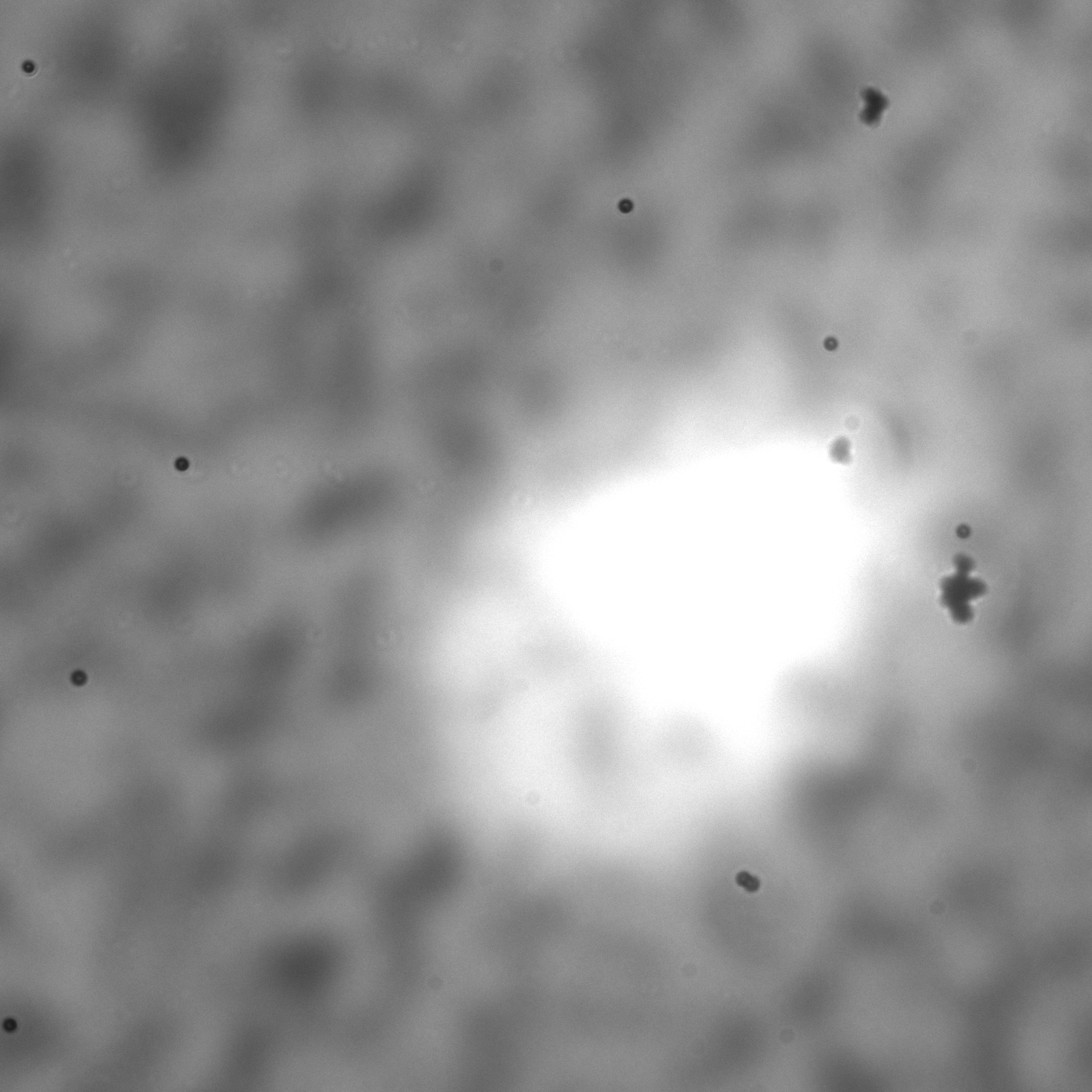

Supplement: Supplementary file 4 — Supplementary Software [file 41467_2023_36373_MOESM4_ESM.zip › analysis software and sample data/CT - Trial Analysis - Sample/33.tiff]

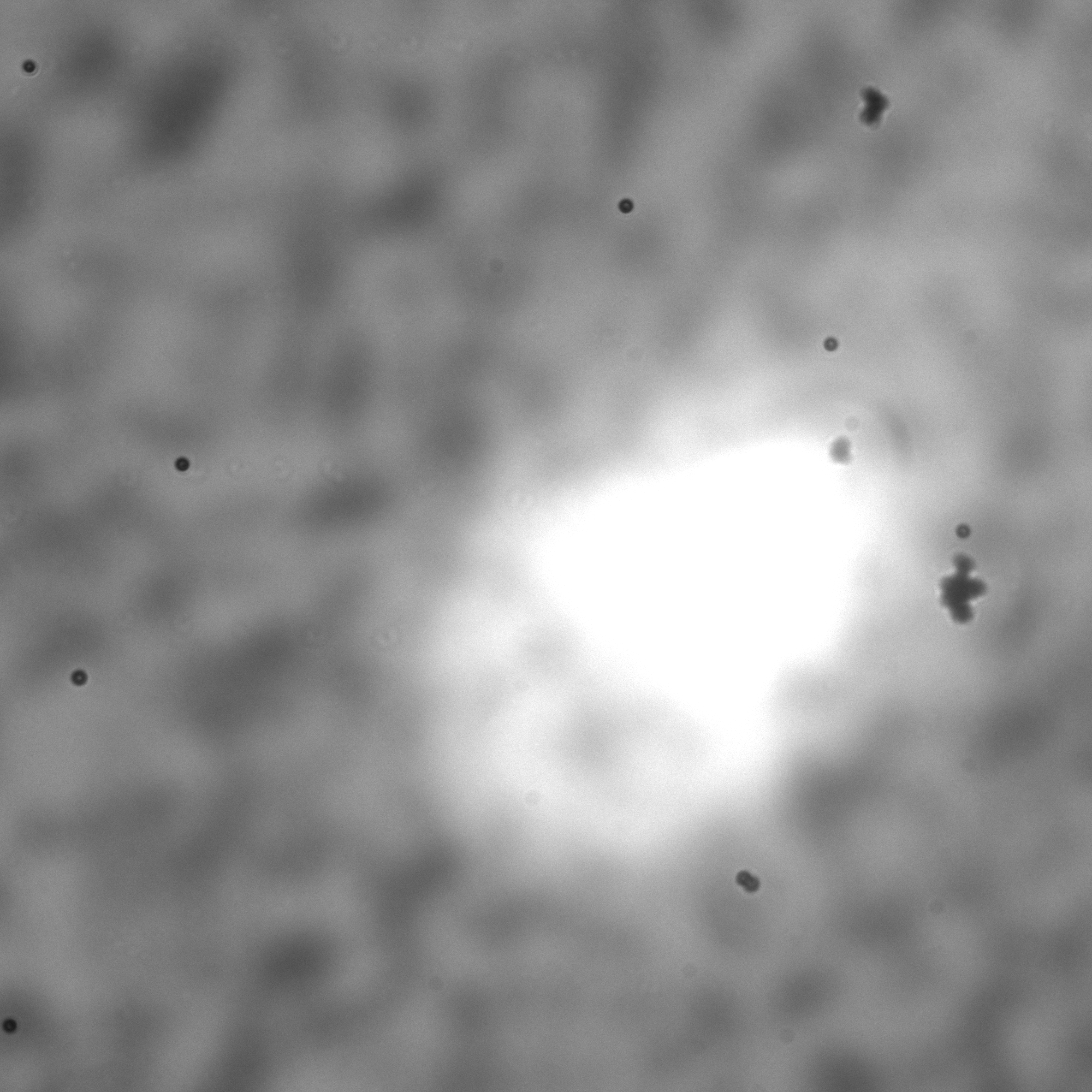

Supplement: Supplementary file 4 — Supplementary Software [file 41467_2023_36373_MOESM4_ESM.zip › analysis software and sample data/CT - Trial Analysis - Sample/34.tiff]

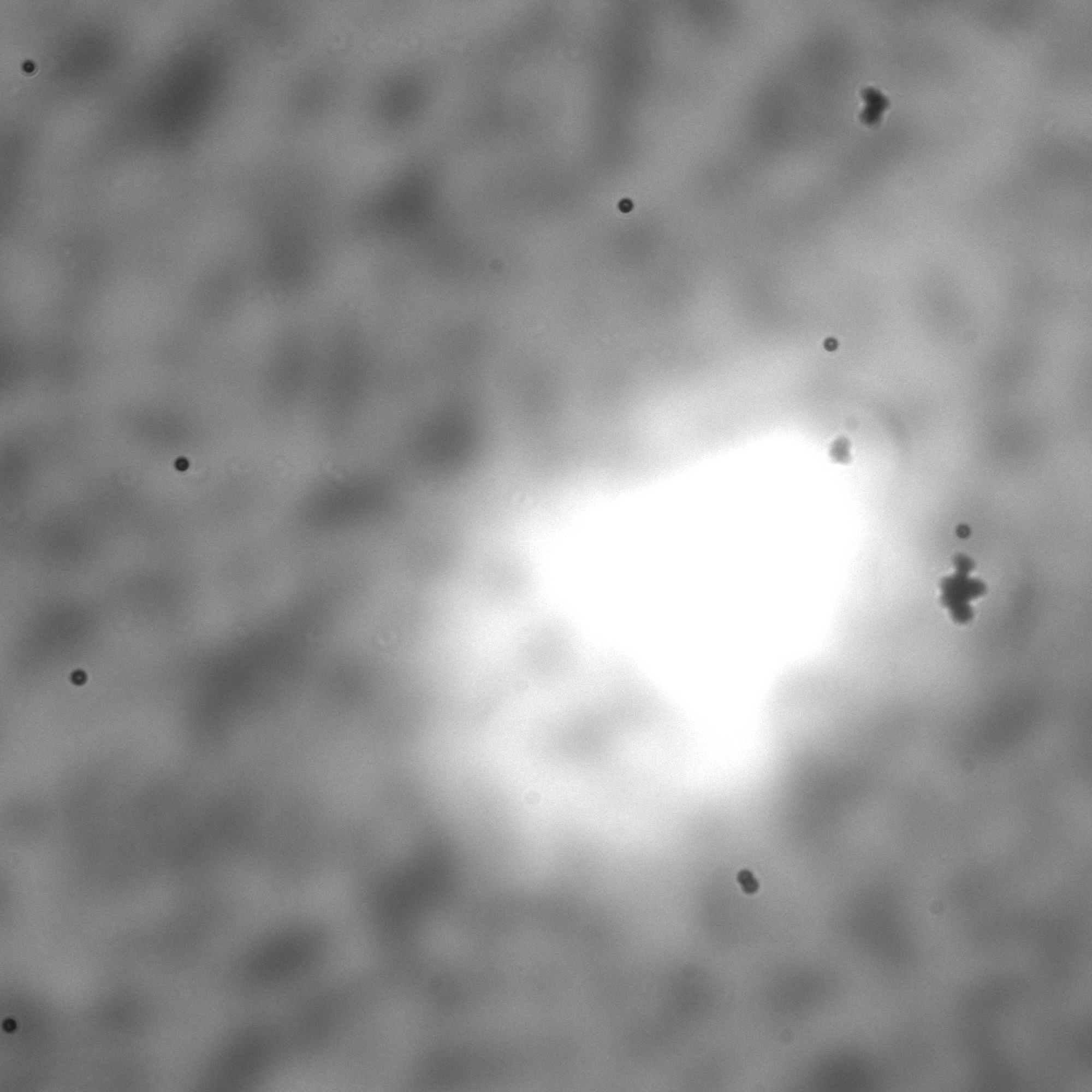

Supplement: Supplementary file 4 — Supplementary Software [file 41467_2023_36373_MOESM4_ESM.zip › analysis software and sample data/CT - Trial Analysis - Sample/35.tiff]

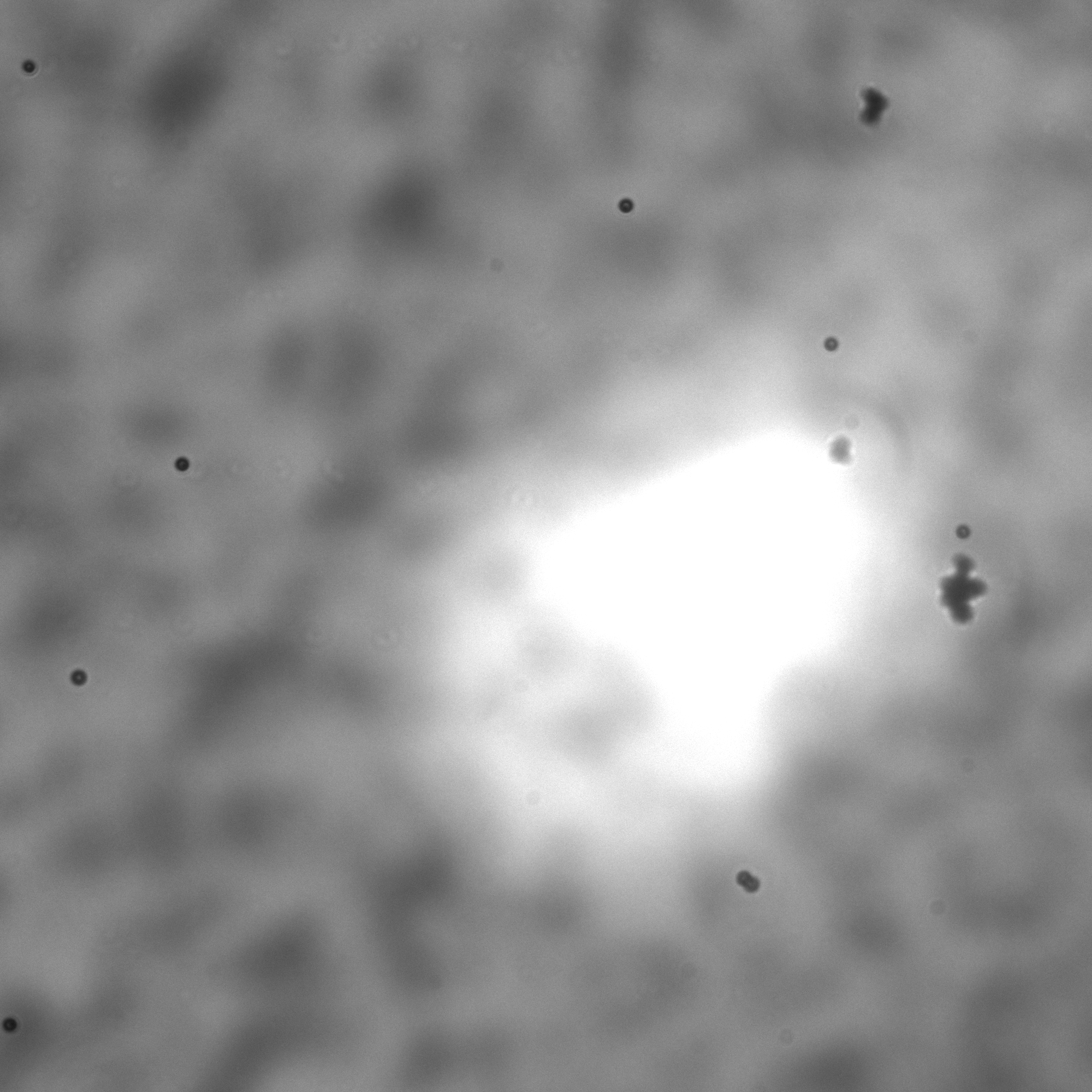

Supplement: Supplementary file 4 — Supplementary Software [file 41467_2023_36373_MOESM4_ESM.zip › analysis software and sample data/CT - Trial Analysis - Sample/36.tiff]

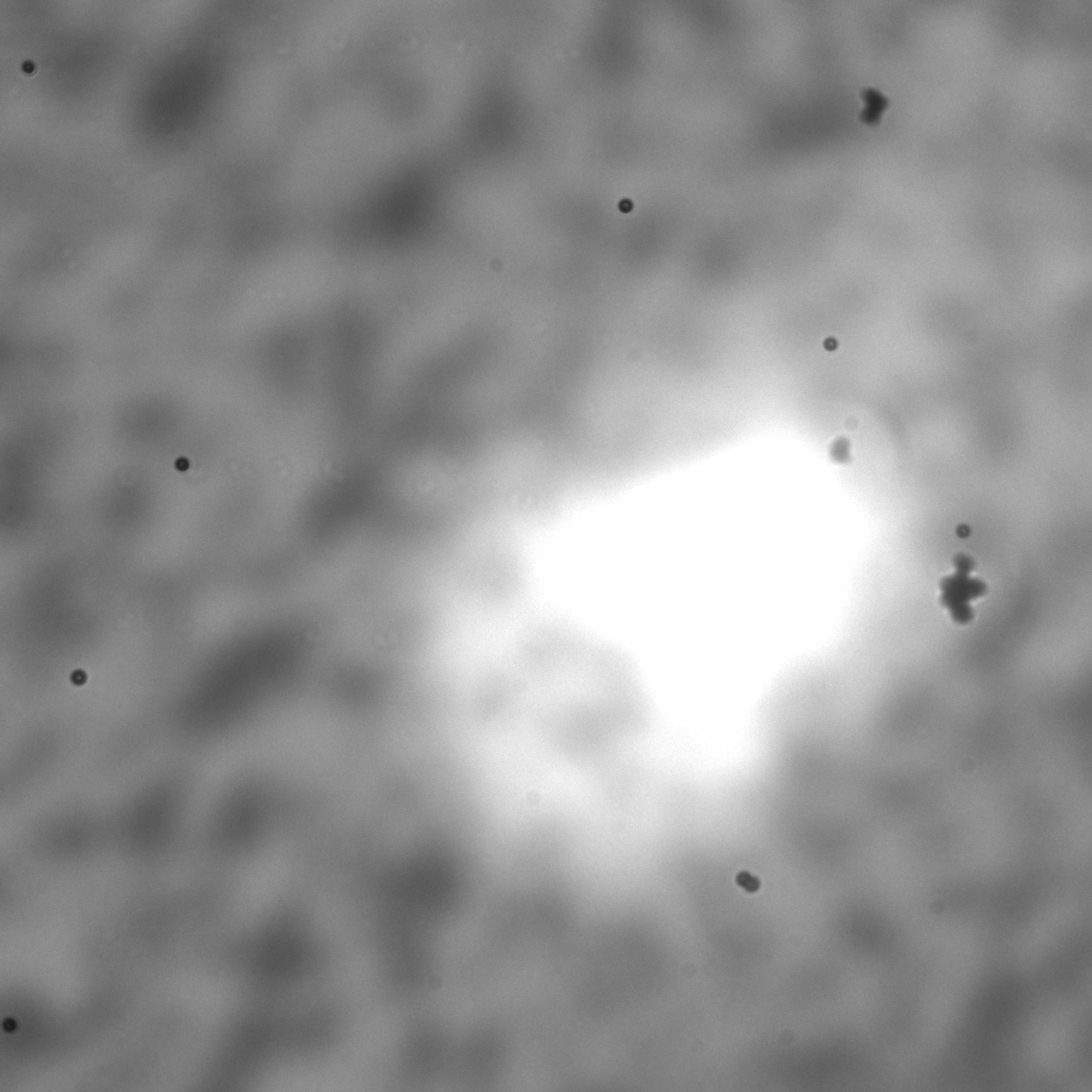

Supplement: Supplementary file 4 — Supplementary Software [file 41467_2023_36373_MOESM4_ESM.zip › analysis software and sample data/CT - Trial Analysis - Sample/37.tiff]

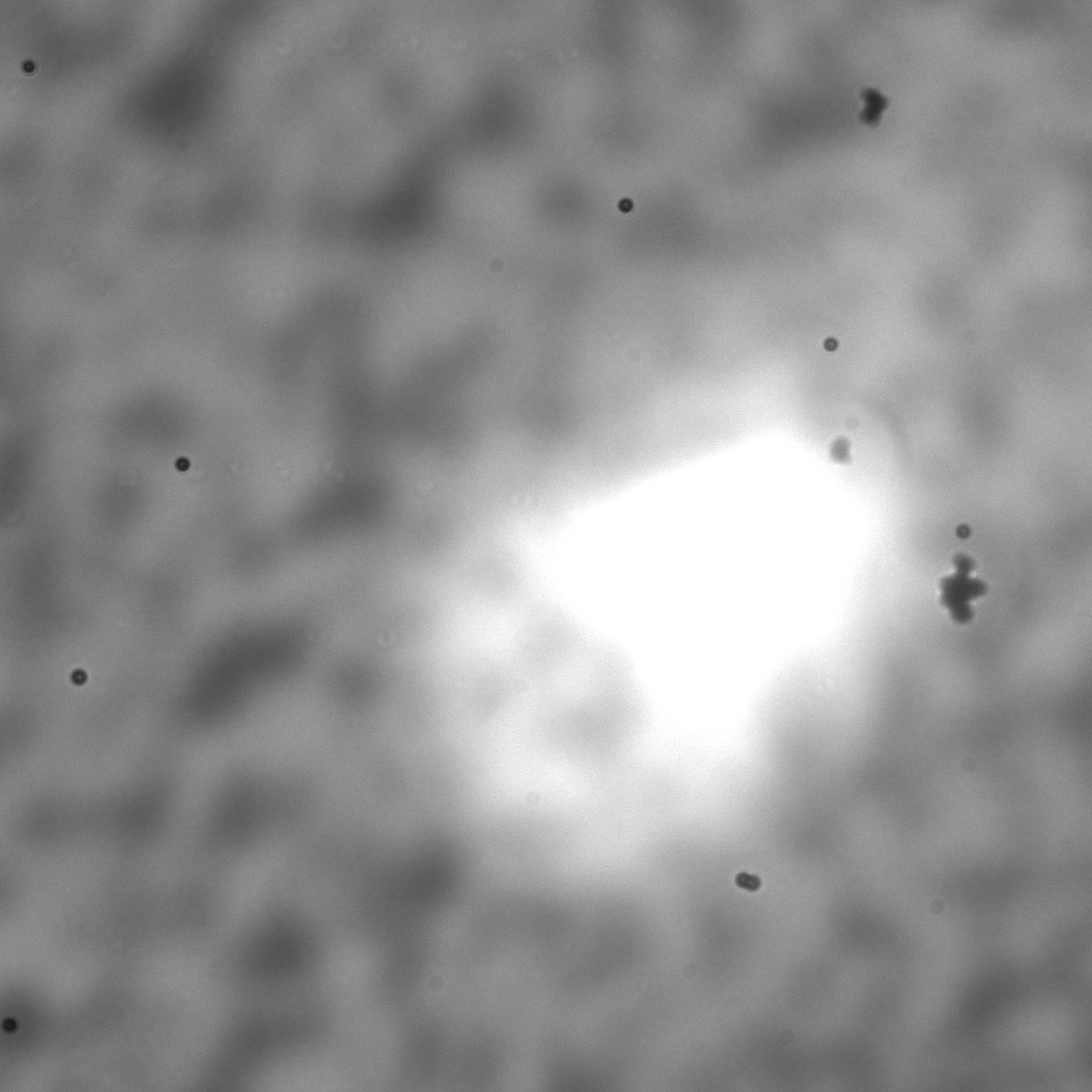

Supplement: Supplementary file 4 — Supplementary Software [file 41467_2023_36373_MOESM4_ESM.zip › analysis software and sample data/CT - Trial Analysis - Sample/38.tiff]

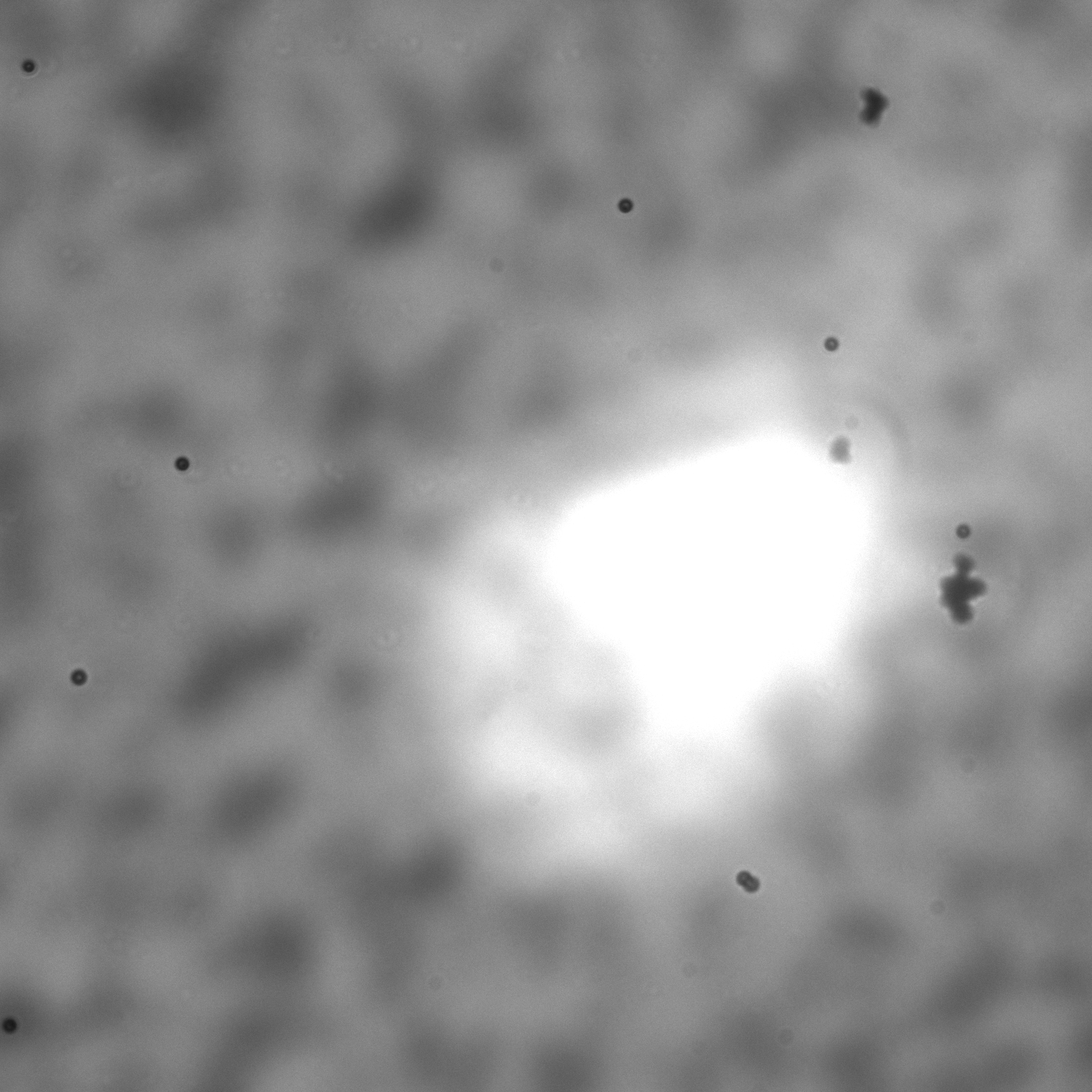

Supplement: Supplementary file 4 — Supplementary Software [file 41467_2023_36373_MOESM4_ESM.zip › analysis software and sample data/CT - Trial Analysis - Sample/39.tiff]

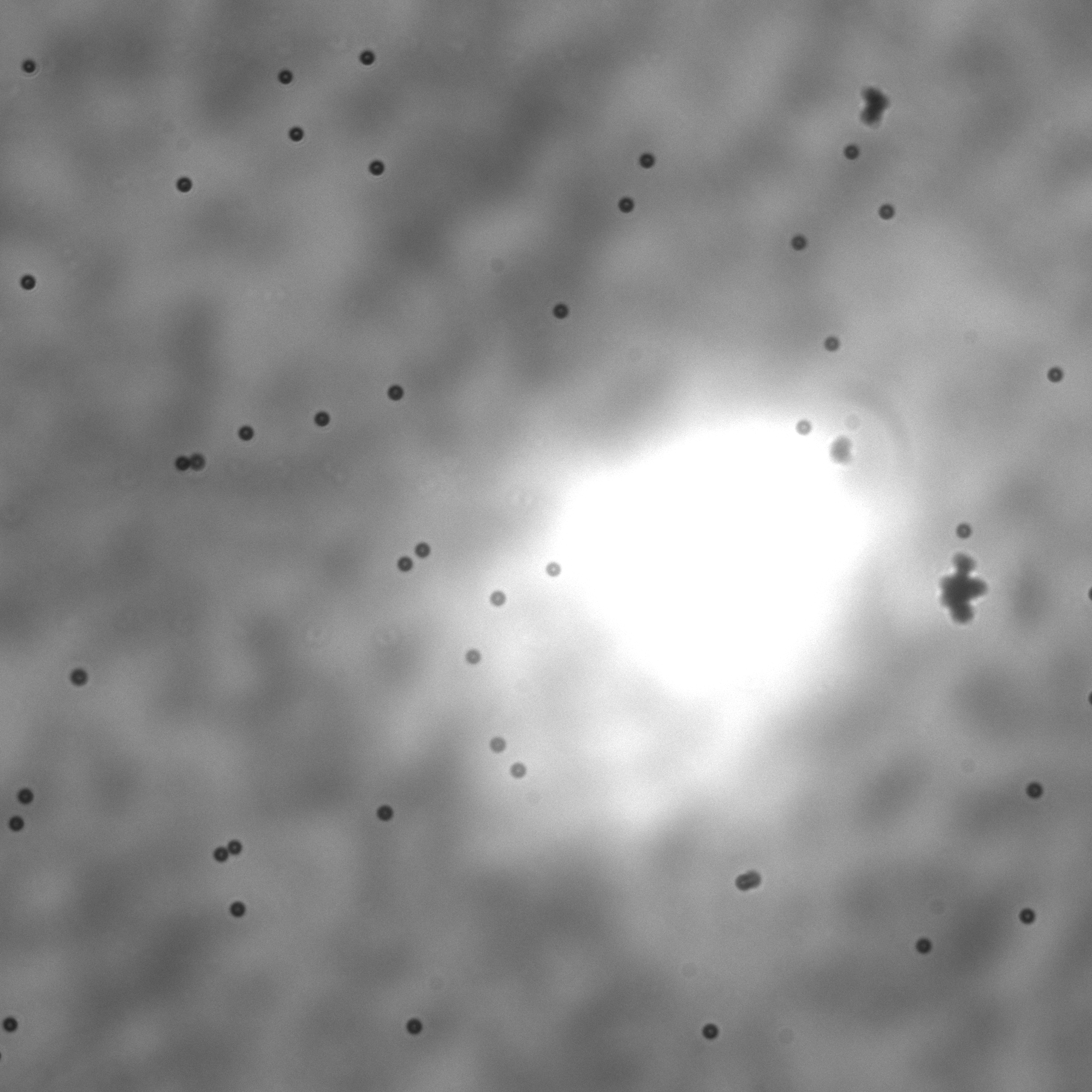

Supplement: Supplementary file 4 — Supplementary Software [file 41467_2023_36373_MOESM4_ESM.zip › analysis software and sample data/CT - Trial Analysis - Sample/4.tiff]

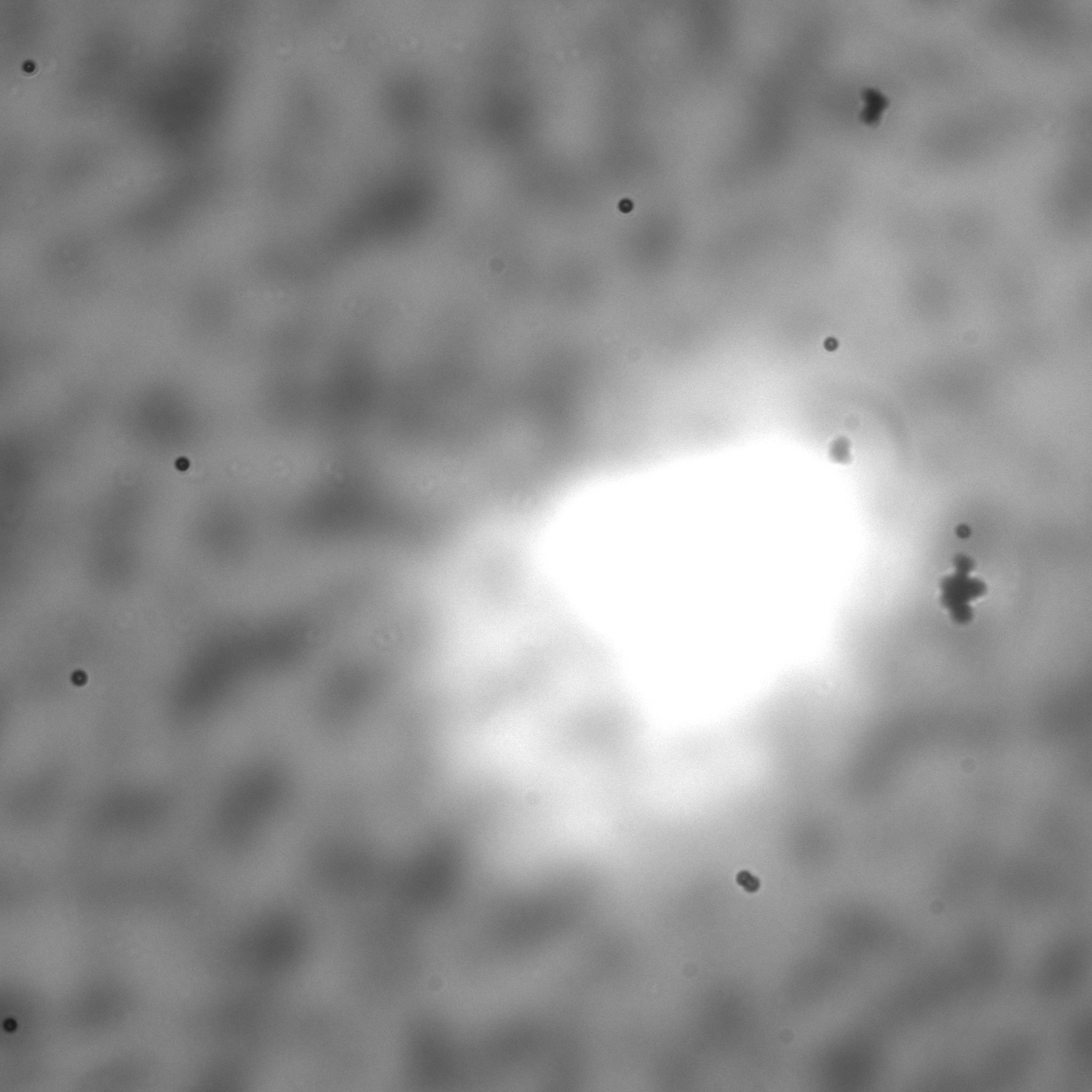

Supplement: Supplementary file 4 — Supplementary Software [file 41467_2023_36373_MOESM4_ESM.zip › analysis software and sample data/CT - Trial Analysis - Sample/40.tiff]

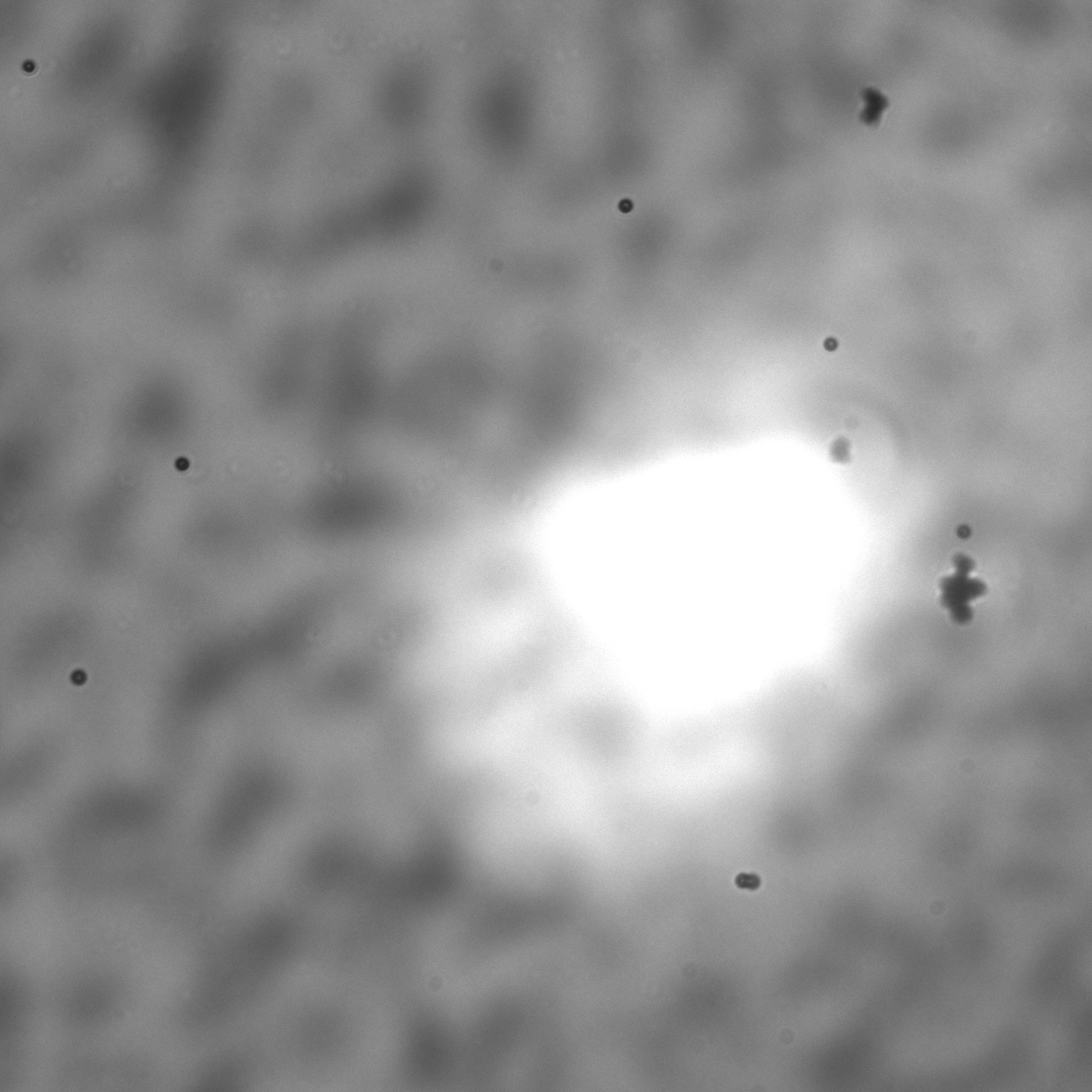

Supplement: Supplementary file 4 — Supplementary Software [file 41467_2023_36373_MOESM4_ESM.zip › analysis software and sample data/CT - Trial Analysis - Sample/41.tiff]

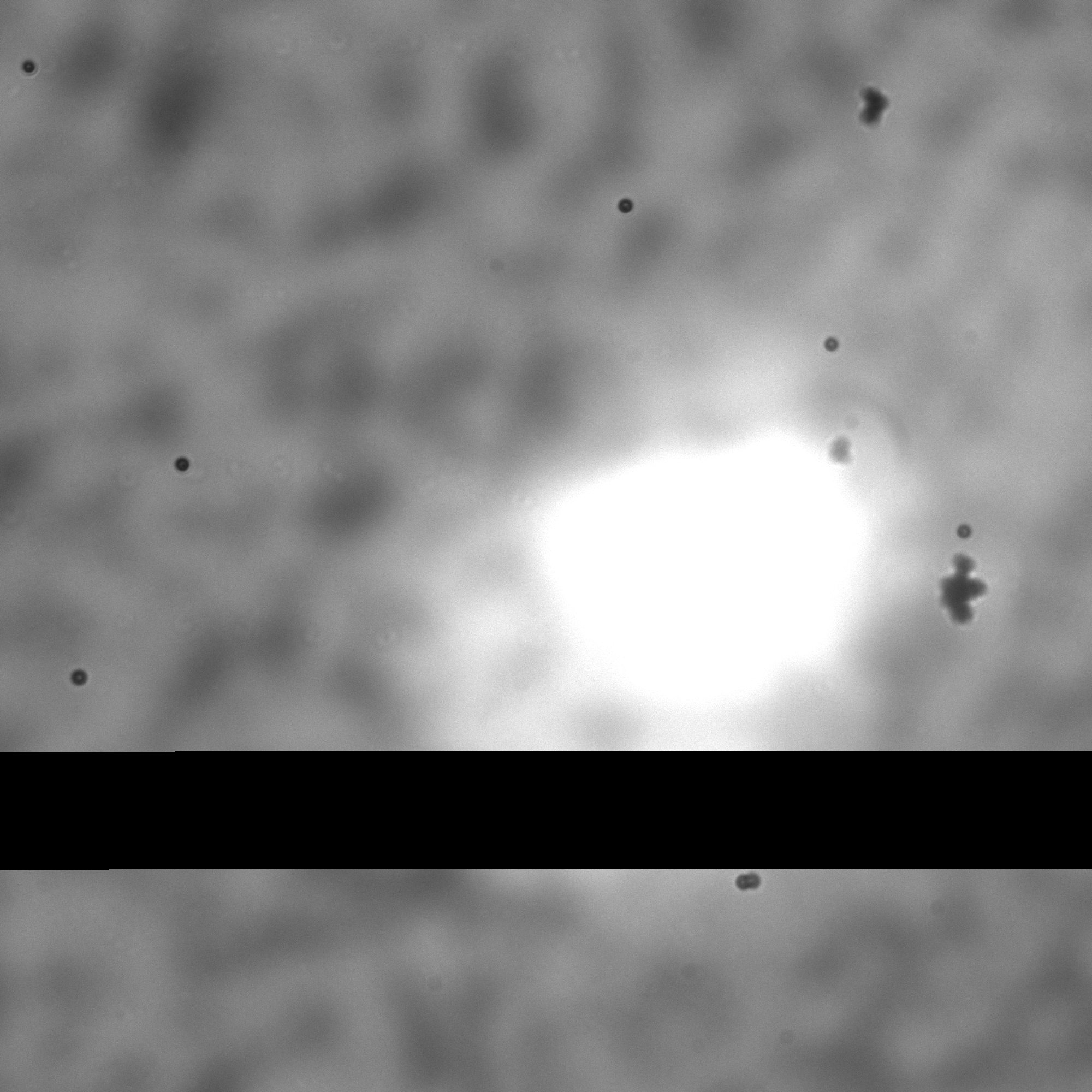

Supplement: Supplementary file 4 — Supplementary Software [file 41467_2023_36373_MOESM4_ESM.zip › analysis software and sample data/CT - Trial Analysis - Sample/42.tiff]

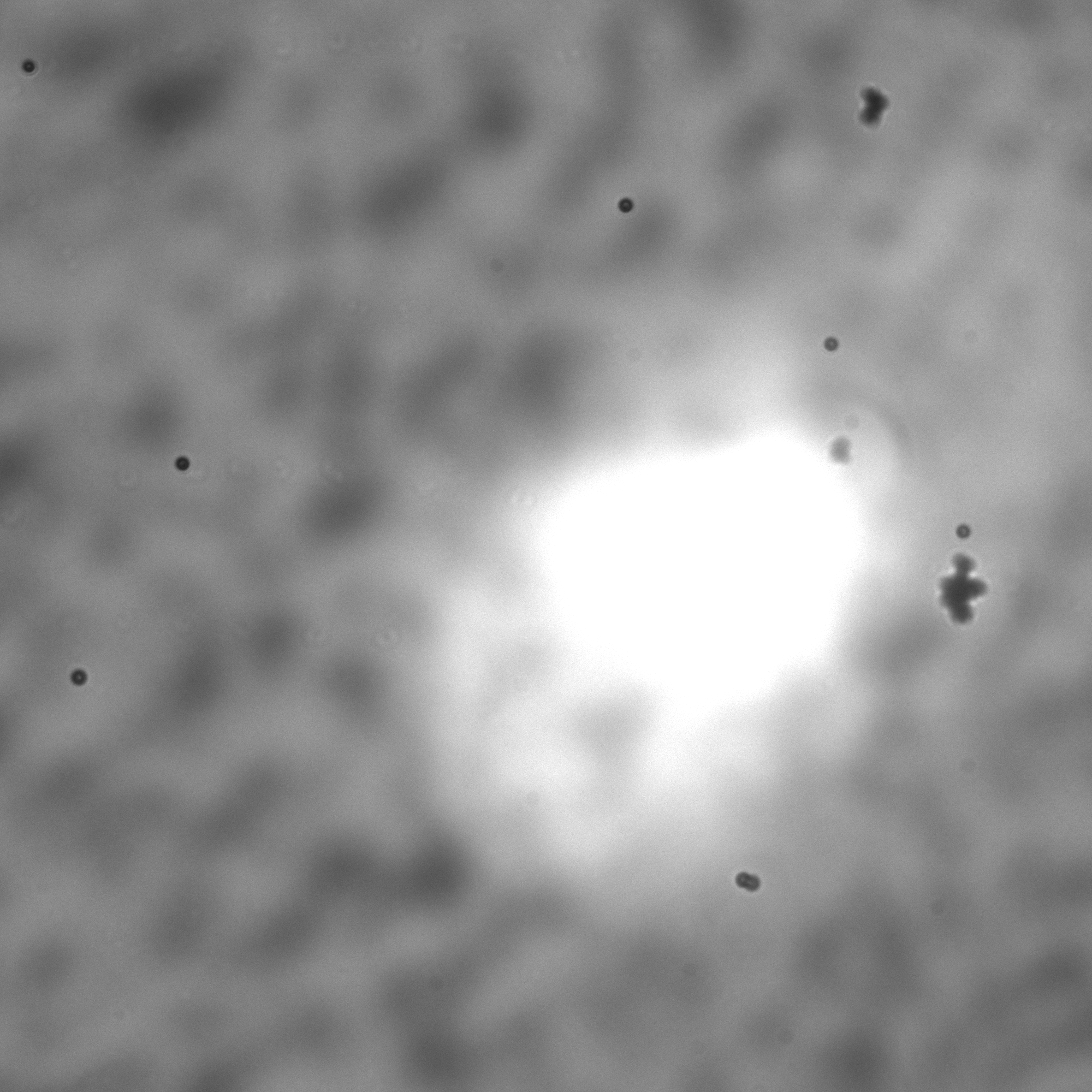

Supplement: Supplementary file 4 — Supplementary Software [file 41467_2023_36373_MOESM4_ESM.zip › analysis software and sample data/CT - Trial Analysis - Sample/43.tiff]

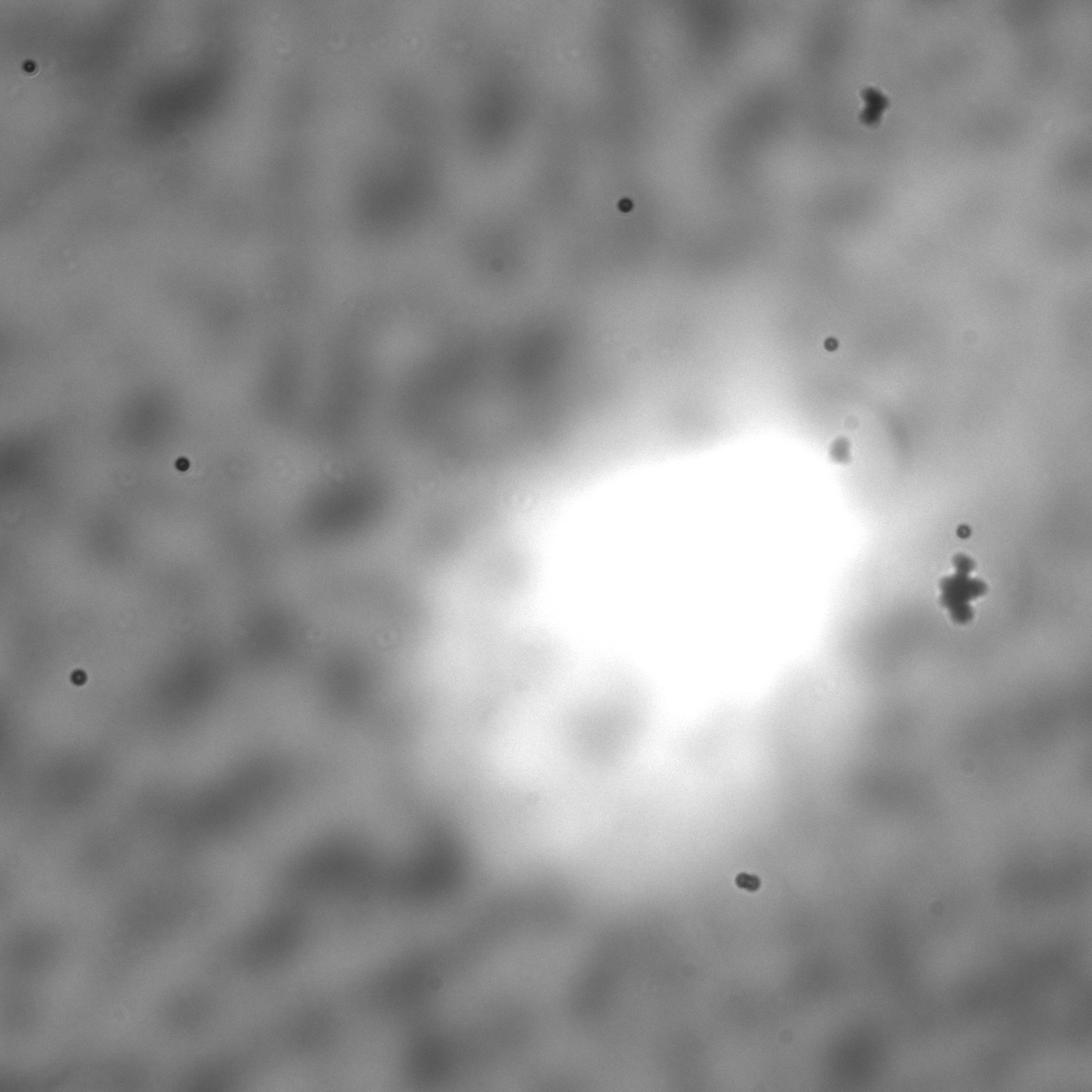

Supplement: Supplementary file 4 — Supplementary Software [file 41467_2023_36373_MOESM4_ESM.zip › analysis software and sample data/CT - Trial Analysis - Sample/44.tiff]

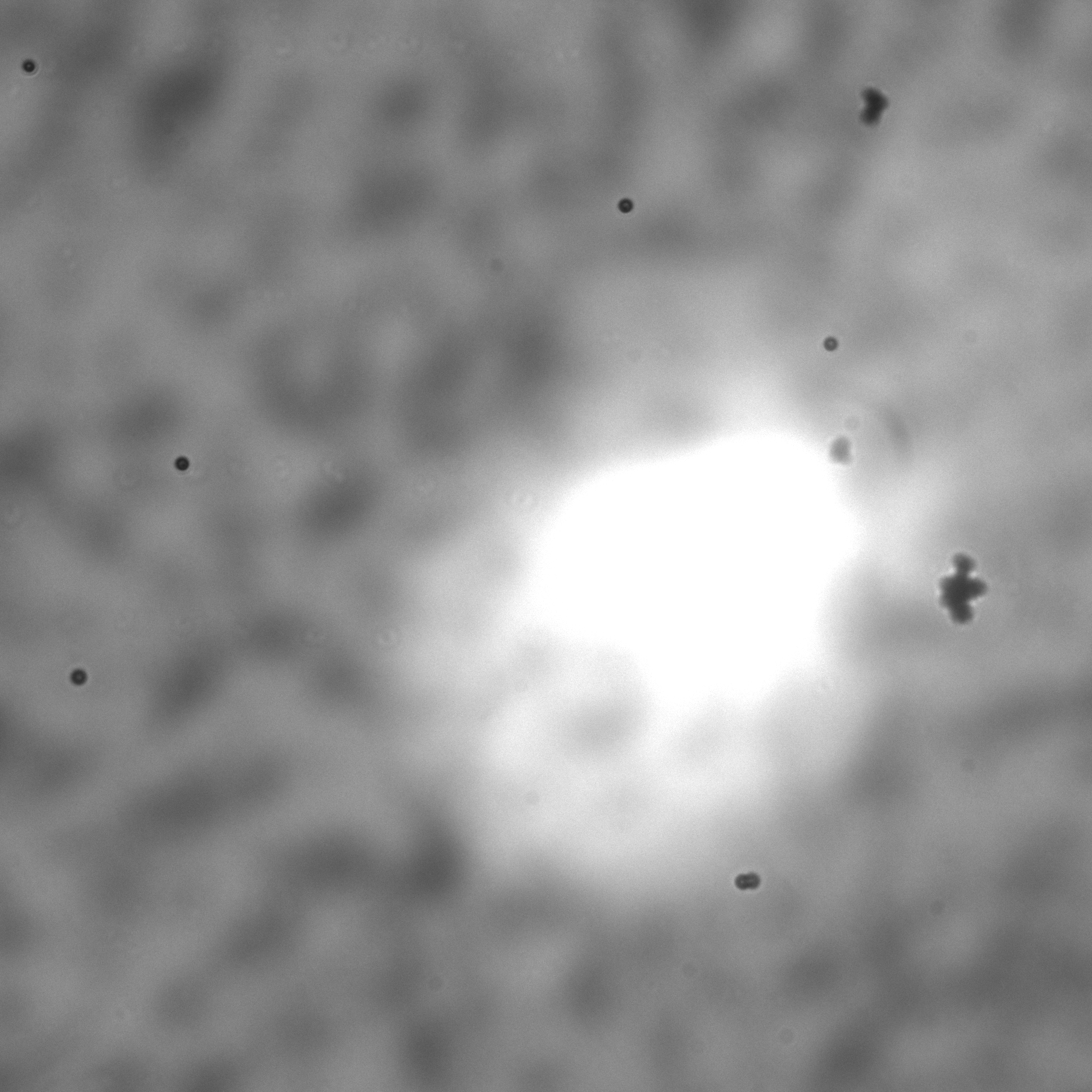

Supplement: Supplementary file 4 — Supplementary Software [file 41467_2023_36373_MOESM4_ESM.zip › analysis software and sample data/CT - Trial Analysis - Sample/45.tiff]

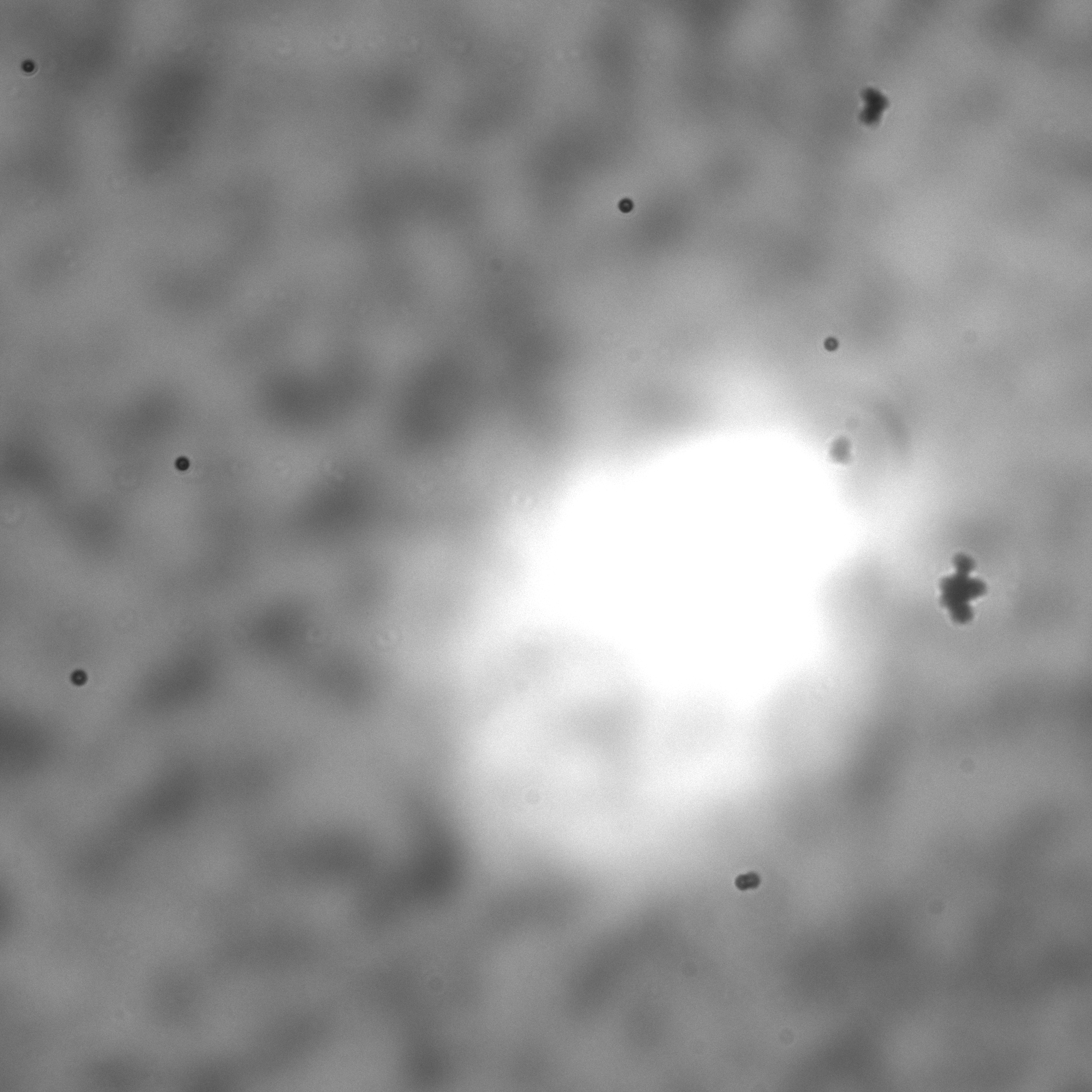

Supplement: Supplementary file 4 — Supplementary Software [file 41467_2023_36373_MOESM4_ESM.zip › analysis software and sample data/CT - Trial Analysis - Sample/46.tiff]

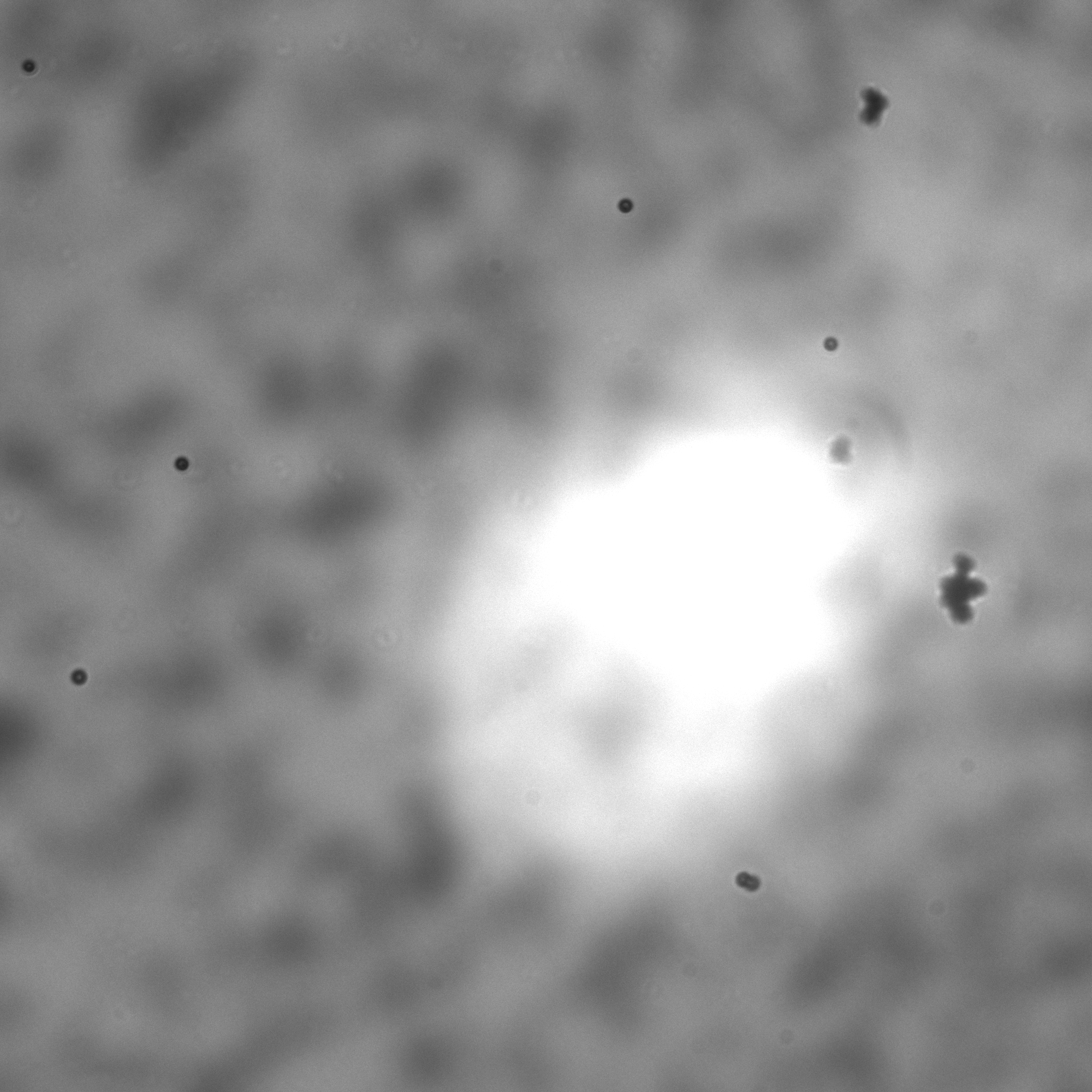

Supplement: Supplementary file 4 — Supplementary Software [file 41467_2023_36373_MOESM4_ESM.zip › analysis software and sample data/CT - Trial Analysis - Sample/47.tiff]

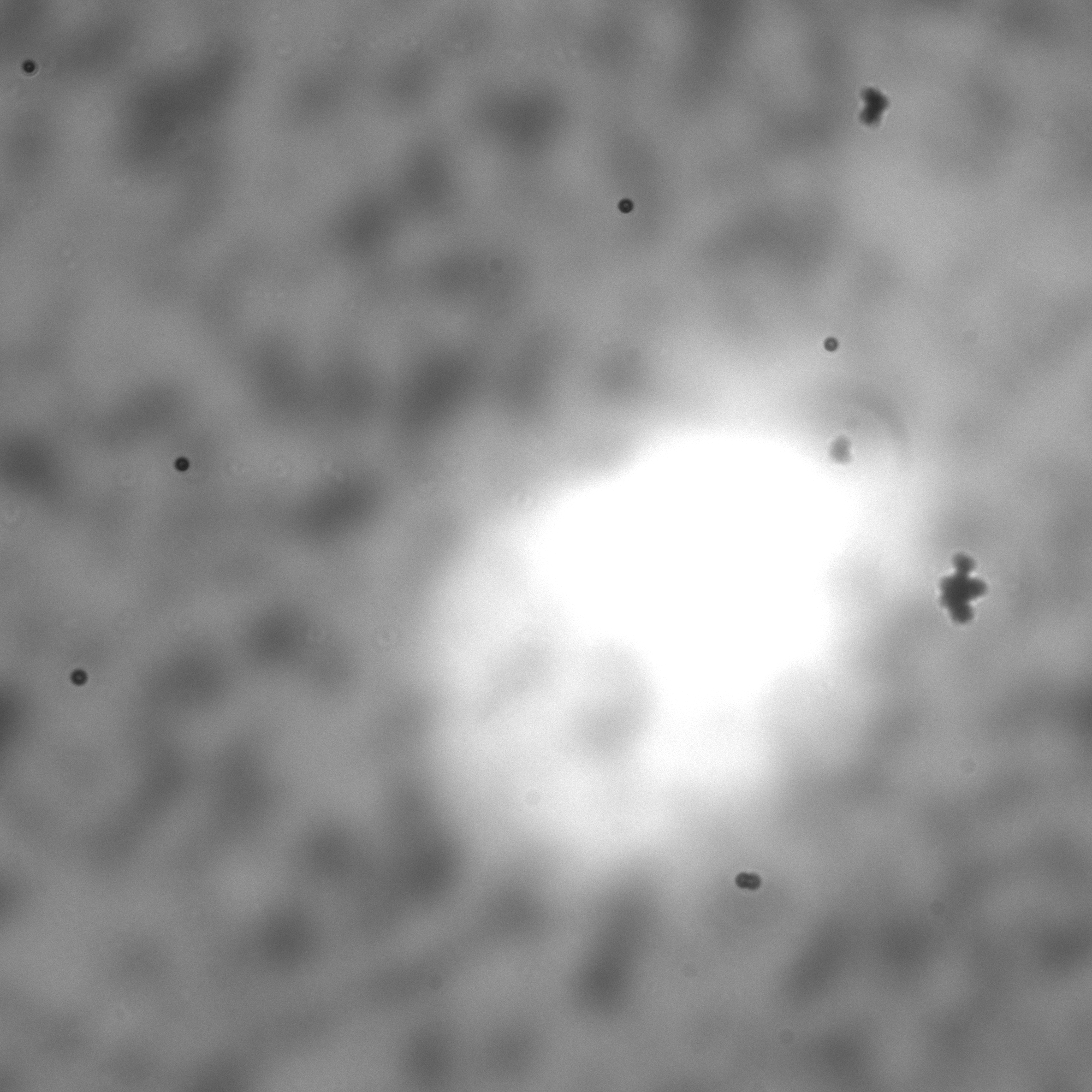

Supplement: Supplementary file 4 — Supplementary Software [file 41467_2023_36373_MOESM4_ESM.zip › analysis software and sample data/CT - Trial Analysis - Sample/48.tiff]

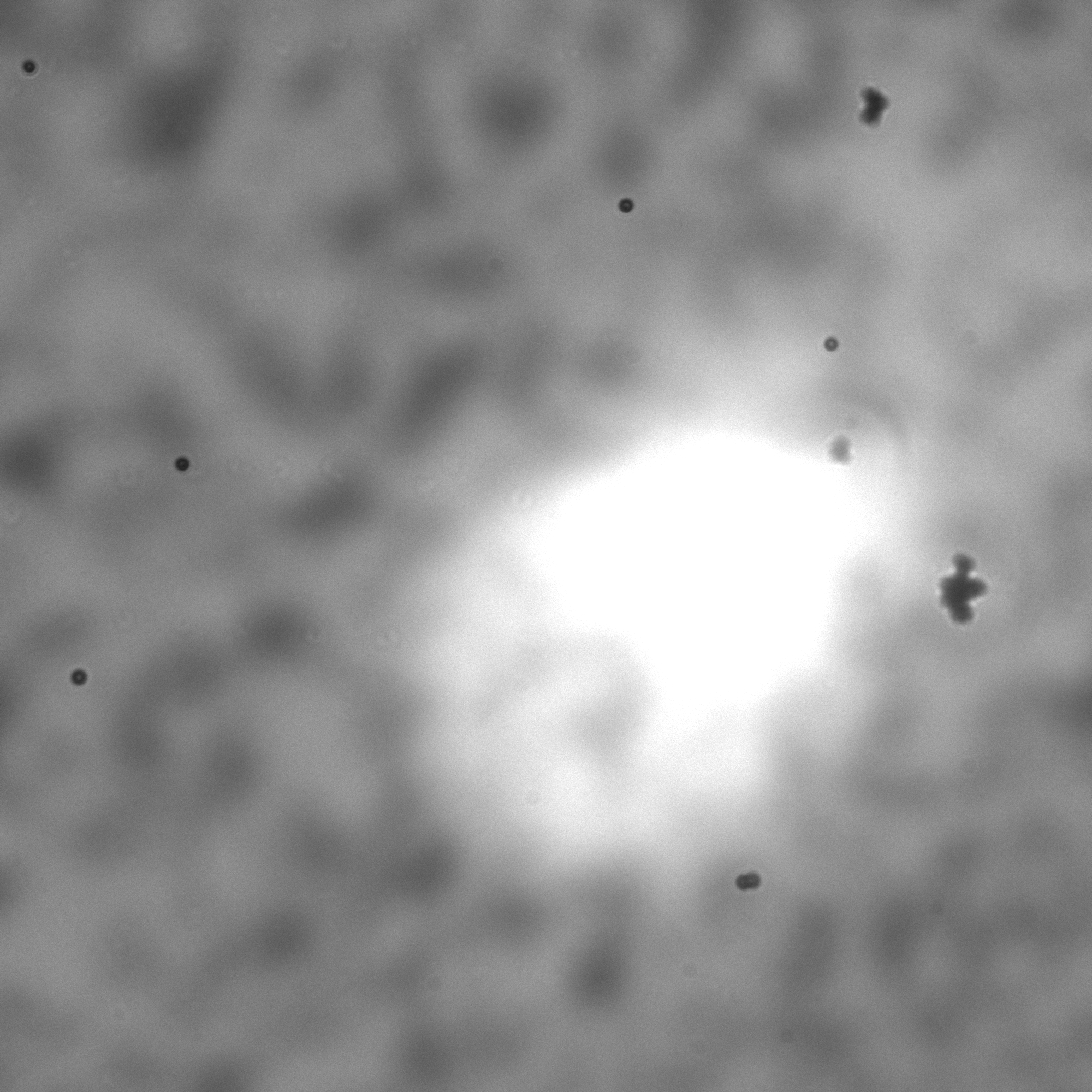

Supplement: Supplementary file 4 — Supplementary Software [file 41467_2023_36373_MOESM4_ESM.zip › analysis software and sample data/CT - Trial Analysis - Sample/49.tiff]

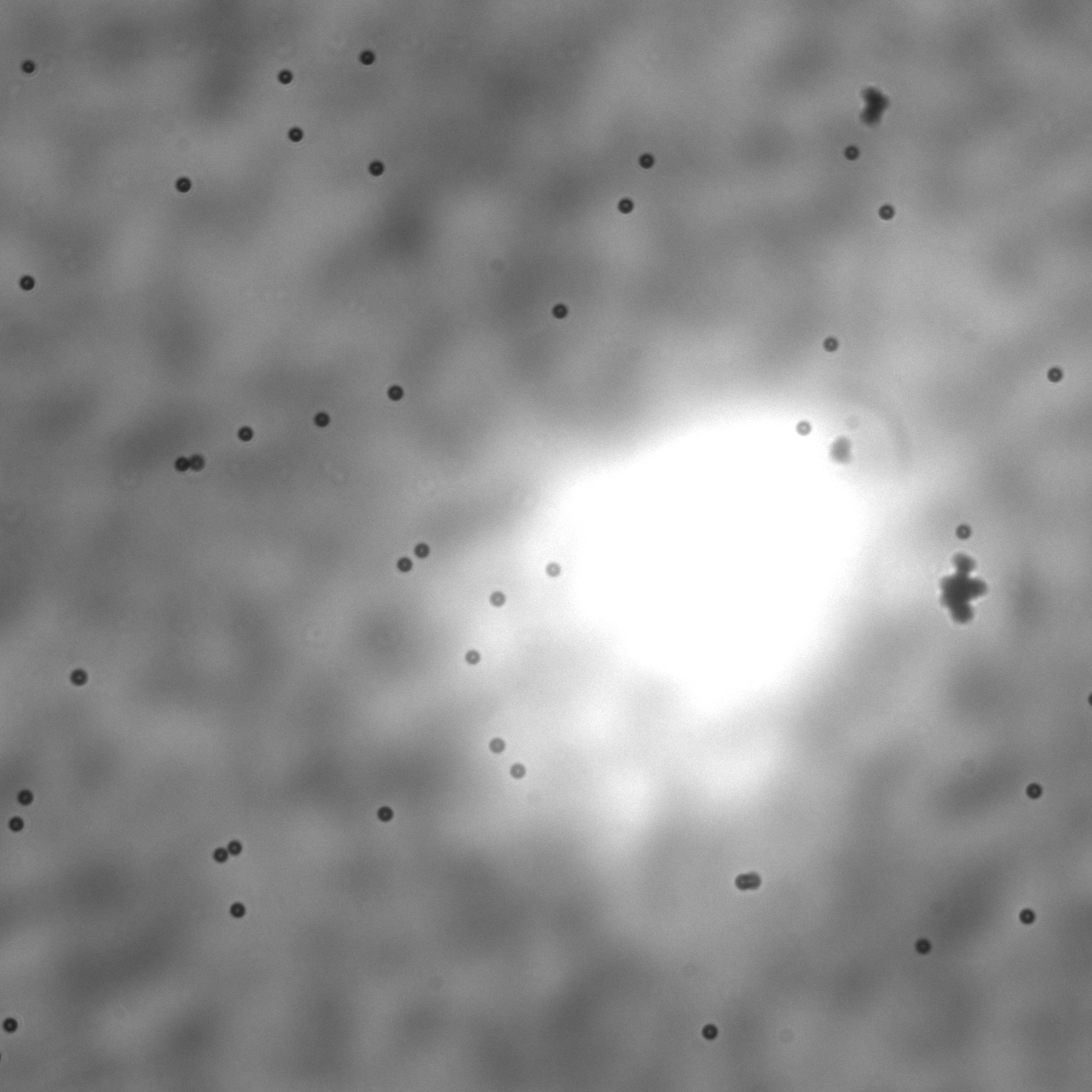

Supplement: Supplementary file 4 — Supplementary Software [file 41467_2023_36373_MOESM4_ESM.zip › analysis software and sample data/CT - Trial Analysis - Sample/5.tiff]

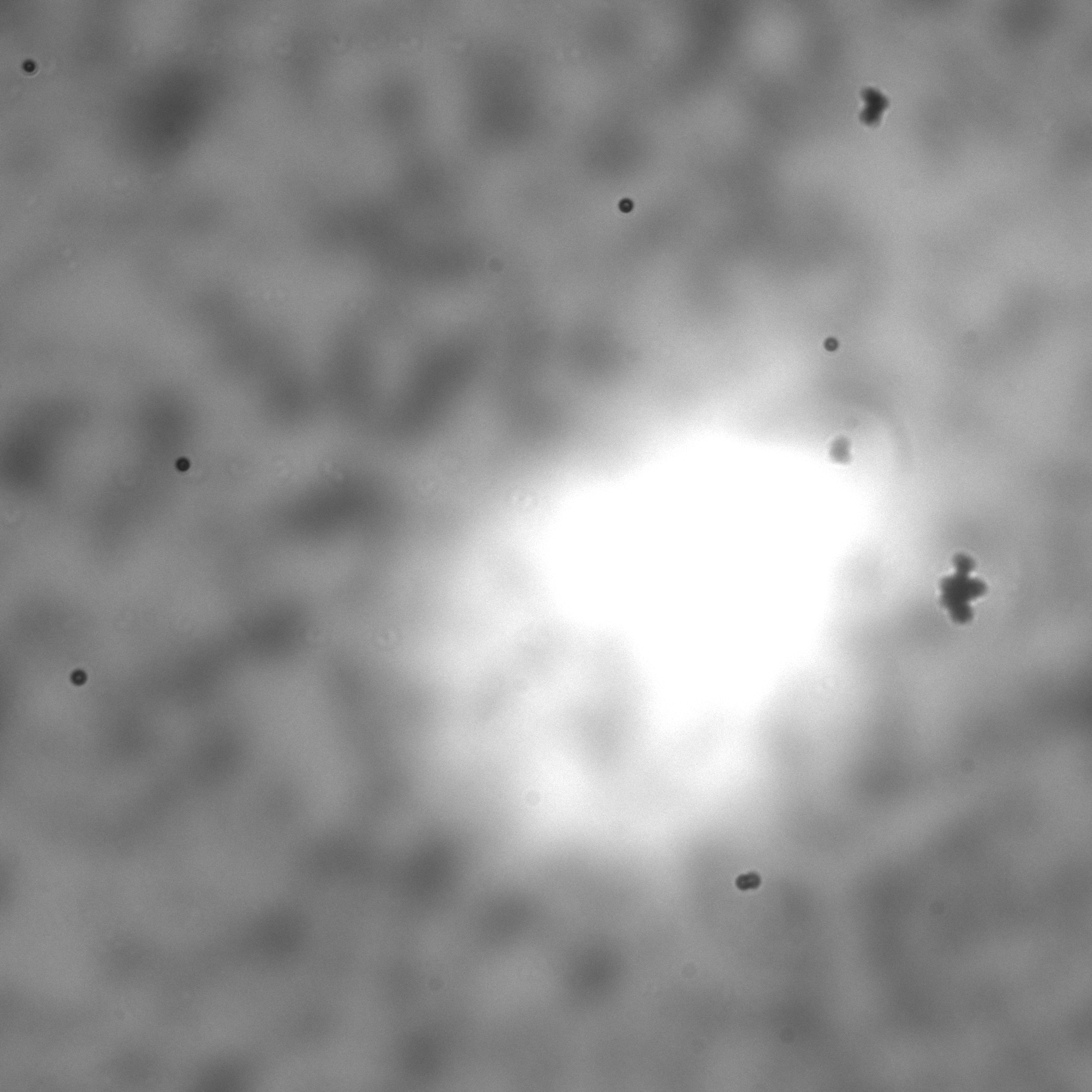

Supplement: Supplementary file 4 — Supplementary Software [file 41467_2023_36373_MOESM4_ESM.zip › analysis software and sample data/CT - Trial Analysis - Sample/50.tiff]

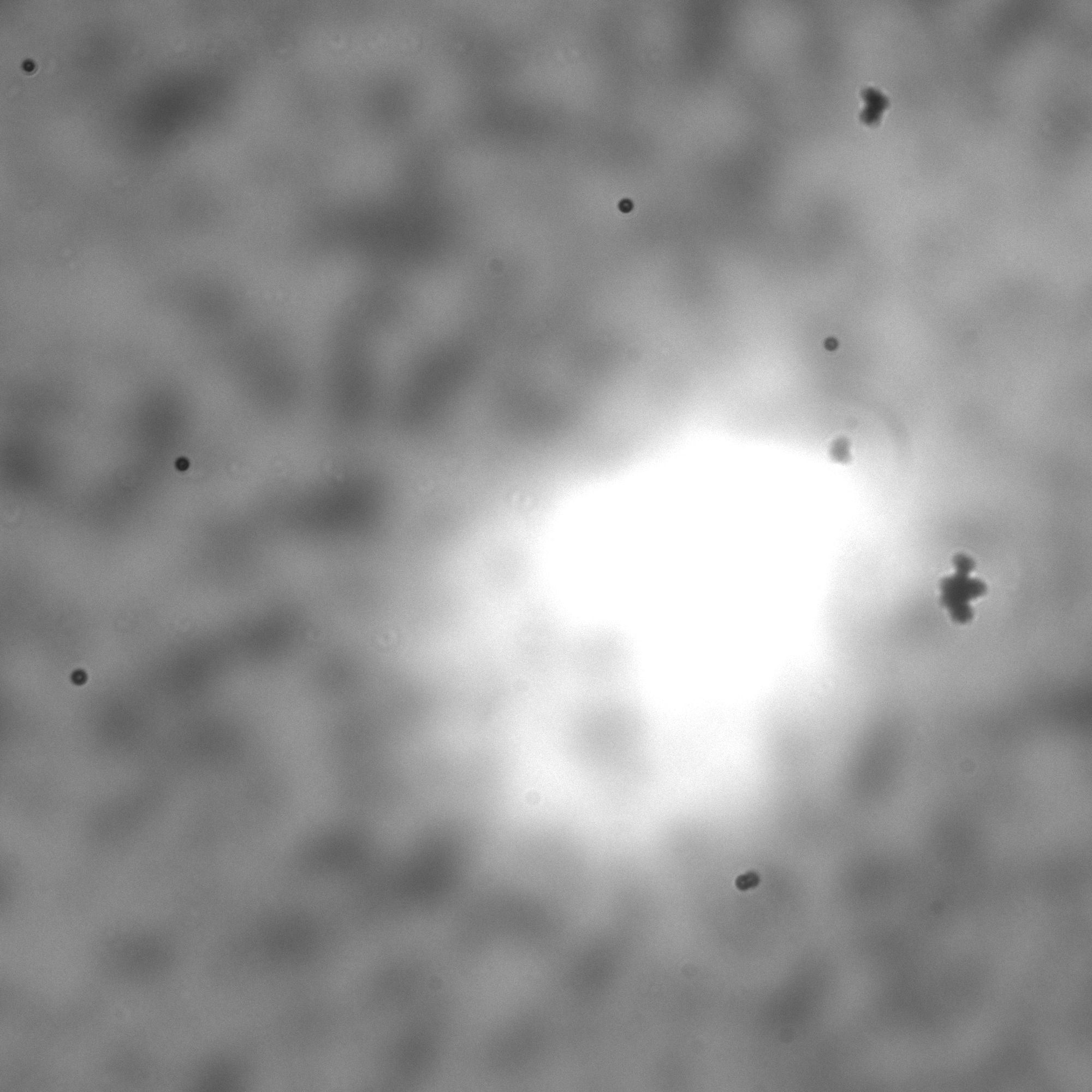

Supplement: Supplementary file 4 — Supplementary Software [file 41467_2023_36373_MOESM4_ESM.zip › analysis software and sample data/CT - Trial Analysis - Sample/51.tiff]

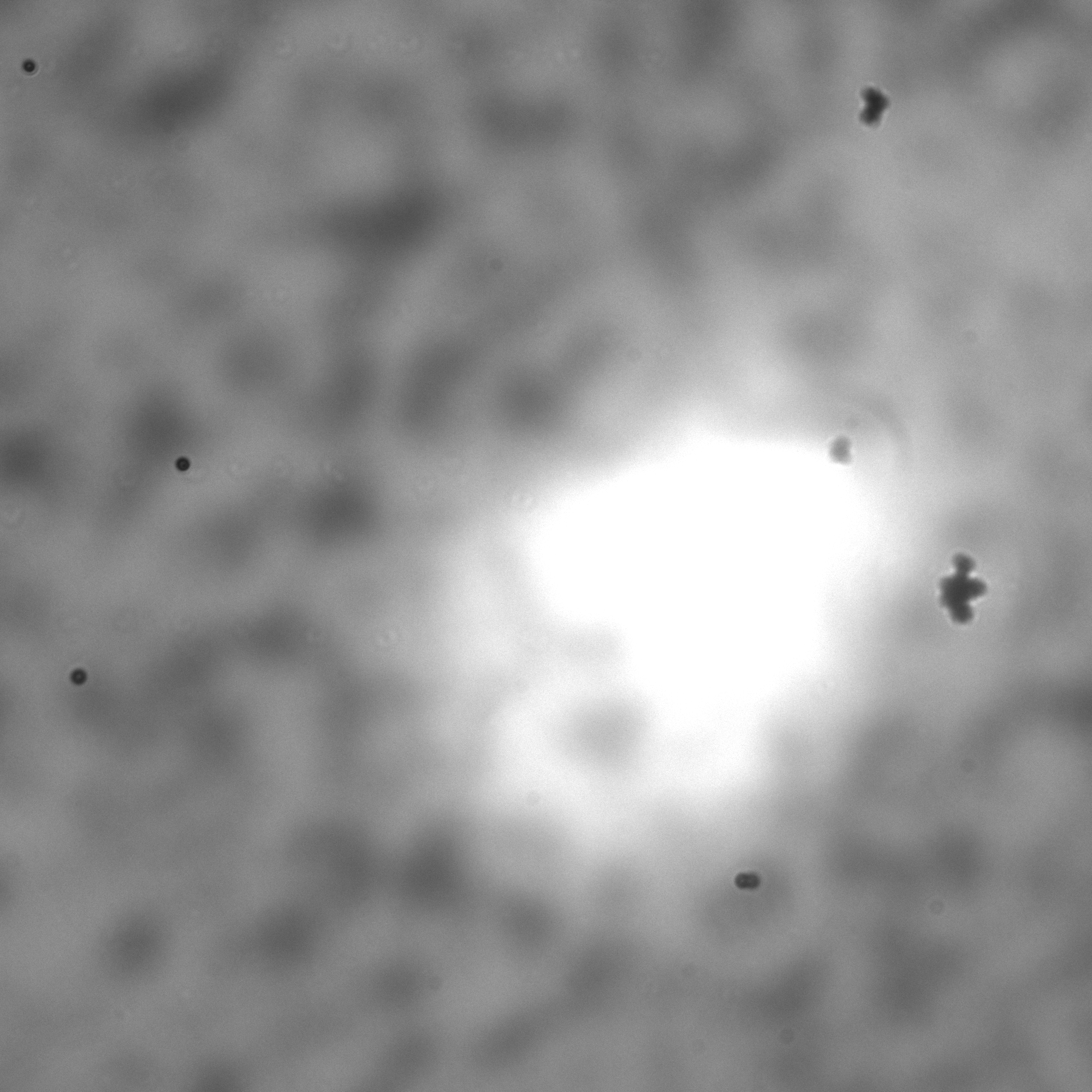

Supplement: Supplementary file 4 — Supplementary Software [file 41467_2023_36373_MOESM4_ESM.zip › analysis software and sample data/CT - Trial Analysis - Sample/52.tiff]

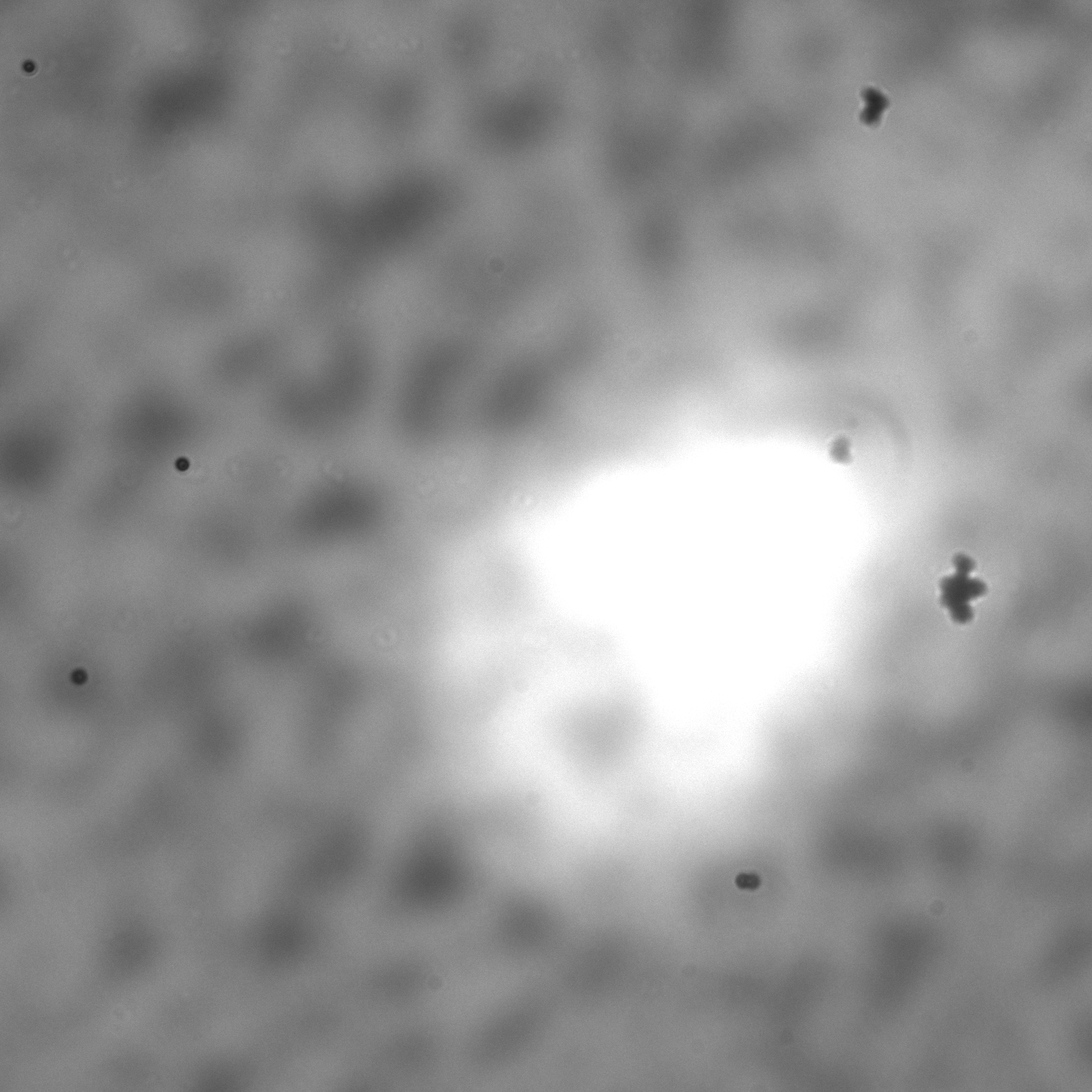

Supplement: Supplementary file 4 — Supplementary Software [file 41467_2023_36373_MOESM4_ESM.zip › analysis software and sample data/CT - Trial Analysis - Sample/53.tiff]

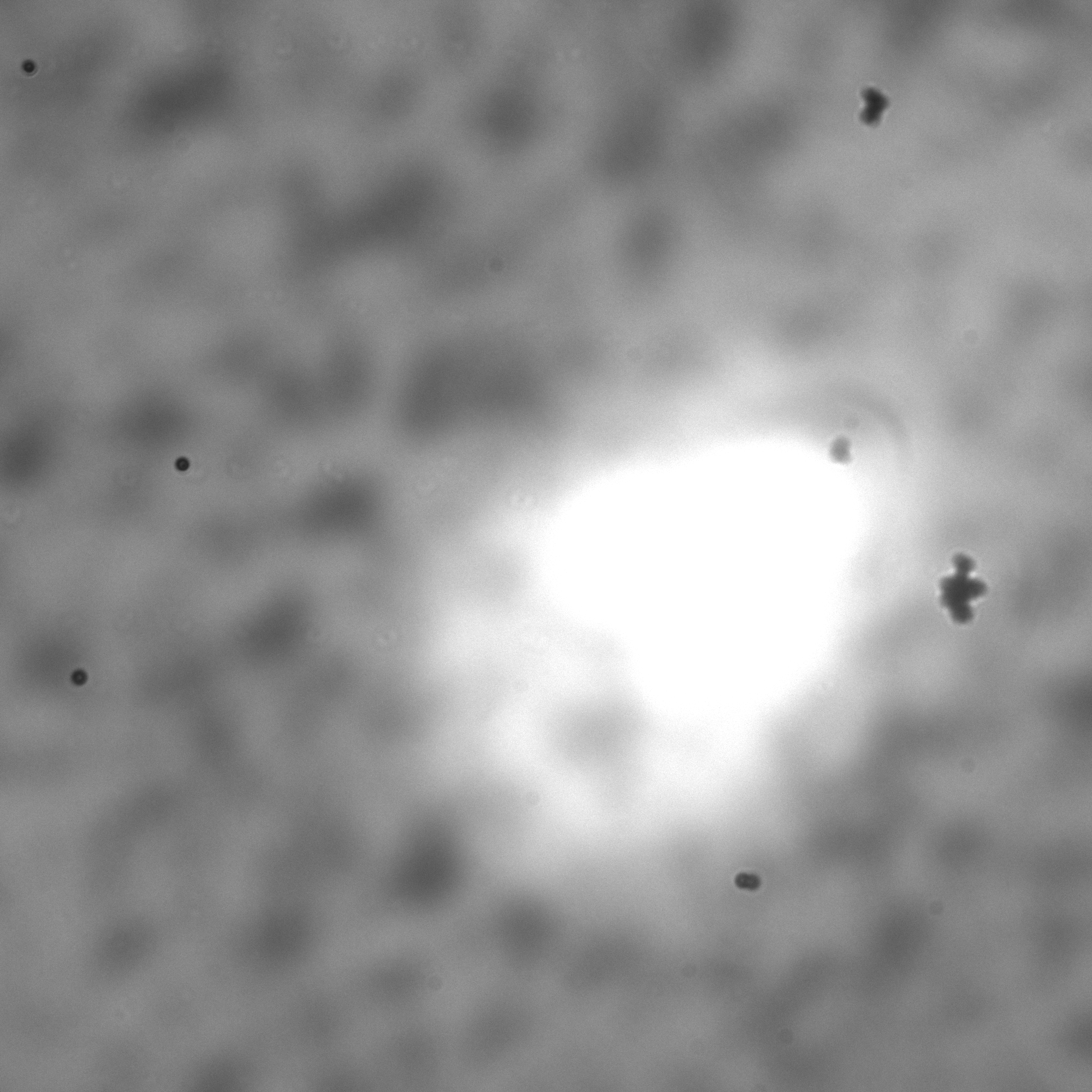

Supplement: Supplementary file 4 — Supplementary Software [file 41467_2023_36373_MOESM4_ESM.zip › analysis software and sample data/CT - Trial Analysis - Sample/54.tiff]

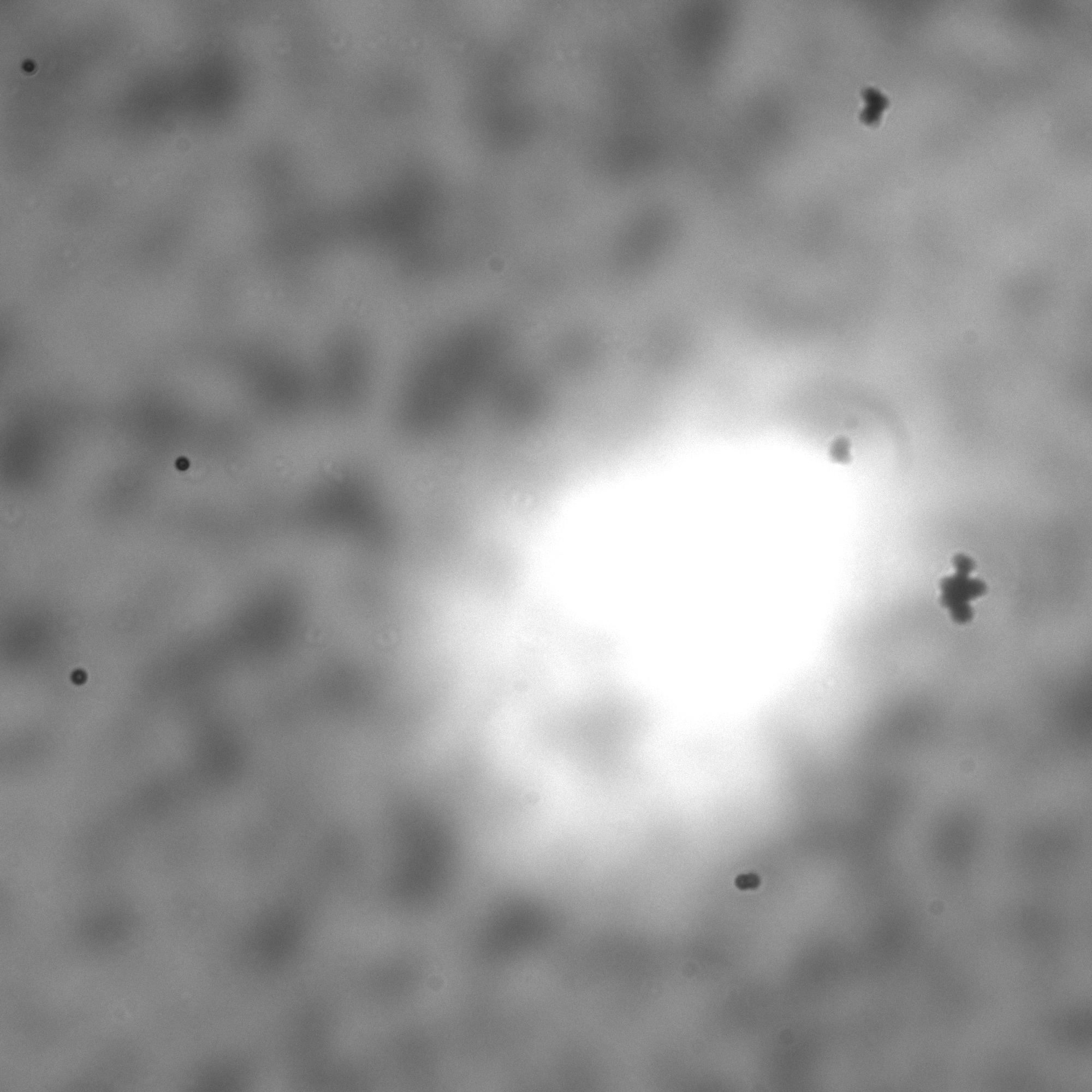

Supplement: Supplementary file 4 — Supplementary Software [file 41467_2023_36373_MOESM4_ESM.zip › analysis software and sample data/CT - Trial Analysis - Sample/55.tiff]

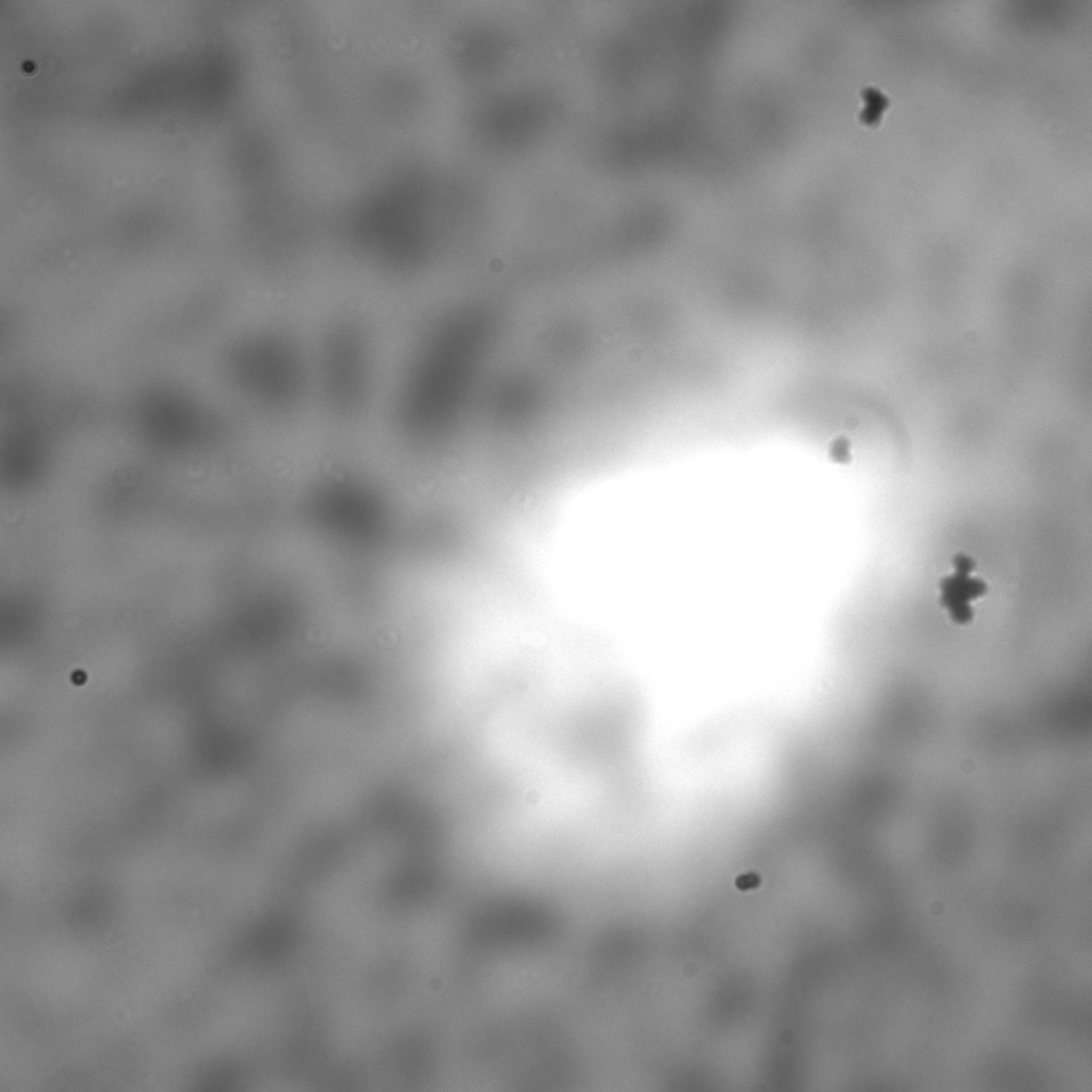

Supplement: Supplementary file 4 — Supplementary Software [file 41467_2023_36373_MOESM4_ESM.zip › analysis software and sample data/CT - Trial Analysis - Sample/56.tiff]

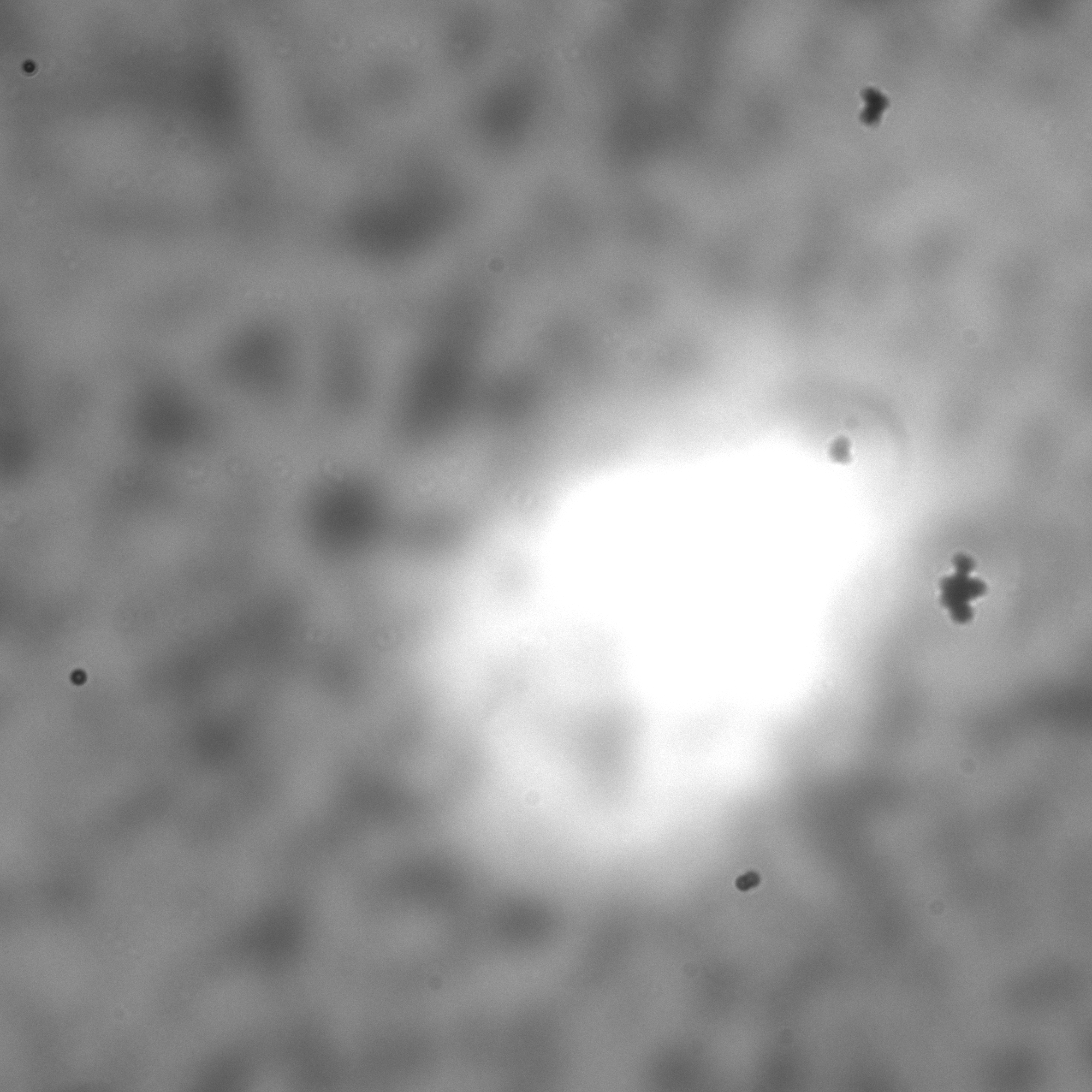

Supplement: Supplementary file 4 — Supplementary Software [file 41467_2023_36373_MOESM4_ESM.zip › analysis software and sample data/CT - Trial Analysis - Sample/57.tiff]

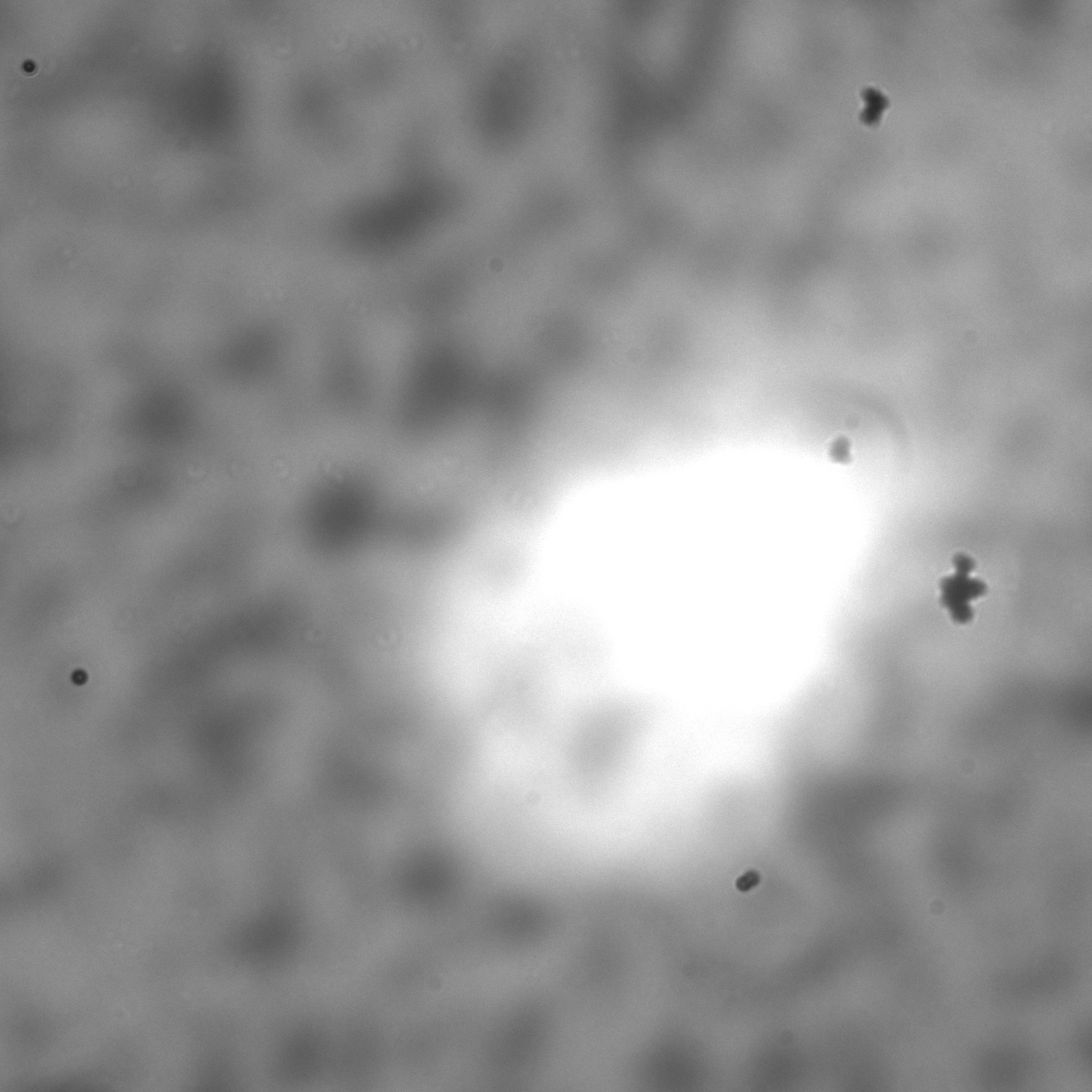

Supplement: Supplementary file 4 — Supplementary Software [file 41467_2023_36373_MOESM4_ESM.zip › analysis software and sample data/CT - Trial Analysis - Sample/58.tiff]

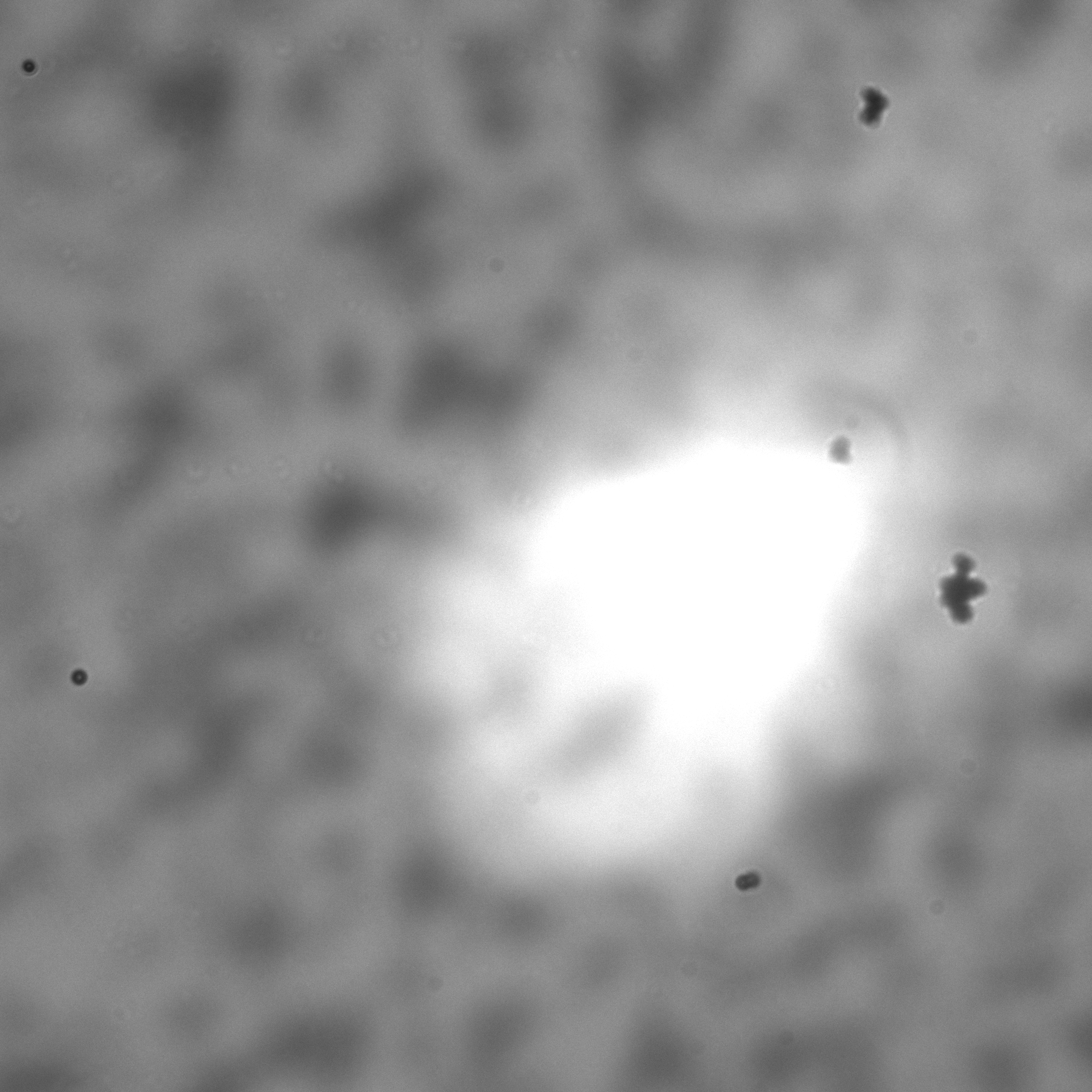

Supplement: Supplementary file 4 — Supplementary Software [file 41467_2023_36373_MOESM4_ESM.zip › analysis software and sample data/CT - Trial Analysis - Sample/59.tiff]

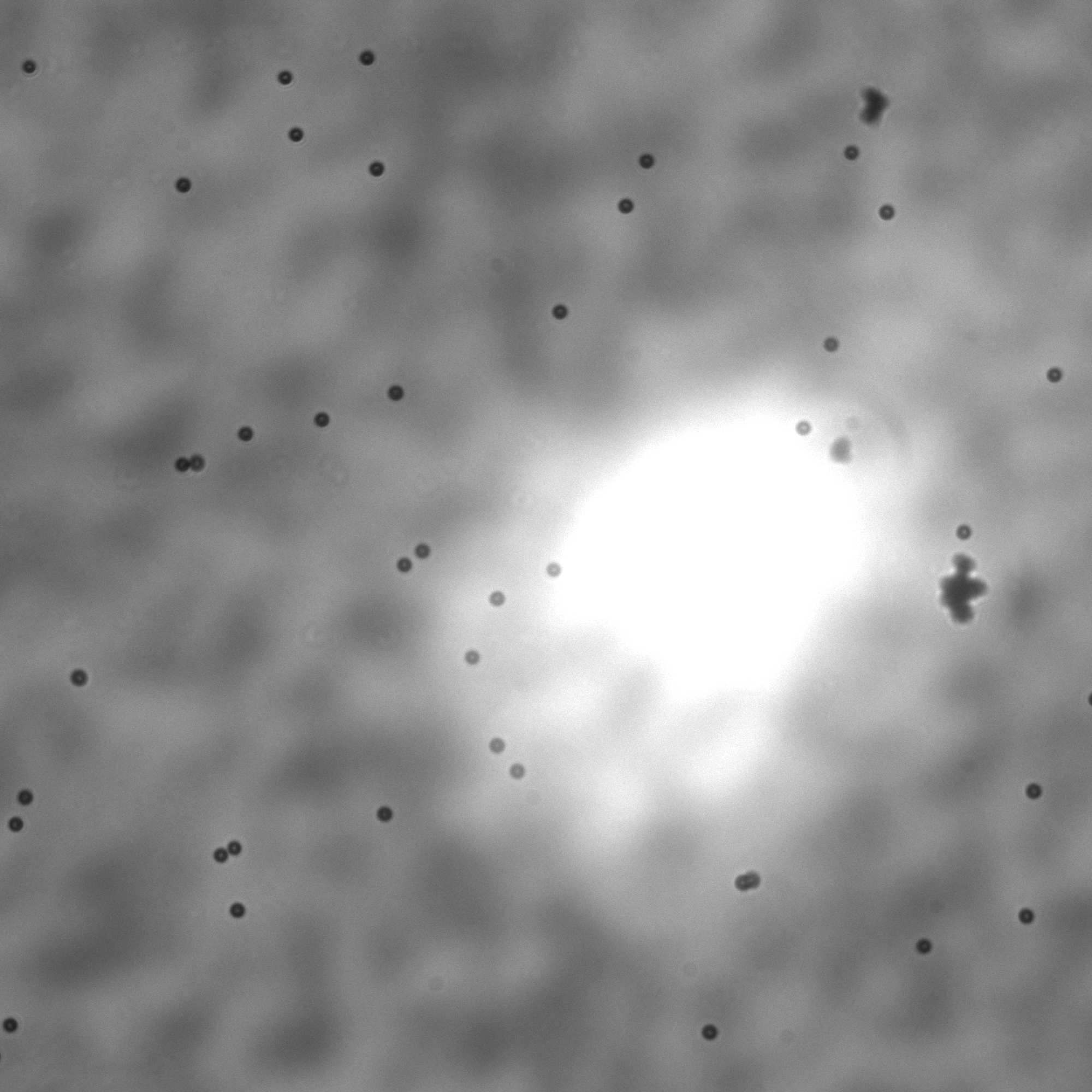

Supplement: Supplementary file 4 — Supplementary Software [file 41467_2023_36373_MOESM4_ESM.zip › analysis software and sample data/CT - Trial Analysis - Sample/6.tiff]

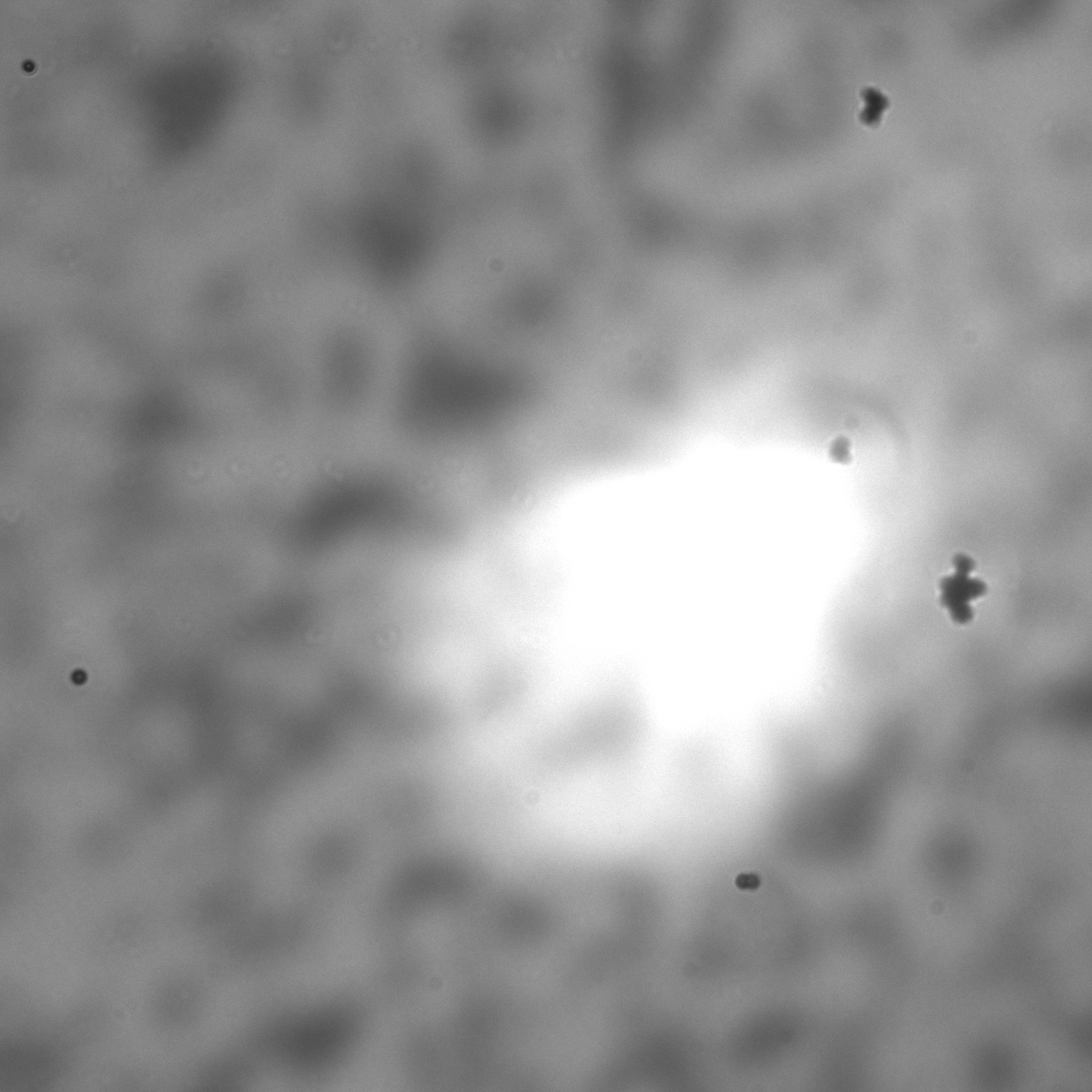

Supplement: Supplementary file 4 — Supplementary Software [file 41467_2023_36373_MOESM4_ESM.zip › analysis software and sample data/CT - Trial Analysis - Sample/60.tiff]

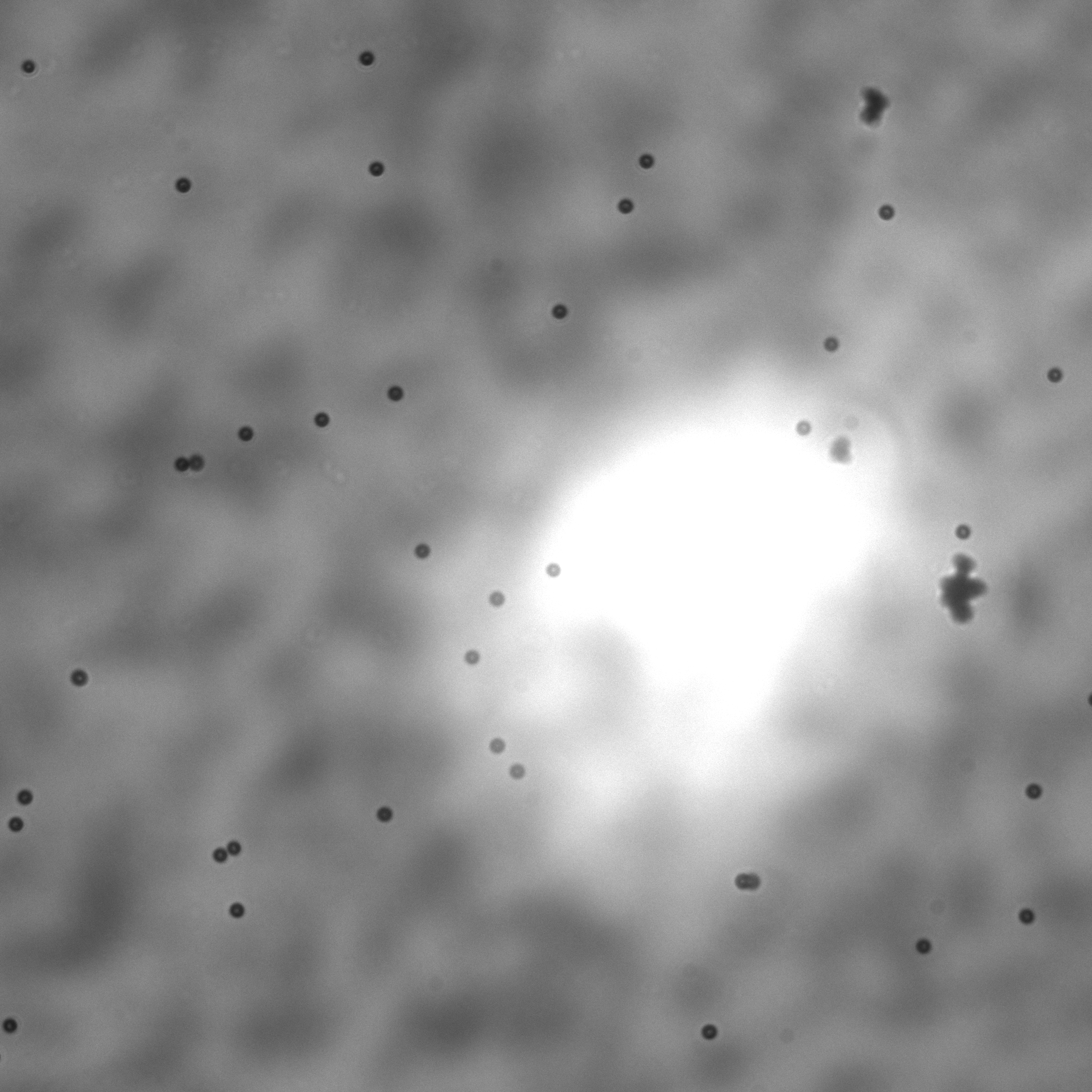

Supplement: Supplementary file 4 — Supplementary Software [file 41467_2023_36373_MOESM4_ESM.zip › analysis software and sample data/CT - Trial Analysis - Sample/7.tiff]

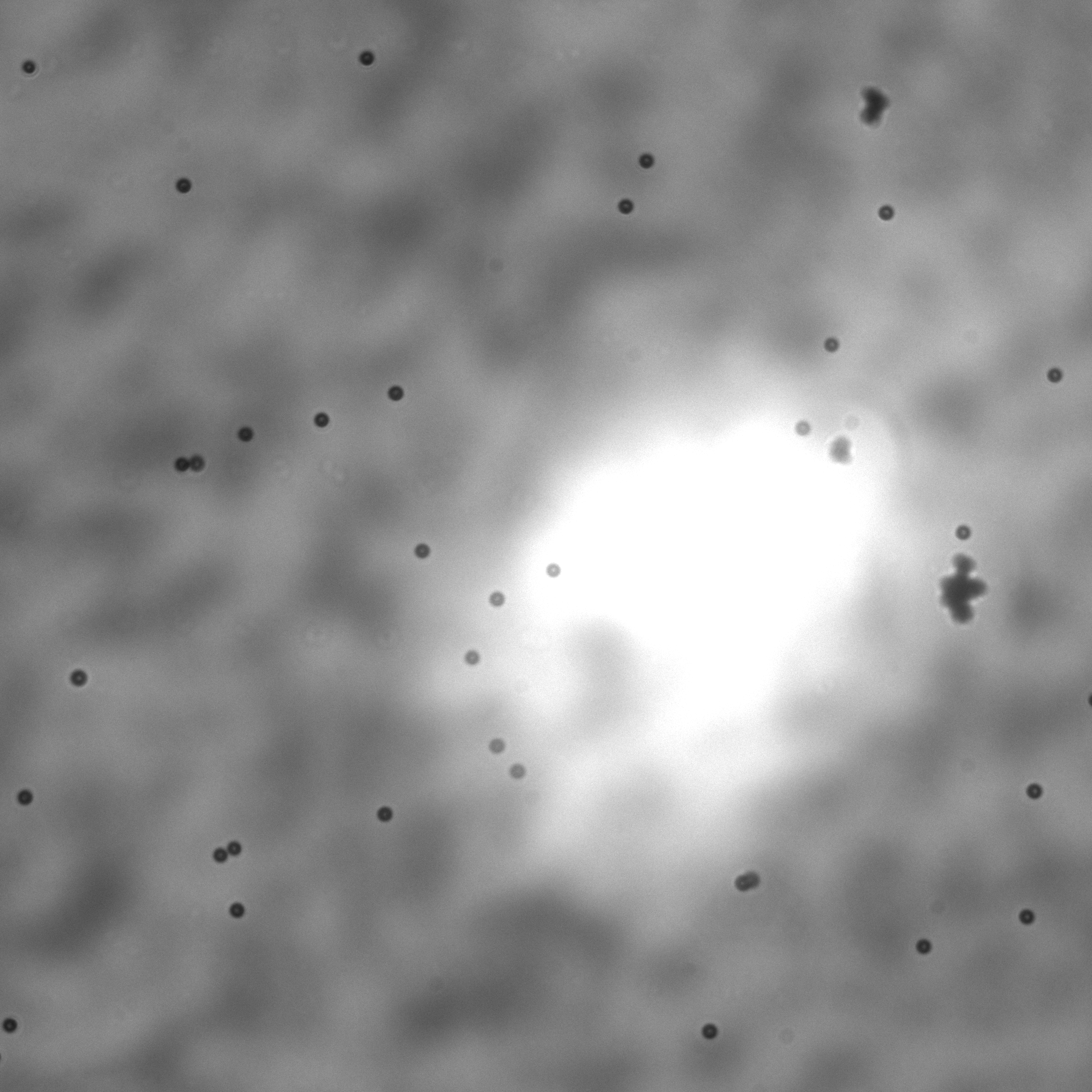

Supplement: Supplementary file 4 — Supplementary Software [file 41467_2023_36373_MOESM4_ESM.zip › analysis software and sample data/CT - Trial Analysis - Sample/8.tiff]

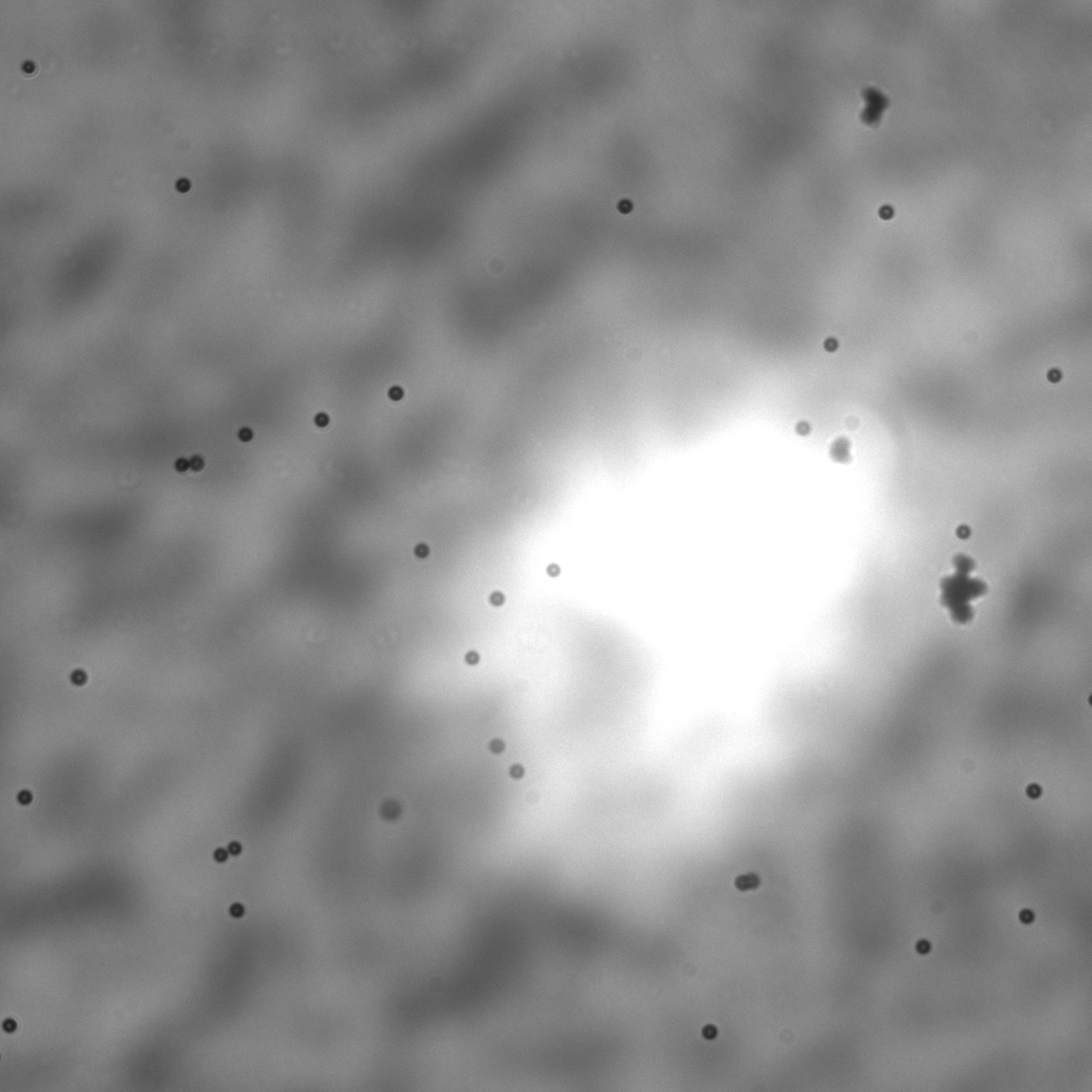

Supplement: Supplementary file 4 — Supplementary Software [file 41467_2023_36373_MOESM4_ESM.zip › analysis software and sample data/CT - Trial Analysis - Sample/9.tiff]

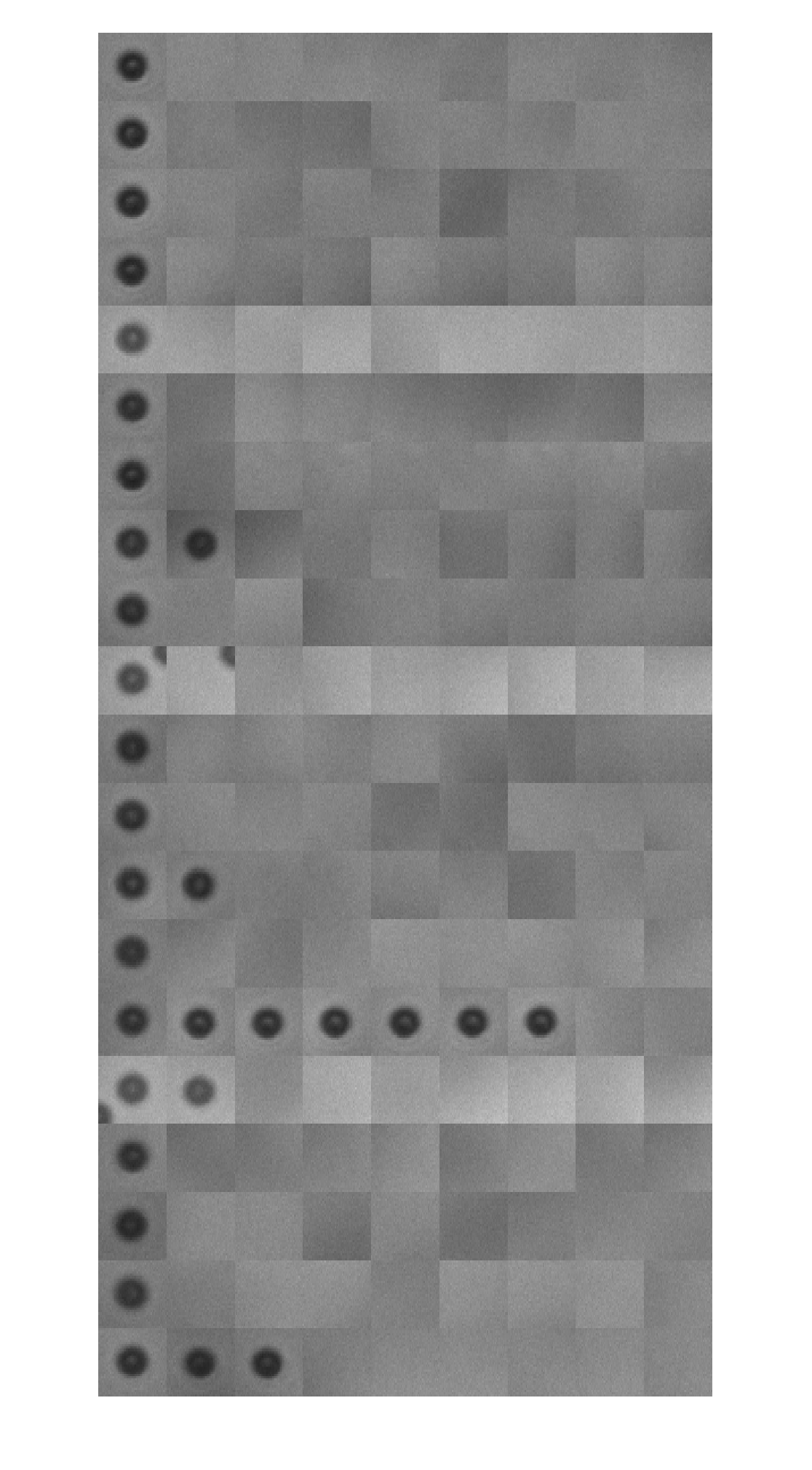

Supplement: Supplementary file 4 — Supplementary Software [file 41467_2023_36373_MOESM4_ESM.zip › analysis software and sample data/CT - Trial Analysis - Sample/Beads from 1 to 20.tif]

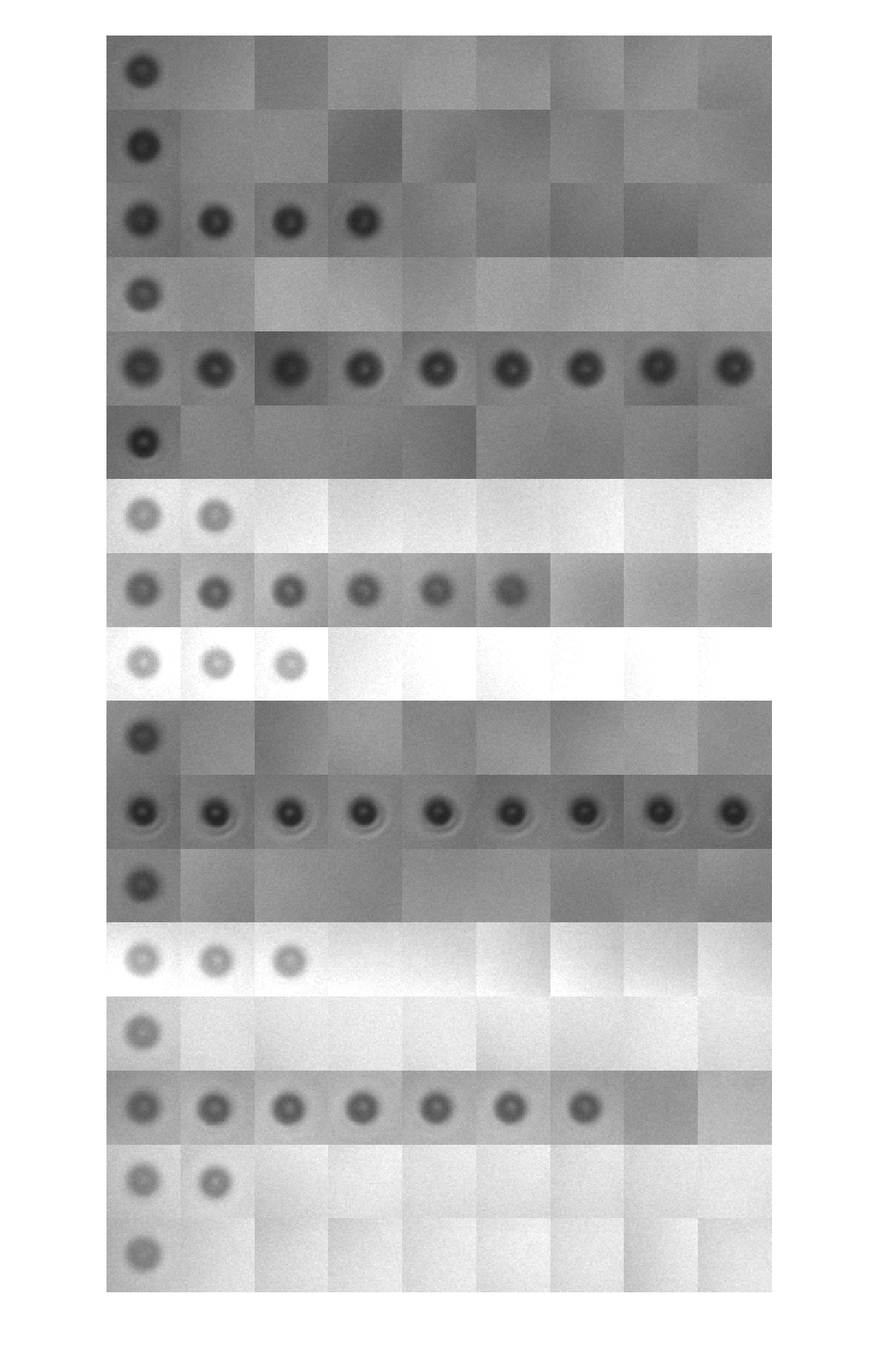

Supplement: Supplementary file 4 — Supplementary Software [file 41467_2023_36373_MOESM4_ESM.zip › analysis software and sample data/CT - Trial Analysis - Sample/Beads from 21 to 37.tif]

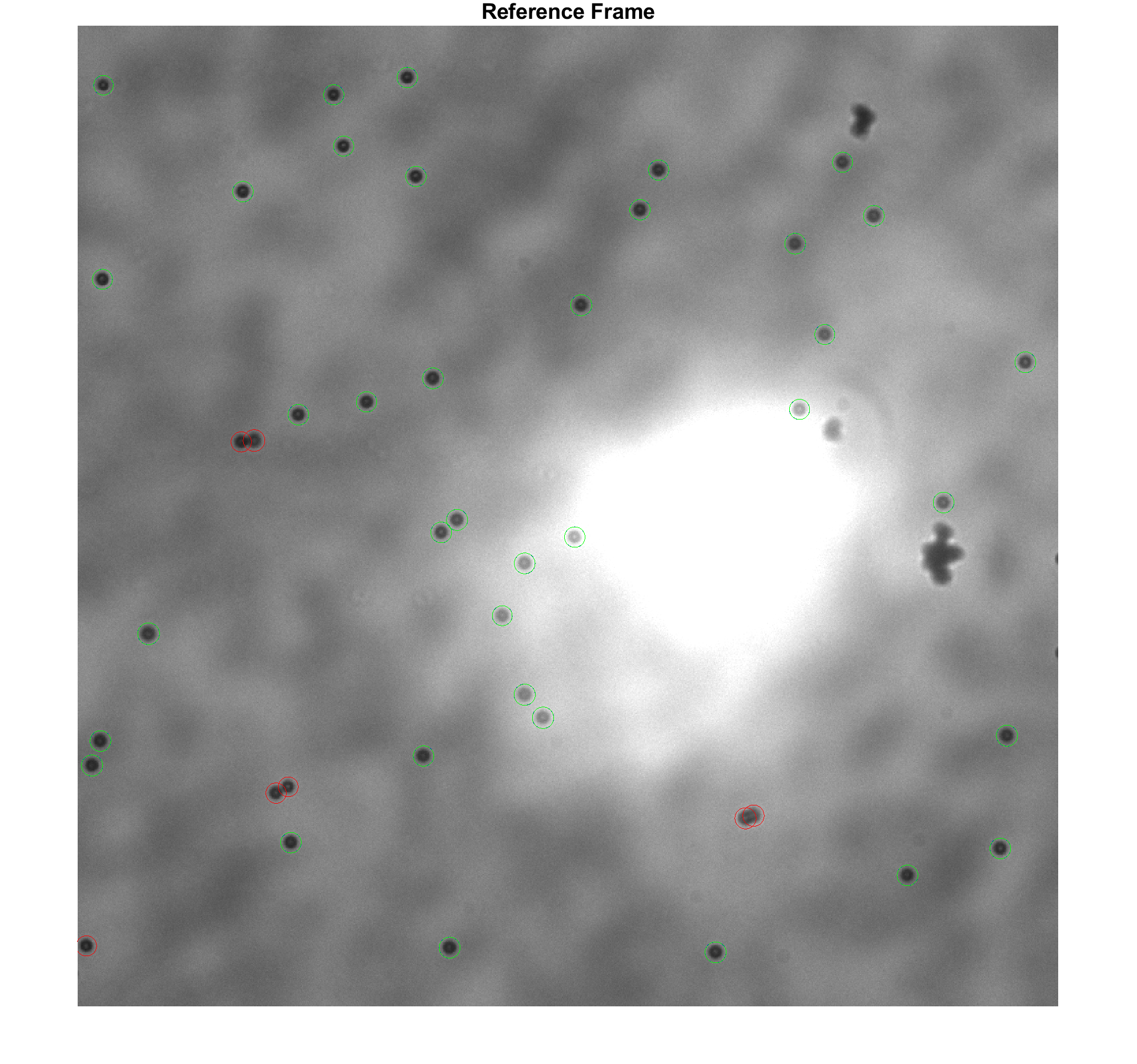

Supplement: Supplementary file 4 — Supplementary Software [file 41467_2023_36373_MOESM4_ESM.zip › analysis software and sample data/CT - Trial Analysis - Sample/Refrence Frame.tif]

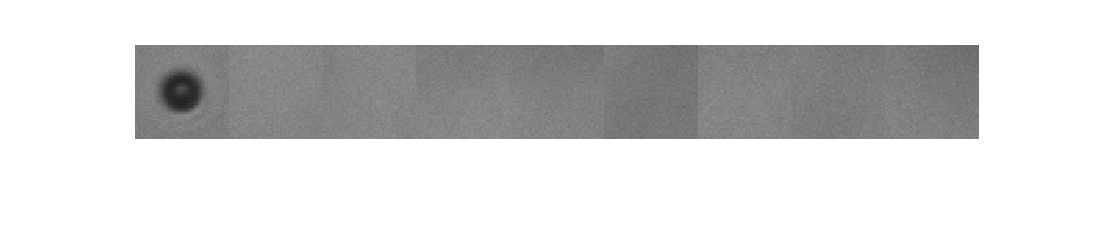

Supplement: Supplementary file 4 — Supplementary Software [file 41467_2023_36373_MOESM4_ESM.zip › analysis software and sample data/CT - Trial Analysis - Sample/Single tethered Bead # 1.tif]

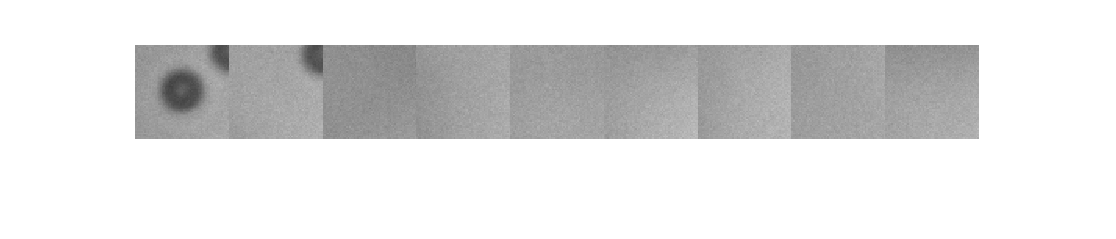

Supplement: Supplementary file 4 — Supplementary Software [file 41467_2023_36373_MOESM4_ESM.zip › analysis software and sample data/CT - Trial Analysis - Sample/Single tethered Bead # 10.tif]

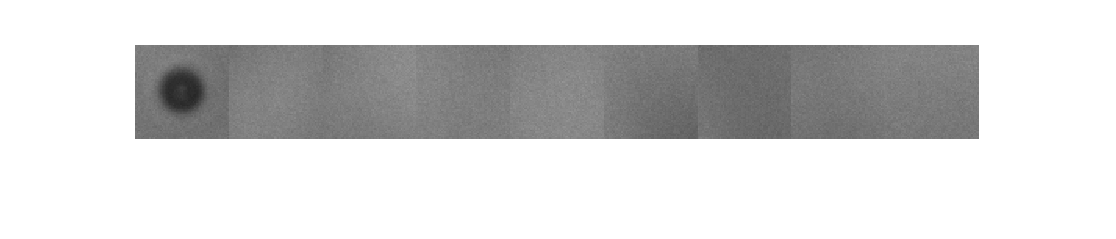

Supplement: Supplementary file 4 — Supplementary Software [file 41467_2023_36373_MOESM4_ESM.zip › analysis software and sample data/CT - Trial Analysis - Sample/Single tethered Bead # 11.tif]

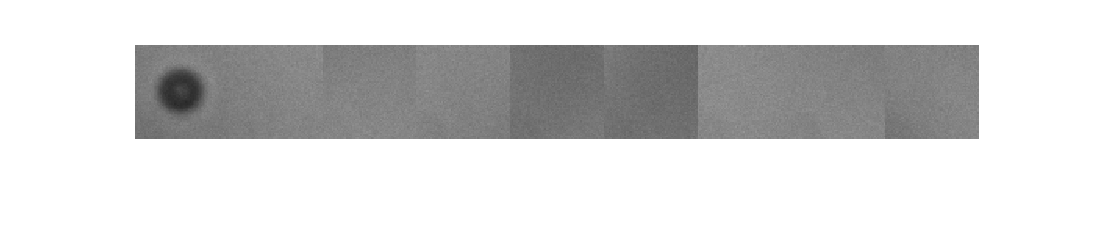

Supplement: Supplementary file 4 — Supplementary Software [file 41467_2023_36373_MOESM4_ESM.zip › analysis software and sample data/CT - Trial Analysis - Sample/Single tethered Bead # 12.tif]

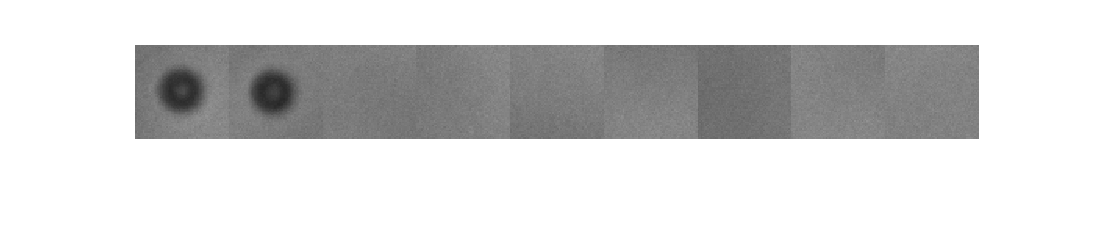

Supplement: Supplementary file 4 — Supplementary Software [file 41467_2023_36373_MOESM4_ESM.zip › analysis software and sample data/CT - Trial Analysis - Sample/Single tethered Bead # 13.tif]

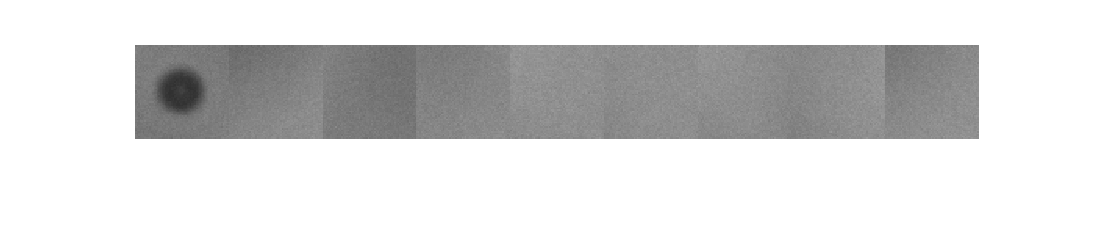

Supplement: Supplementary file 4 — Supplementary Software [file 41467_2023_36373_MOESM4_ESM.zip › analysis software and sample data/CT - Trial Analysis - Sample/Single tethered Bead # 14.tif]

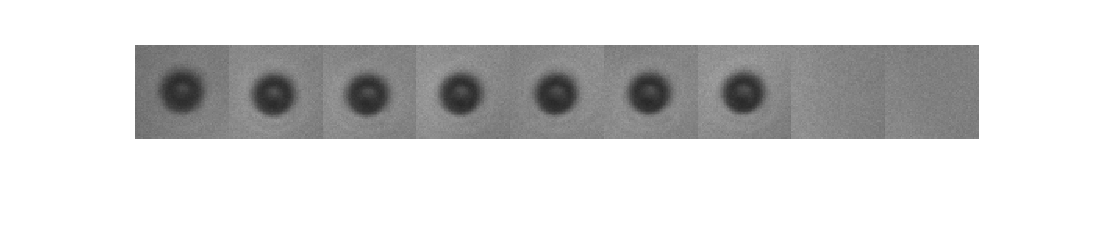

Supplement: Supplementary file 4 — Supplementary Software [file 41467_2023_36373_MOESM4_ESM.zip › analysis software and sample data/CT - Trial Analysis - Sample/Single tethered Bead # 15.tif]

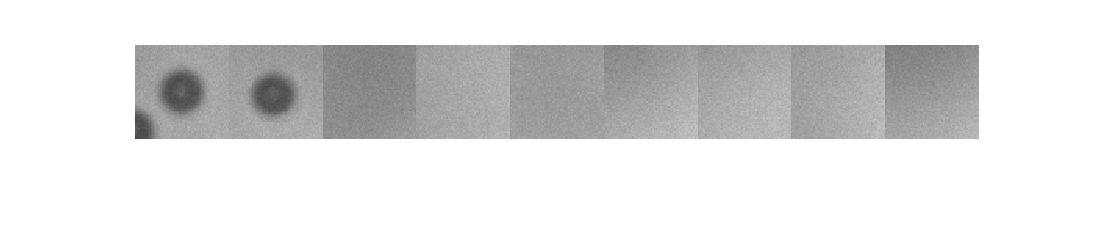

Supplement: Supplementary file 4 — Supplementary Software [file 41467_2023_36373_MOESM4_ESM.zip › analysis software and sample data/CT - Trial Analysis - Sample/Single tethered Bead # 16.tif]

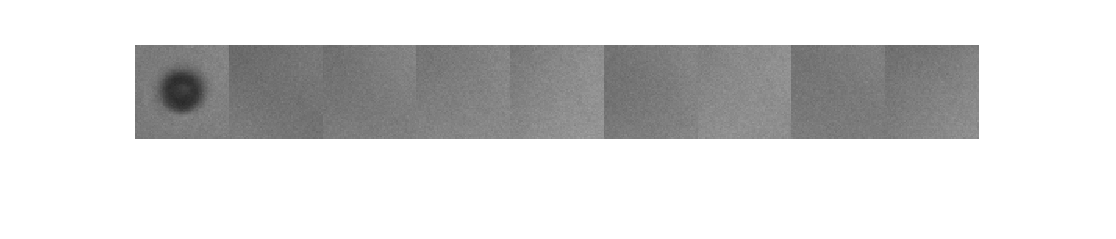

Supplement: Supplementary file 4 — Supplementary Software [file 41467_2023_36373_MOESM4_ESM.zip › analysis software and sample data/CT - Trial Analysis - Sample/Single tethered Bead # 17.tif]

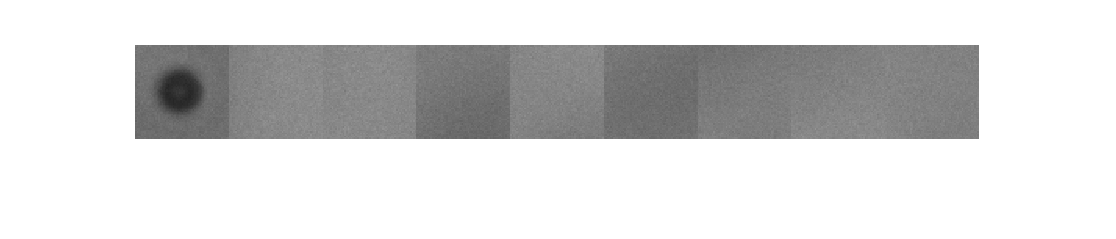

Supplement: Supplementary file 4 — Supplementary Software [file 41467_2023_36373_MOESM4_ESM.zip › analysis software and sample data/CT - Trial Analysis - Sample/Single tethered Bead # 18.tif]

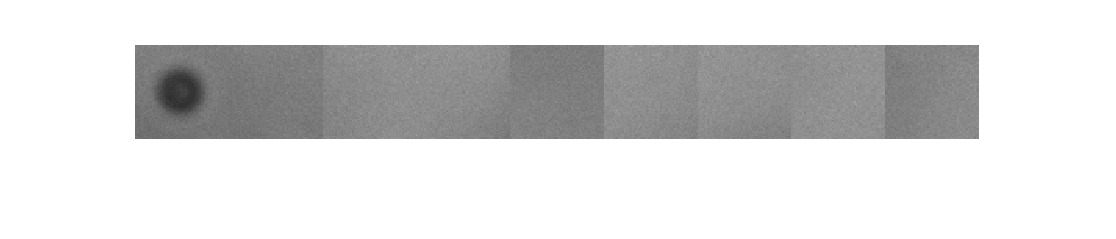

Supplement: Supplementary file 4 — Supplementary Software [file 41467_2023_36373_MOESM4_ESM.zip › analysis software and sample data/CT - Trial Analysis - Sample/Single tethered Bead # 19.tif]

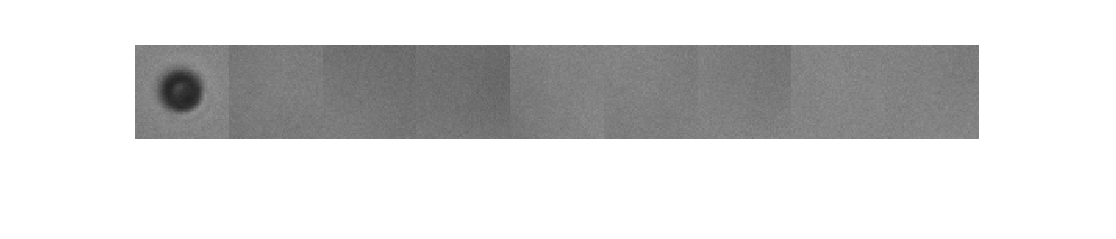

Supplement: Supplementary file 4 — Supplementary Software [file 41467_2023_36373_MOESM4_ESM.zip › analysis software and sample data/CT - Trial Analysis - Sample/Single tethered Bead # 2.tif]

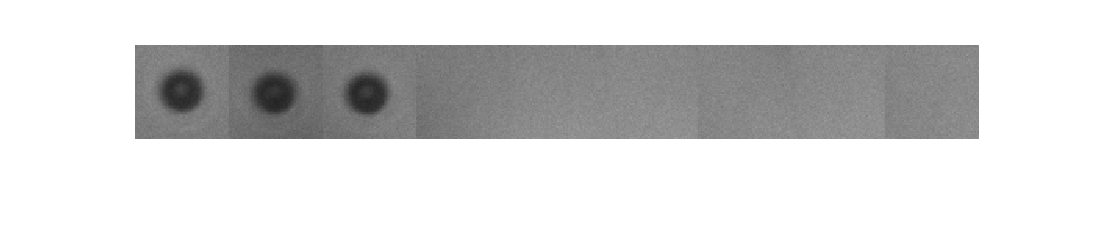

Supplement: Supplementary file 4 — Supplementary Software [file 41467_2023_36373_MOESM4_ESM.zip › analysis software and sample data/CT - Trial Analysis - Sample/Single tethered Bead # 20.tif]

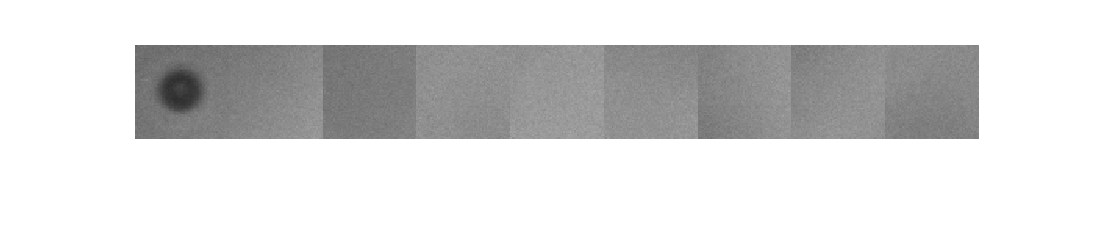

Supplement: Supplementary file 4 — Supplementary Software [file 41467_2023_36373_MOESM4_ESM.zip › analysis software and sample data/CT - Trial Analysis - Sample/Single tethered Bead # 21.tif]

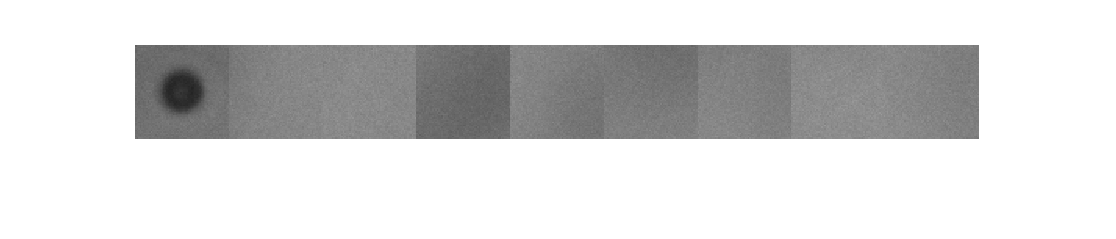

Supplement: Supplementary file 4 — Supplementary Software [file 41467_2023_36373_MOESM4_ESM.zip › analysis software and sample data/CT - Trial Analysis - Sample/Single tethered Bead # 22.tif]

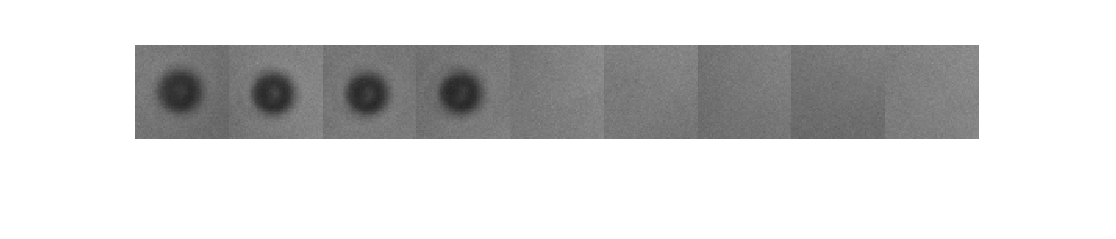

Supplement: Supplementary file 4 — Supplementary Software [file 41467_2023_36373_MOESM4_ESM.zip › analysis software and sample data/CT - Trial Analysis - Sample/Single tethered Bead # 23.tif]

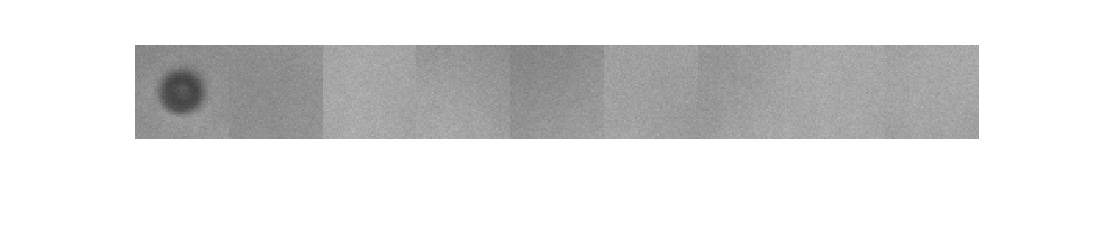

Supplement: Supplementary file 4 — Supplementary Software [file 41467_2023_36373_MOESM4_ESM.zip › analysis software and sample data/CT - Trial Analysis - Sample/Single tethered Bead # 24.tif]

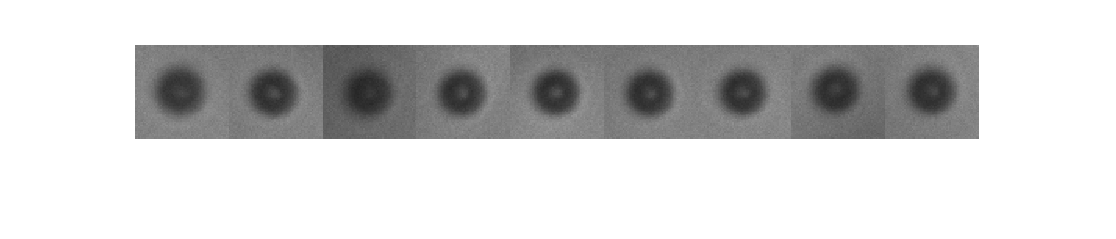

Supplement: Supplementary file 4 — Supplementary Software [file 41467_2023_36373_MOESM4_ESM.zip › analysis software and sample data/CT - Trial Analysis - Sample/Single tethered Bead # 25.tif]

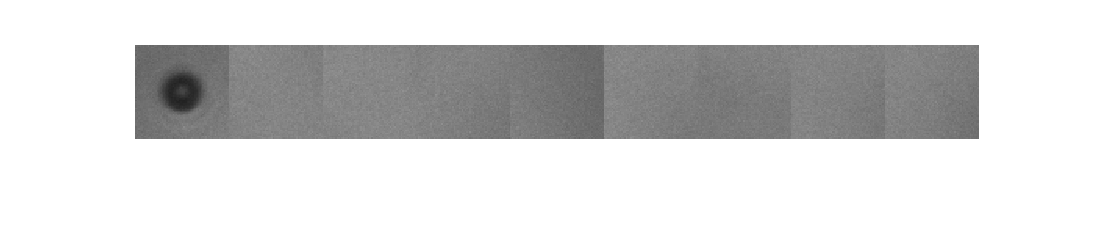

Supplement: Supplementary file 4 — Supplementary Software [file 41467_2023_36373_MOESM4_ESM.zip › analysis software and sample data/CT - Trial Analysis - Sample/Single tethered Bead # 26.tif]

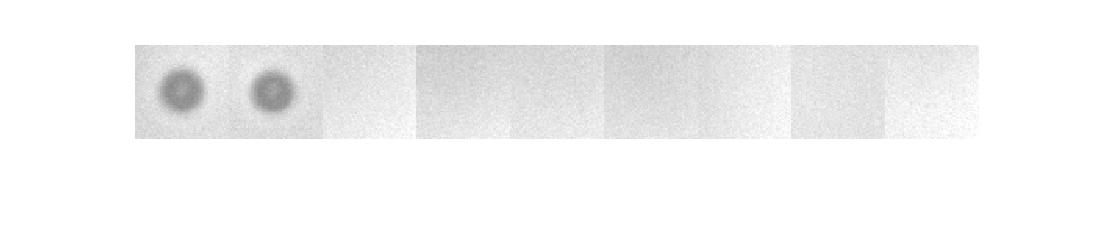

Supplement: Supplementary file 4 — Supplementary Software [file 41467_2023_36373_MOESM4_ESM.zip › analysis software and sample data/CT - Trial Analysis - Sample/Single tethered Bead # 27.tif]

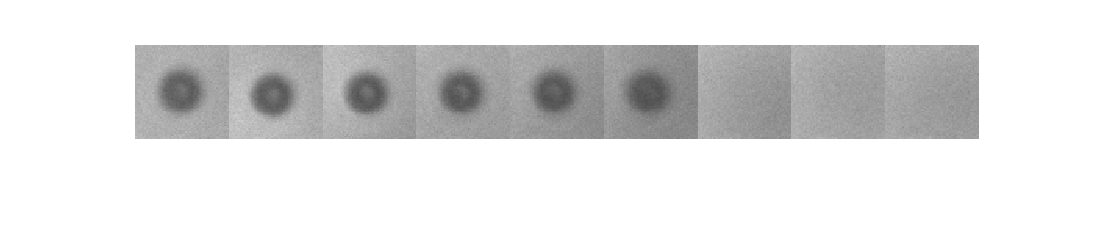

Supplement: Supplementary file 4 — Supplementary Software [file 41467_2023_36373_MOESM4_ESM.zip › analysis software and sample data/CT - Trial Analysis - Sample/Single tethered Bead # 28.tif]

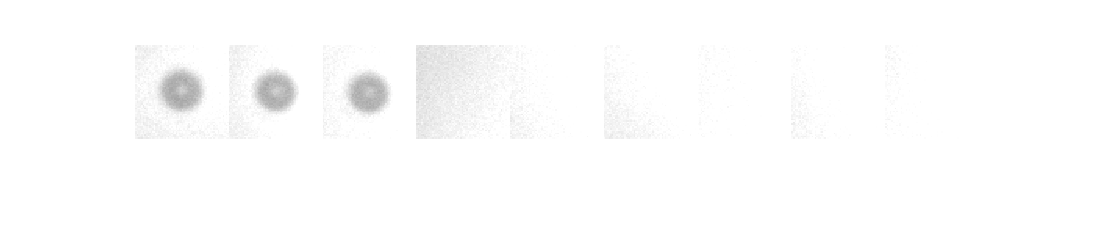

Supplement: Supplementary file 4 — Supplementary Software [file 41467_2023_36373_MOESM4_ESM.zip › analysis software and sample data/CT - Trial Analysis - Sample/Single tethered Bead # 29.tif]

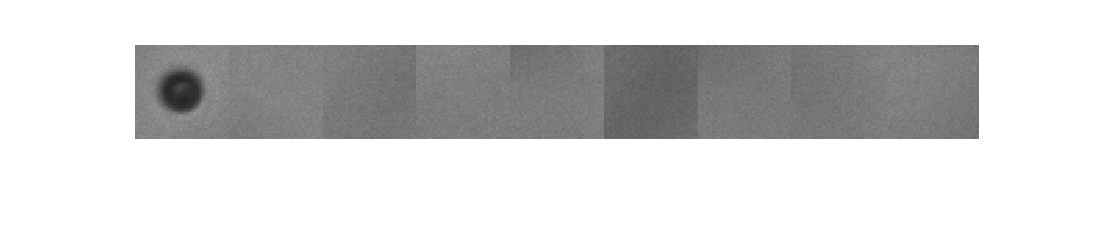

Supplement: Supplementary file 4 — Supplementary Software [file 41467_2023_36373_MOESM4_ESM.zip › analysis software and sample data/CT - Trial Analysis - Sample/Single tethered Bead # 3.tif]

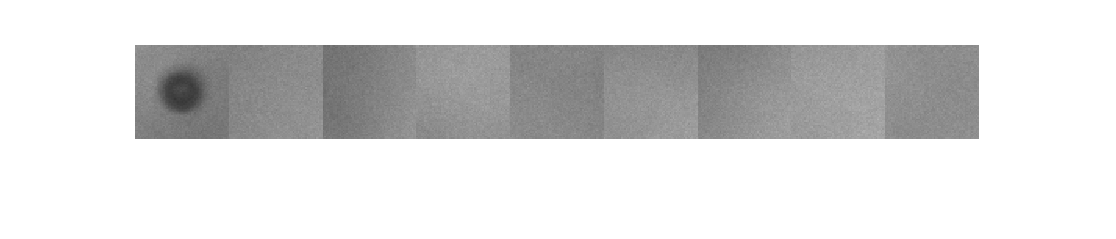

Supplement: Supplementary file 4 — Supplementary Software [file 41467_2023_36373_MOESM4_ESM.zip › analysis software and sample data/CT - Trial Analysis - Sample/Single tethered Bead # 30.tif]

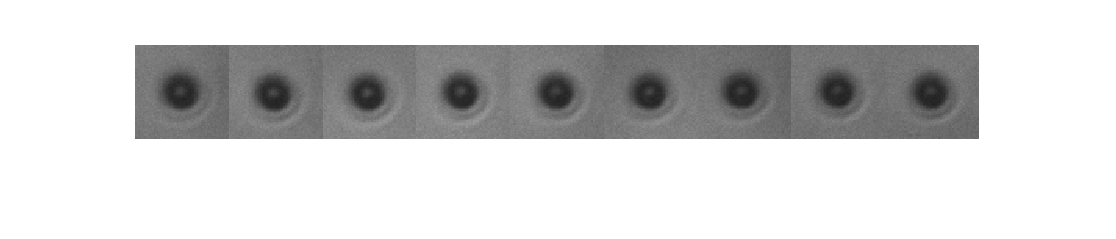

Supplement: Supplementary file 4 — Supplementary Software [file 41467_2023_36373_MOESM4_ESM.zip › analysis software and sample data/CT - Trial Analysis - Sample/Single tethered Bead # 31.tif]

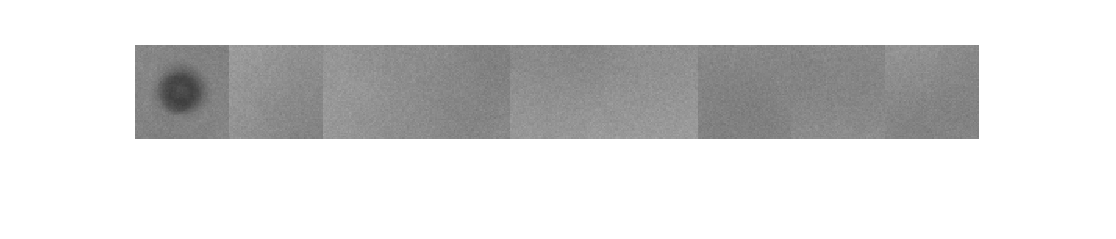

Supplement: Supplementary file 4 — Supplementary Software [file 41467_2023_36373_MOESM4_ESM.zip › analysis software and sample data/CT - Trial Analysis - Sample/Single tethered Bead # 32.tif]

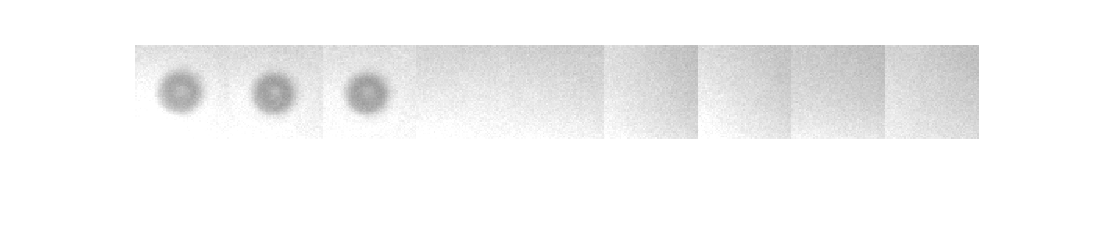

Supplement: Supplementary file 4 — Supplementary Software [file 41467_2023_36373_MOESM4_ESM.zip › analysis software and sample data/CT - Trial Analysis - Sample/Single tethered Bead # 33.tif]

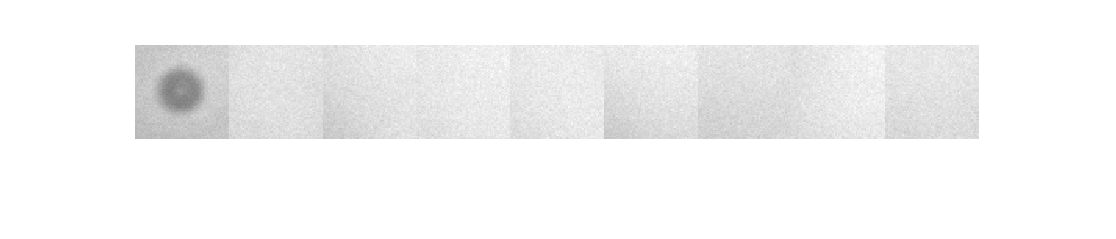

Supplement: Supplementary file 4 — Supplementary Software [file 41467_2023_36373_MOESM4_ESM.zip › analysis software and sample data/CT - Trial Analysis - Sample/Single tethered Bead # 34.tif]

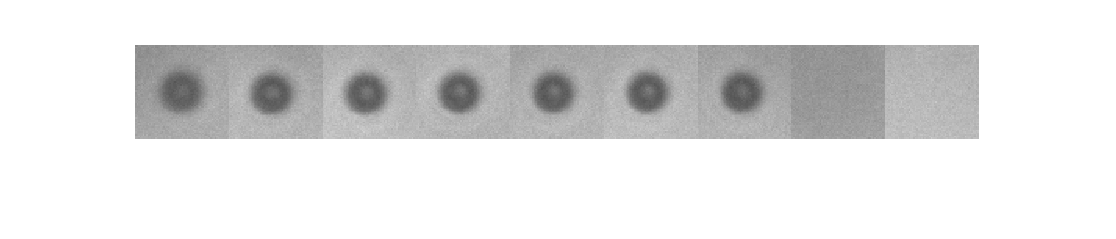

Supplement: Supplementary file 4 — Supplementary Software [file 41467_2023_36373_MOESM4_ESM.zip › analysis software and sample data/CT - Trial Analysis - Sample/Single tethered Bead # 35.tif]

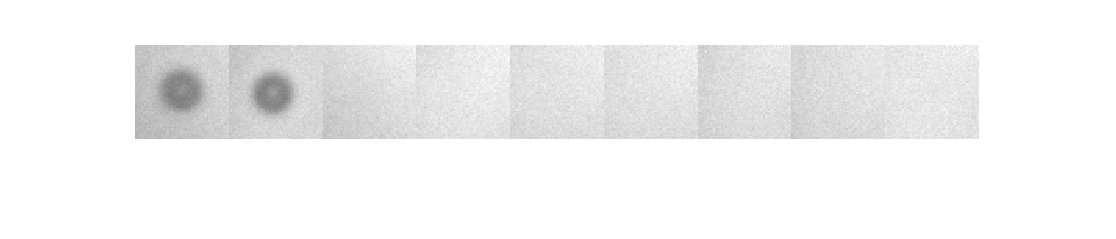

Supplement: Supplementary file 4 — Supplementary Software [file 41467_2023_36373_MOESM4_ESM.zip › analysis software and sample data/CT - Trial Analysis - Sample/Single tethered Bead # 36.tif]

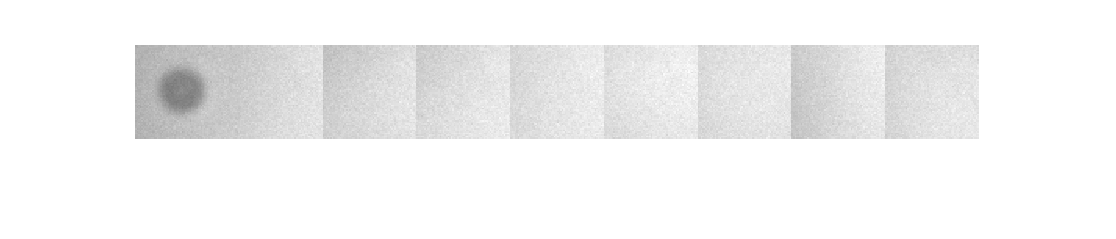

Supplement: Supplementary file 4 — Supplementary Software [file 41467_2023_36373_MOESM4_ESM.zip › analysis software and sample data/CT - Trial Analysis - Sample/Single tethered Bead # 37.tif]

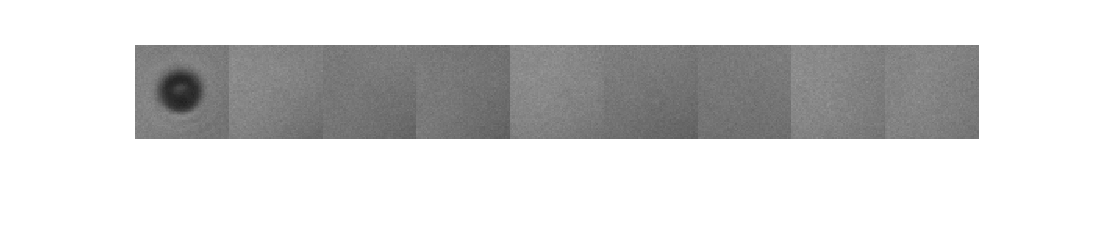

Supplement: Supplementary file 4 — Supplementary Software [file 41467_2023_36373_MOESM4_ESM.zip › analysis software and sample data/CT - Trial Analysis - Sample/Single tethered Bead # 4.tif]

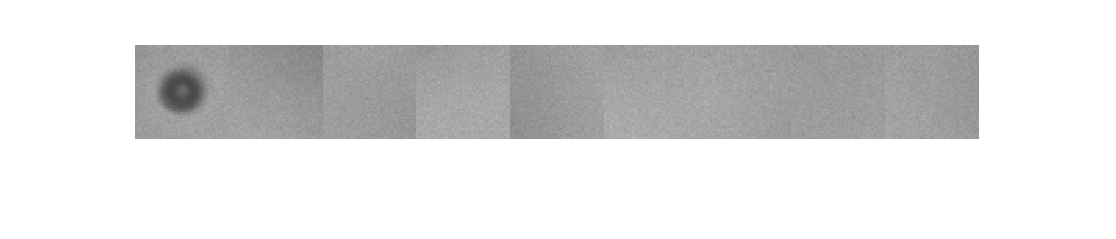

Supplement: Supplementary file 4 — Supplementary Software [file 41467_2023_36373_MOESM4_ESM.zip › analysis software and sample data/CT - Trial Analysis - Sample/Single tethered Bead # 5.tif]

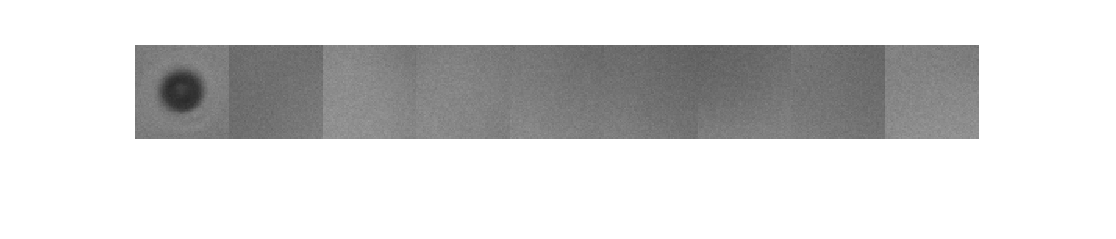

Supplement: Supplementary file 4 — Supplementary Software [file 41467_2023_36373_MOESM4_ESM.zip › analysis software and sample data/CT - Trial Analysis - Sample/Single tethered Bead # 6.tif]

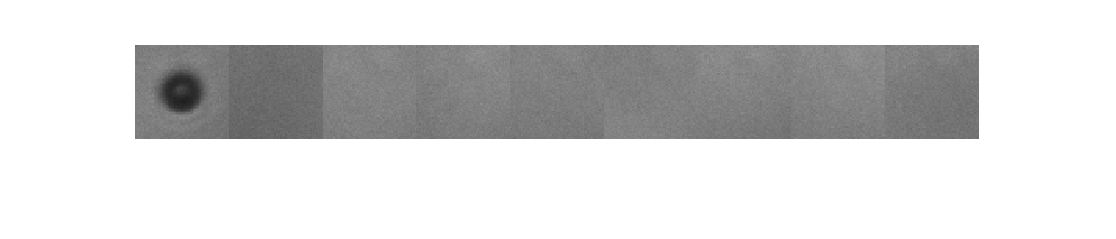

Supplement: Supplementary file 4 — Supplementary Software [file 41467_2023_36373_MOESM4_ESM.zip › analysis software and sample data/CT - Trial Analysis - Sample/Single tethered Bead # 7.tif]

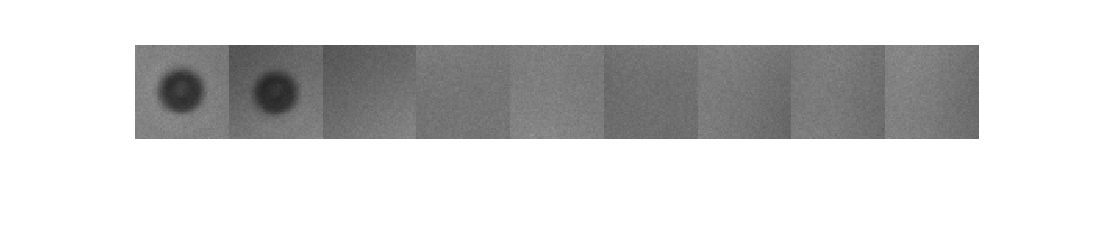

Supplement: Supplementary file 4 — Supplementary Software [file 41467_2023_36373_MOESM4_ESM.zip › analysis software and sample data/CT - Trial Analysis - Sample/Single tethered Bead # 8.tif]

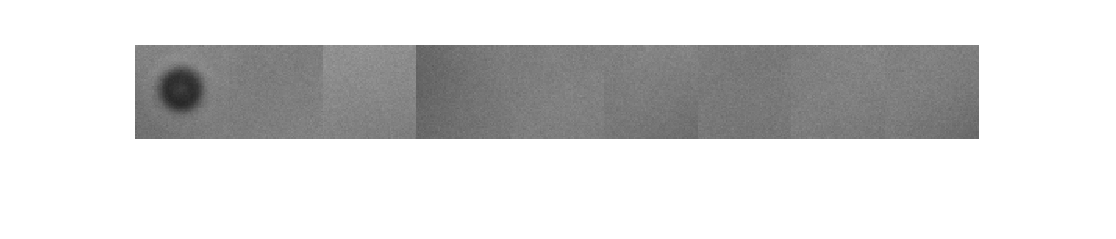

Supplement: Supplementary file 4 — Supplementary Software [file 41467_2023_36373_MOESM4_ESM.zip › analysis software and sample data/CT - Trial Analysis - Sample/Single tethered Bead # 9.tif]
